# A New Method for the Synthesis of 3-Thiocyanatopyrazolo[1,5-*a*]pyrimidines

**DOI:** 10.3390/molecules25184169

**Published:** 2020-09-11

**Authors:** Vladimir A. Kokorekin, Sergey V. Neverov, Vera N. Kuzina, Vladimir A. Petrosyan

**Affiliations:** 1 N. D. Zelinsky Institute of Organic Chemistry, Russian Academy of Sciences, Leninsky prosp. 47, 119991 Moscow, Russia; kokorekin@yandex.ru (V.A.K.); sergei.neverov@yandex.ru (S.V.N.); 2 Institute of Pharmacy, Sechenov First Moscow State Medical University (Sechenov University), Trubetskaya str. 8, bldg. 2, 119991 Moscow, Russia; kuzinavn@mail.ru; 3 All-Russian Research Institute of Phytopathology, Institute str. 5, Bol’shiye Vyazemy, 143050 Moscow, Russia

**Keywords:** thiocyanate group, 5-aminopyrazoles, 1,3-dicarbonyl compounds, pyrazolo[1,5-*a*]pyrimidines, anodic C–H thiocyanation, condensation, cyclic voltammetry

## Abstract

In this article, we demonstrate how an original effective “metal-free” and “chromatography-free” route for the synthesis of 3-thiocyanatopyrazolo[1,5-*a*]pyrimidines has been developed. It is based on electrooxidative (anodic) C–H thiocyanation of 5-aminopyrazoles by thiocyanate ion leading to 4-thiocyanato-5-aminopyrazoles (stage 1, yields up to 87%) following by their chemical condensation with 1,3-dicarbonyl compounds or their derivatives (stage 2, yields up to 96%). This method is equally effective for the synthesis of 3-thiocyanatopyrazolo[1,5-*a*]pyrimidines, both without substituents and with various donor (acceptor) substituents in the pyrimidine ring.

## 1. Introduction

The functionalization of arenes ensures their diversity and opens the way to a wide range of practically useful substances. At present, the important methodology for (hetero)arenes modification is the functionalization of their C–H bonds [1,2,3]. One of the actively developed approaches is the electrooxidative C–H functionalization of (hetero)arenes, using the anode (An) as a “green oxidizing agent” (C–H (An) functionalization) [4,5,6,7].

Our attention was attracted by C–H thiocyanation of (hetero)arenes, which is usually carried out chemically [8,9]. Resulting (hetero)aryl thiocyanates are interesting as precursors of various sulfur-containing compounds, as well as substances with a wide spectrum of bioactivity [8,9,10,11,12,13].

**Scheme 1:**
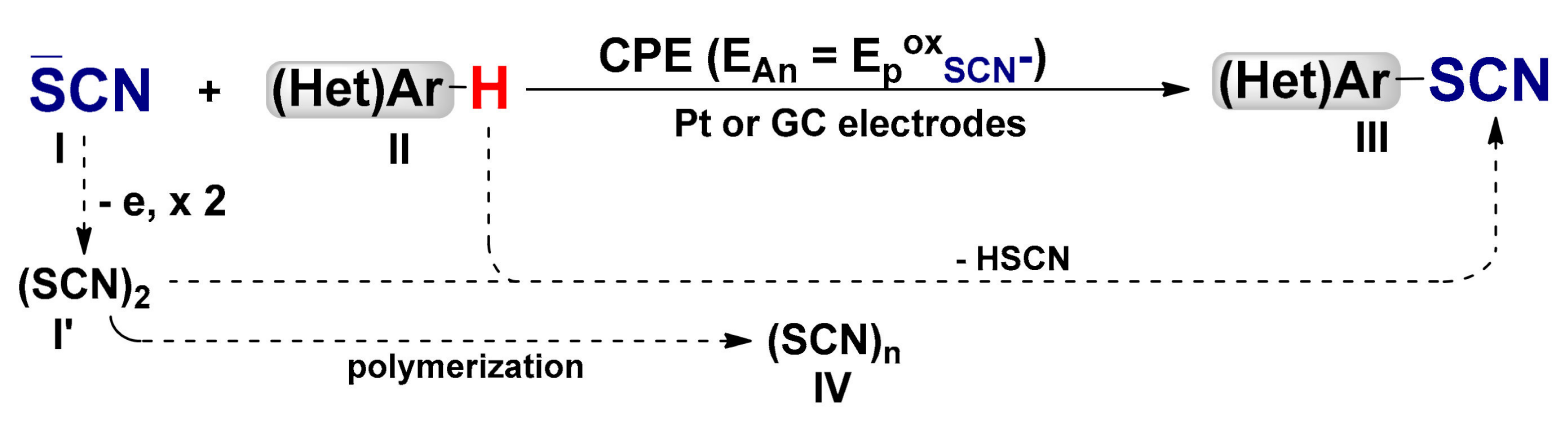
C–H (An) thiocyanation of (hetero)arenes (**II**) via the thiocyanogen (**I′**) at controlled potential electrolysis (CPE) at Е_An_ = E_p_^ox^_SCN_^−^.

Earlier, we realized several processes of anodic C–H thiocyanation of (hetero)arenes [14,15,16,17,18] as part of the development of the C–H (An) functionalization methodology. To obtain target products, a thiocyanate ion (**I**)/(hetero)arene (**II**) mixture in MeCN was subjected to controlled potential electrolysis (CPE) at an anode potential (Е_An_) equal to the oxidation peak potential (E_p_^ox^) of SCN^−^ (Scheme 1) in an undivided cell equipped with Pt or glassy carbon (GC) electrodes. Based on cyclic voltammetry (CV) data and depending on conditions, the Е_An_ was 0.70–0.85 V (vs. SCE). Furthermore, that C–H (An) thiocyanation can be carried out at galvanostatic electrolysis (GE) [14,16,19,20,21].

The key intermediate of such processes is well-known [8,9] thiocyanogen (**I′**), which is electrogenerated at stage **I → I′**. In addition to interaction with arene **II**, leading to aryl thiocyanate **III** (stage (**I′** + **II → III**), thiocyanogen **I′** is capable of polymerization to polythiocyanogen **IV** (stage **I′ → IV**) [14,16,22].

To avoid polymerization, electrolysis can be carried out at low current densities for 1–3 days [21] or at low temperatures [23]. However, we found [14] that thiocyanation in MeCN is successfully realized even at room temperature for 2–4 h—probably due to the ability of MeCN to partially stabilize thiocyanogen **I′ [16]**.

**Scheme 2:**
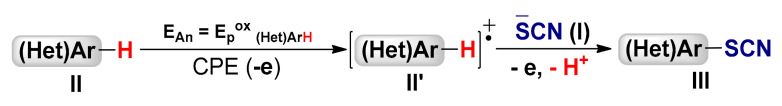
C–H (An) thiocyanation of (hetero)arenes (**II**) via ECE mechanism at CPE at E_An_ = E_p_^ox^_(Неt)ArH_.

On the other hand, it was shown [16,17,18] that CPE of an SCN^−^ (**I**)/(hetero)arene (**II**) mixture can be realized at E_An_ = E_p_^ox^_(Неt)ArH_ (Scheme 2). The process can proceed according to the ECE mechanism [16,18] via the electrogeneration of the radical cation **II′**, leading to the target product **III** in good yield. This is especially valuable when the previous process (Scheme 1) was ineffective.

A similar situation was reported in refs [16,18], where several approaches to the efficient C–H (An) thiocyanation of substituted pyrazolo[1,5-*a*]pyrimidines were first developed. Such structures are of big interest, because pyrazolo[1,5-*a*]pyrimidine is one of the synthetic analogs of purine and a scaffold for many bioactive compounds [24]. During the above studies, initial pyrazolo[1,5-*a*]pyrimidines were preliminarily obtained by condensation of 5-aminopyrazoles with 1,3-dicarbonyl compounds (or their derivatives) under various conditions [15,25,26,27,28,29,30] with very moderate yields in half the cases. On the contrary, C–H (An) thiocyanation of pyrazolo[1,5-*a*]pyrimidines was implemented in good and high yields in all cases [15,16,18].

**Scheme 3:**
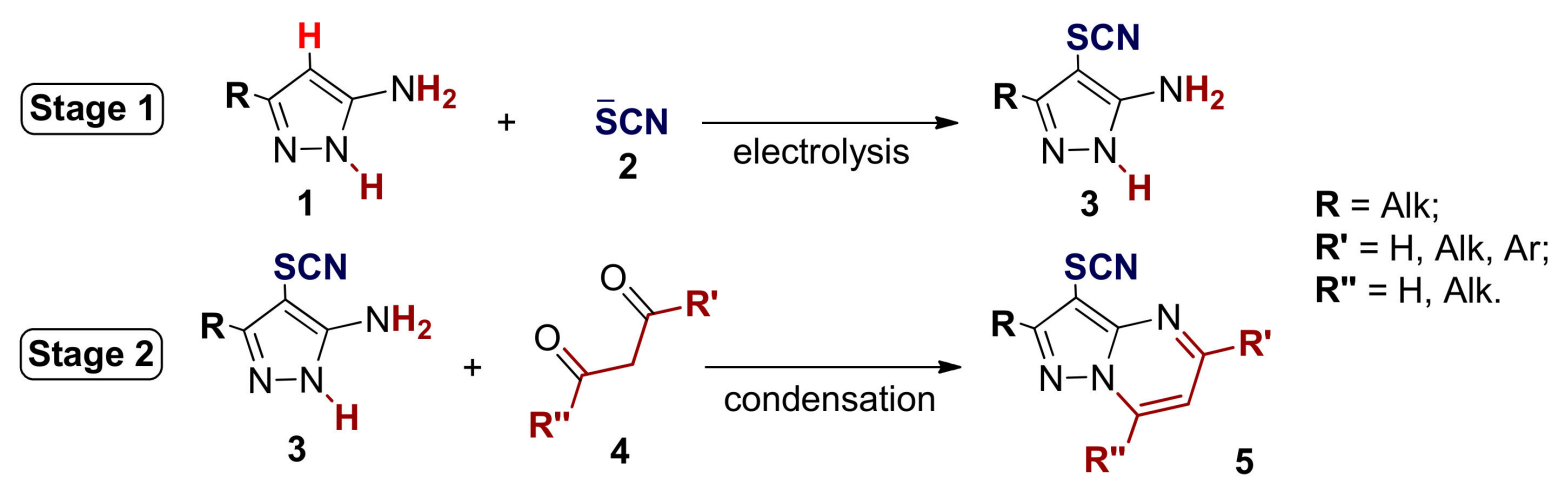
A new approach to the synthesis of 3-thiocyanatopyrazolo[1,5-*a*]pyrimidines **5**: C–H (An) thiocyanation of 5-aminopyrazoles **1** (stage 1) following by condensation of the resulting 4-thiocyanatopyrazoles **3** with a 1,3-dicarbonyl compounds (or their derivatives) **4** (stage 2).

Considering the above, we expected more efficiency from the new approach (Scheme 3), where stage 1 corresponds to C–H (An) thiocyanation of 5-aminopyrazole **1** leading to pyrazole **3**; and stage 2 corresponds to the condensation of pyrazole **3** with a 1,3-dicarbonyl compound (or its derivative) **4** to form the target product **5**. In addition, the condensation of 4-substituted aminopyrazoles usually proceeds in high yields [31,32,33].

Thus, the ultimate goal of the present work was to assess the feasibility and efficiency of the new approach to the synthesis of 3-thiocyanatopyrazolo[1,5-*a*]pyrimidines (Scheme 3).

## 2. Results and Discussion

### 2.1. Anodic C–H Thiocyanation of 5-Aminopyrazoles (Scheme 3, Stage 1)

**Figure 1:**
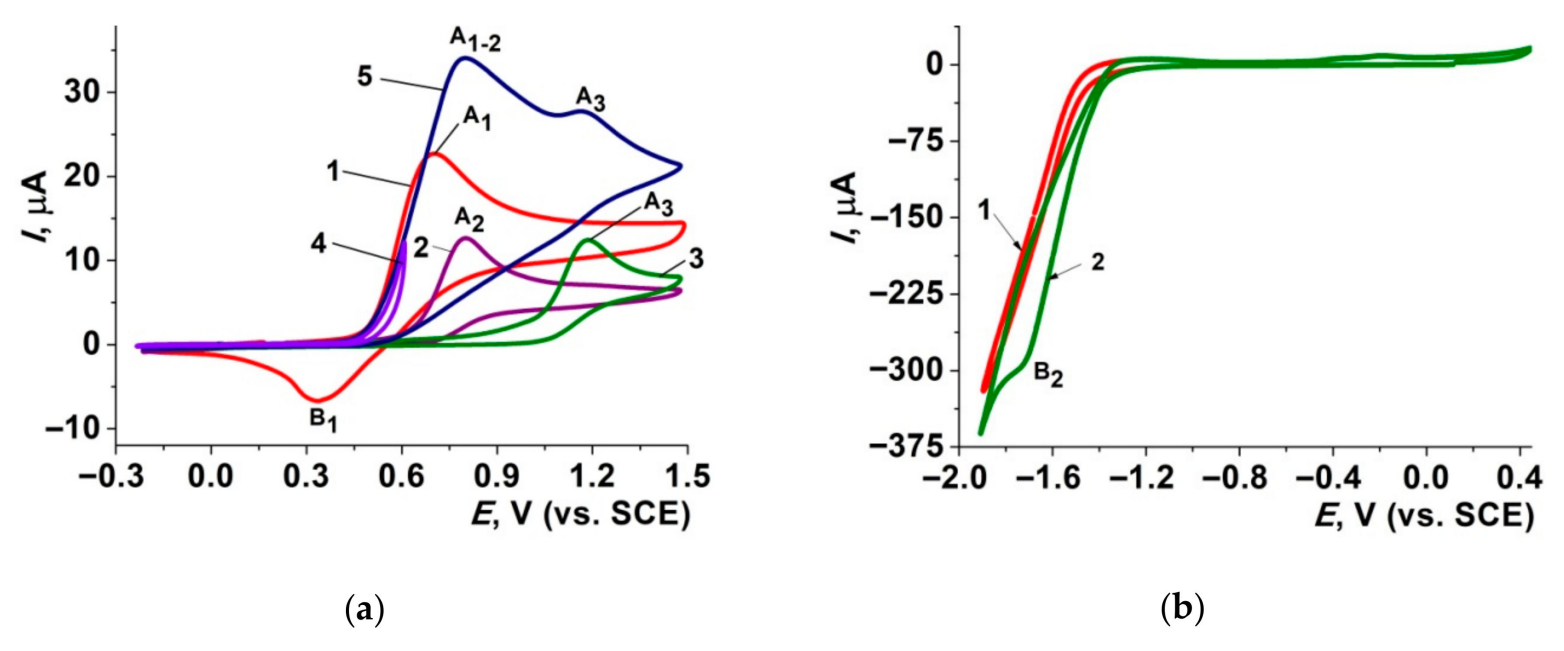
Cyclic voltammetry (CV) curves on Pt working electrode in 0.1M NaClO_4_ in MeCN, ν = 0.10 V·s^−1^. (**a**) NH_4_SCN (0.002 М)—1; azole **1а** (0.002 М)—2; 3-methyl-4-thiocyanato-1*H*-pyrazol-5-amine **3a** (0.002 М)—3; mixture NH_4_SCN/azole **1а** (1:1) with the reverse scan from 0.60 V—4; the same on the reverse scan from 1.45 V—5; (**b**) azole **1а** (0.002 М)—1; thiocyanato-pyrazole **3a** (0.002 М)—2.

Predicting successful C–H (An) thiocyanation of various (hetero)arenes is of obvious interest. We have recently developed the original express-test to find suitable (hetero)arenes for such processes and to evaluate their efficiency without electrolysis [34]. This test is based on an analysis of CV data for thiocyanate ion, (hetero)arenes and their mixtures. Thus, we started the study of stage 1 (Scheme 3) with CV measurements (Figure 1).

#### 2.1.1. CV Data

The model object was the couple thiocyanate ion/3-methyl-1*H*-pyrazol-5-amine (**1a**) (previously not studied in either the electrochemical or chemical C–H thiocyanation). Figure 1a shows a typical CV of the SCN^−^ ion (curve 1) with a one-electron oxidation peak **A_1_** (E_p_^ox^ = 0.70 V). Its irreversibility is due to the formation of thiocyanogen **I′** (see Scheme 1, stage **I**→**I′**), which was observed on the reverse scan as the reduction peak **B_1_** (E_p_^red^ = 0.34 V) [14,16,23,34]. The changes in peak **B_1_** after adding of (hetero)arene to the thiocyanate ion solution is the basis of the above express-test: If the peak **B_1_** did not change, then (hetero)arene does not react with thiocyanogen **I′**, whereas if the peak **B_1_** decreased or disappeared, then the (hetero)arene reacts with thiocyanogen **I′** (see stage **I′** + **II → III**). Moreover, the more efficient the target process, the lower the peak **B_1_** height.

As follows from Figure 1a, the adding of an equimolar amount of azole **1а** led to a complete disappearance of peak **B_1_** even at a potential reverse from 0.60 V (curve 4). It means almost complete consumption of thiocyanogen **I′**, due to its rapid interaction with azole **1a**. At the same time, peak **B_1_** also disappeared when the potential was reversed from 1.45 V (curve 5). This case is primarily interesting because the peak **A_1_**–**_2_** (E_p_^ox^ = 0.80 V, curve 5) is obviously the peak of the co-oxidation of the SCN ^–^ (E_p_^ox^ = 0.70 V, see curve 1) and azole **1а** (E_p_^ox^ = 0.81 V, see curve 2), since the ΔE_p_^ox^ of these peaks is 0.11 V.

These results indicate the effective interaction of thiocyanogen and azole **1a**, according to Scheme 1 (stage **I′**+ **II →** **III**). At the same time, the nearness of the oxidation potentials of thiocyanate ion and azole **1a** (0.11 V) does not exclude the ECE mechanism (Scheme 2), especially since the anodic oxidation of azole **1a** cannot be excluded even at 0.70 V. Overall, the set of CV data indicates the probability of successful realization of C–H (An) thiocyanation. It was also confirmed by peak **A_3_** (E_p_^ox^ = 1.15 V, curve 5), which corresponds to the oxidation of target product **3а** (cf. with curve 3). Note that 3-cyclopropyl-1*H*-pyrazol-5-amine (**1b**) (an additional research object) had similar CV behavior.

It must be pointed that the C–H (An) thiocyanation of (hetero)arenes is often performed in undivided cells, neglecting the possible cathodic decomposition of the target products, which can affect the processes efficiency. Therefore, we accomplished additional CV studies to evaluate this possibility. Figure 1b shows that the CV curve of the initial azole **1a** in the cathodic region (curve 1) practically coincided with that of the supporting electrolyte, while thiocyanato-pyrazole **3a** gave the clear reduction peak (E_p_^red^ = –1.72 V, curve 2). Taking into account these results, the electrolysis in a divided cell seemed to be more appropriate, since it excluded the possible reduction and, accordingly, decomposition of the target thiocyanato-product.

#### 2.1.2. Effect of Electrolysis Conditions on the Yield of the Target Product

On the example of electrolysis of a thiocyanate ion/azole **1a** mixture, it turned out that process proceeded most efficiently on GC electrodes (which were also effective in thiocyanation of other (hetero)arenes [16,18]) than on commonly used Pt electrodes (Table 1, cf. entries 1 and 2). As a result (entry 1), the maximum yield of product **3a** (83%) was obtained under CPE at Е_An_ = 0.90 V in a divided cell using NH_4_SCN (thiocyanating agent) and 0.1M NaClO_4_ in MeCN-H_2_O (20:1) (supporting electrolyte) after passing the theoretical amount of electricity (Q = Q_t_ = 193 C). Under other conditions, the yield of product **3a** decreased by ~10–40% (entries 2–7).

Therefore, the yield of product **3a** was 72% when using Pt electrodes (entry 2). The resinification was observed in an undivided cell (entry 3), while the yield of the target product was 42%. According to the CV data (see Figure 1b), it can be due to its cathodic decomposition (under the electrolysis conditions, E_Cat_ was –1.8 … –2.7 V). The use of KSCN or NaSCN (entry 4) reduced the yield of the target product to 65%, most likely due to their lower solubility in the MeCN-H_2_O. In the absence of H_2_O (entry 5), azole **1а** was less soluble in the reaction mixture, which also reduced the process efficiency. A resinification and a decrease in the yield of product **3а** to 63% were observed when an increase in Е_An_ to 1.10 V (entry 6). According to the CV data (Figure 1a, curve 3), it can be due to the electrooxidation of product **3а** at this potential. On the contrary, a decrease in Е_An_ to 0.70 V had almost no effect on the yield of the target product (cf. entries 7 and 1), but increased the electrolysis duration from ~2.5 h to ~3.5 h.

#### 2.1.3. Synthesis of Target Products

Under optimal conditions, along with the target azole **3a** (yield 83%), azole **3b** (yield 87%) was also obtained (Table 2, entries 1 and 4). An attempt to scaling up the process with a 5-fold increase in the loading of the starting reagents was successful. The yield of products **3a** and **3b** was 74–78% under CPE (entries 2 and 5) and 69–71% under GE (entries 3 and 6) at the full conversion of azoles **1a** and **1b**.

Thus, for the first time, the CV method made it possible to sufficiently simulate the thiocyanation process and evaluate the participation of the starting pyrazole and the target product in both the anodic and cathodic processes. This led to the successful realization of an efficient “metal-free” C–H (An) thiocyanation of 5-aminopyrazoles under CPE and GE with the possibilities of scaling up these processes and of “chromatography-free” isolation of products **3a** and **3b** in pure form by (re)extraction (see Section 3.2.1andSection 3.2.3

### 2.2. Condensation of 4-Thiocyanato-5-Aminopyrazoles with 1,3-Dicarbonyl Compounds (or their Derivatives) (Scheme 3, Stage 2)

As noted above, the known [15,25,26,27,28,29,30] methods of condensation of 5-aminopyrazoles differ. Therefore, we developed a more universal and effective method at this stage.

#### 2.2.1. Effect of Conditions on the Target Product Yield

On the example of thiocyanato-pyrazole **3a** and diacetal **4a** couple, it turned out that the condensation most efficiently (Table 3, entry 1) proceeded in H_2_O in the presence of HCl as a catalyst for 24 h (yield of the product **5aa** 77%). Under other conditions, the yield of thiocyanate **5aa** decreased by ~10–75% (entries 2–7).

In the absence of HCl (entry 2), product **5aa** was almost not formed. When AcOH (entry 3) or H_2_SO_4_ (entry 4) were used, the yield of thiocyanate **5aa** was only 36–39%, and in the latter case, with the simultaneous resinification. Obviously, condensation proceeds at a low rate in the presence of weaker AcOH, while under the action of stronger H_2_SO_4_, along with condensation, partial decomposition of the substrate **3a** (or product **5aa**) occur. An increase in the HCl concentration (entry 5) had practically no effect on the thiocyanate **5aa** yield. It follows from entries 6 and 7 that condensation can also proceed quietly in other media (aqueous EtOH or EtOH). Despite the lower yield of the product **5aa** (65–67%), such media can be widely used in the case of poorly water-soluble 1,3-dicarbonyl compounds (or their derivatives).

#### 2.2.2. Synthesis of Target Products

Based on the above results, we synthesized the series of 3-thiocyanatopyrazolo[1,5-*a*]pyrimidines both without substituents and with various donor (acceptor) substituents in the pyrimidine ring (Table 4).

Experiments with the diacetal **4a** and diketone **4b** (entries 1–4) were proceeded rather effective in an H_2_O with 77–96% yields of products **5aa**–**bb**. The less water-soluble hemiacetals **4c,d**, and diketones **4e,f** (entries 5–8) were most efficiently condensed in aqueous EtOH (yield of thiocyanates **5ac**–**af** was 71–89%). Finally, the processes involving poorly water-soluble diketones **4g** and **4h** (entries 9 and 10) were most successfully carried out in EtOH with a yield of 84–91%.

Thus, a sufficiently universal method for the condensation of 5-aminopyrazoles with 1,3-dicarbonyl compounds and their derivatives was developed. As a result, we obtained a series of target 3-thiocyanatopyrazolo[1,5-*a*]pyrimidines, as well as worked out their “chromatography-free” isolation in pure form by precipitation from the reaction mixture (see subSection 3.3.2).

## 3. Materials and Methods

### 3.1. General Information

The ^1^H and ^13^C-NMR spectra were recorded in CDCl_3_ and DMSO-d_6_ on a Bruker Avance 300 (Bruker BioSpin GmbH, Karlsruhe, Germany) instrument (300.1 MHz for ^1^H and 75.5 MHz for ^13^C), Bruker Avance DRX-500 (Bruker Biospin GmbH, Rheinstetten, Germany) instrument (125.8 MHz for ^13^C) and Bruker Avance AV600 (Bruker Biospin GmbH, Rheinstetten, Germany) instrument (600.1 MHz for ^1^H and 150.9 MHz for ^13^C). The chemical shifts values (δ) were expressed relative to the chemical shifts of the solvent-*d*. High resolution mass-spectra (HRMS) were measured on the Bruker micrOTOF II instrument (Bruker Daltonics, Bremen, Germany) using electrospray ionization (ESI). Melting points were determined on Gallenkamp melting point apparatus MFB-595-010M (Weiss-Gallenkamp, London, UK) and they are uncorrected. MeCN (99.9%, for HPLC), EtOH (95%, for analysis), water (for analysis), toluene (99+%, extra pure), EtOAc (99+%, extra pure), NH_4_SCN (**2**) (99+%, extra pure), KSCN (98%, pure), NaSCN (98%, extra pure), NaClO_4_ (98%, extra pure), Na_2_SO_4_ (99%, extra pure, anhydrous), HCl (32% solution in water, for analysis,), H_2_SO_4_ (96% solution in water, for analysis,), AcOH (99.6%, for analysis), 1,1,3,3-tetraethoxypropane (97%) (**4a**), 2,4-pentanedione (99+%) (**4b**), 1,1,1-trifluoro-2,4-pentanedione (98%) (**4e**), 4,4,4-trifluoro-1-phenyl-1,3-butanedione (99%) (**4g**), 4,4,4-trifluoro-1-(2-thienyl)-1,3-butanedione (99%) (**4h**) (Acros Organics, Geel, Belgium) were used as purchased. 3-Methyl-1*H*-pyrazol-5-amine (**1a**), 3-cyclopropyl-1*H*-pyrazol-5-amine (**1b**), 1,1,1-trichloro-4-ethoxybut-3-en-2-one (**4c**), 4-ethoxy-1,1,1-trifluorobut-3-en-2-one (**4d**), 1-cyclopropyl-4,4,4-trifluorobutane-1,3-dione (**4f**) were prepared using reported [35,36,37,38] procedures. More spectral data can be found at Supplementary Materials section.

### 3.2. Anodic C–H Thiocyanation of 5-Aminopyrazoles (Scheme 3, Stage 1)

Voltammetric (CV) studies were carried out in a temperature-controlled (25 °C) glass cell (V = 10 mL) under argon using a P30JM potentiostat (Elins, Moscow Region, Chernogolovka, Russia). The scan rate was 0.10 V·s^–1^. A platinum disc 1 mm in diameter was used as the working electrode. A saturated calomel electrode (SCE) separated from the solution being studied by a salt bridge filled with the supporting electrolyte (0.1M NaClO_4_ in MeCN) was used as the reference electrode. A platinum plate (S = 3 cm^2^) was used as the counter electrode. All experiments were performed with the concentration of studied compounds of 0.002M in MeCN.

Controlled potential electrolyses (CPE) or galvanostatic electrolyses (GE) were carried out using the above potentiostat in a glass temperature-controlled (20–25 °C) cells: Cell A (undivided, *V* = 60 mL, equipped with plane glassy carbon (GC) electrodes, S_An_ = 8 cm^2^, S_Cat_ = 4 cm^2^), cell B (divided with the 3-layer tracing-paper diaphragm, V_An compartment_ = 50 mL, V_Cat compartment_ = 10 mL, equipped with the above GC electrodes), cell C (the above cell, but equipped with the coaxial cylindrical Pt electrodes, S_An_ = 16 cm^2^, S_Cat_ = 10 cm^2^) or cell D (divided with the above diaphragm, V_An comp._ = 85 mL, V_Cat comp._ = 15 mL, equipped with plane GC electrodes, S_An_ = 16 cm^2^, S_Cat_ = 8 cm^2^).

#### 3.2.1. Effect of Electrolysis Conditions on the Yield of the Target Product

Azole **1a** (1 mmol, 0.10 g) and thiocyanating agent (4 mmol, 0.30–0.39 g, NH_4_SCN, KSCN or NaSCN) were dissolved in the corresponding volume of supporting electrolyte (0.1M NaClO_4_ in MeCN-H_2_O (20:1) or MeCN) using cell A or B or C. CPE was performed by passing 2F (Q = 193 C) of electricity (based on 1F per mol NH_4_SCN or 2F per mol of azole **1a**) at E_An_ = 0.70–1.10 V (vs. SCE). After stopping the electrolysis, the solvent was evaporated in vacuo, and residue was extracted with toluene (5 × 25 mL). Further drying of the combined extracts over Na_2_SO_4_, filtration, and evaporation in vacuo gave a pure product **3a** (yield 42–83%, see Table 1).

#### 3.2.2. Anodic Thiocyanation of Azoles **1a**,**b**

Azole **1a,b** (1 mmol, 0.10–0.12 g) and NH_4_SCN **2** (4 mmol, 0.30 g) were added to anodic compartment of cell B with 0.1 M solution of NaClO_4_ in MeCN-H_2_O (20:1) (50 mL). The cathodic compartment contains 0.1 M solution of NaClO_4_ in MeCN-H_2_O (20:1) (10 mL). CPE was performed by passing 193 C of electricity at E_An_ = 0.90 V, then target products **3a,b** were isolated as described above (yield 83–87%, 0.13–0.16 g, see Table 2, entries 1, 4).

#### 3.2.3. Anodic Thiocyanation of Azoles **1a**,**b** on a Larger Scale

Azole **1a,b** (5 mmol, 0.49–0.62 g) and NH_4_SCN **2** (20 mmol, 1.52 g) were added to anodic compartment of cell D with 0.1M solution of NaClO_4_ in MeCN-H_2_O (20:1) (85 mL). The cathodic compartment contains 0.1M solution of NaClO_4_ in MeCN-H_2_O (20:1) (15 mL). Electrolysis was performed by passing 965 C of electricity at E_An_ = 0.90 V (CPE) or I_An_ = 0.02 A (GE). After stopping the electrolysis, the solvent was evaporated in vacuo, the residue was extracted with EtOAc (5 × 50 mL) followed by concentration of the combined extracts in vacuo and re-extraction with toluene (5 × 50 mL). Further drying of the combined extracts over Na_2_SO_4_, filtration, and evaporation in vacuo gave a pure products **3a,b** (yields 69–78%, 0.53–0.70 g, see Table 2, entries 2, 3, 5, 6).

*3-Methyl-4-thiocyanato-1H-pyrazol-5-amine* (**3a**) 83%. Colorless powder. M.p. 120–122 °С. ^1^H-NMR (600.13 MHz, DMSO-*d*_6_): δ 2.16 (s, 3H, Me), 3.56 (s, 2H, NH_2_). ^13^C-NMR (125.8 MHz, DMSO-*d*_6_): δ 13.9, 79.1, 112.5, 146.6, 154.0. HRMS (ESI) calc. for [C_5_H_7_N_4_S]^+^ [M + H]^+^. 155.0386, found 155.0393.

*3-Cyclopropyl-4-thiocyanato-1H-pyrazol-5-amine* (**3b**) 87%. Thick brownish oil. ^1^H-NMR (300.1 MHz, CDCl_3_): δ 0.50–1.42 (m, 4H), 1.84–2.53 (m, 1H), 4.71 (br. s, 2H, NH_2_). ^13^C-NMR (150.9 MHz, CDCl_3_): δ 7.6, 8.2, 8.6, 81.4, 111.3, 147.2, 151.1. HRMS (ESI) calc. for [C_7_H_9_N_4_S]^+^ [M + H]^+^ 181.0542, found 181.0542.

### 3.3. Condensation of 4-Thiocyanato-5-Aminopyrazoles with 1,3-Dicarbonyl Compounds (or Their Derivatives) (Scheme 3, stage 2)

Condensations were carried out in a temperature-controlled (20–25 °C) glass reactor (V = 20 mL).

#### 3.3.1. Effect of Condensation Conditions on the Yield of the Target Product

Azole **3a** (5 mmol, 0.77 g) was dissolved in H_2_O or H_2_O-EtOH (1:4) or EtOH (15 mL), then HCl (2.5 mL or 10 mL), or AcOH or H_2_SO_4_ (2.5 mL) and diacetal **4a** (6 mmol, 1.32 g) were added. After stirring for 24 h, EtOH (entries 6 and 7) was evaporated in vacuo, and the resulting mixture was extracted by EtOAc (5 × 20 mL). The combined extracts were dried over Na_2_SO_4_, filtered, and concentrated in vacuo followed by separation with column chromatography on SiO_2_ (eluent—light petroleum ether/EtOAc mixtures). Yield of product **5aa** was ~0–77% (~0–0.73 g, see Table 3).

#### 3.3.2. Synthesis of Target Products

Azole **3a,b** (5 mmol, 0.77–0.90 g) was dissolved in 15 mL H_2_O (Table 4, entries 1–4) or H_2_O-EtOH (1:4) (entries 5–8) or EtOH (entries 9, 10), then 2.5 mL of 32% aqueous HCl and 1,3-dicarbonyl compound (or its derivative) **4a**–**h** (6 mmol, 0.60–1.33 g) were added. After stirring for 24 h, EtOH (entries 5–10) was evaporated in vacuo, and H_2_O (entries 9 and 10) was added. Washing of the formed precipitate with H_2_O (4 × 15 mL) gave the pure target products **5aa**–**h**, **5ba**–**b** (yields 71–96%, 0.73–1.55 g).

*2-Methyl-3-thiocyanatopyrazolo[1,5-a]pyrimidine* (**5aa**) 77%. Colorless powder. M.p. 124–126 °С. ^1^H-NMR (600.1 MHz, CDCl_3_): δ 2.65 (s, 3H, Me), 6.98 (dd, 1H, ^3^*J* = 4.3 Hz, ^3^*J* = 5.2 Hz, H6), 8.57–8.82 (m, 2H, H5, H7). ^13^C-NMR (75.5 MHz, CDCl_3_): δ 13.0, 85.8, 109.4, 110.6, 135.7, 149.6, 151.9, 158.9. HRMS (ESI) calc. for [C_8_H_7_N_4_S]^+^ [M + H]^+^ 191.0386, found 191.0391.

*2-Cyclopropyl-3-thiocyanatopyrazolo[1,5-a]pyrimidine* (**5ba**) 84%. Colorless powder. M.p. 135–138 °С. ^1^H-NMR (300.1 MHz, CDCl_3_): δ 1.12–1.51 (m, 4H), 2.26–2.49 (m, 1H), 6.94 (dd, 1H, ^3^*J* = 6.9 Hz, ^3^*J* = 4.0 Hz, H6), 8.58 (d, 1H, ^3^*J* = 6.9 Hz, H7), 8.64 (d, 1H, ^3^*J* = 4.0 Hz, H5). ^13^C-NMR (150.9 MHz, CDCl_3_): δ 8.9, 10.4, 86.3, 109.9, 111.5, 136.4, 151.0, 152.2, 164.5. HRMS (ESI) calc. for [C_10_H_8_N_4_NaS]^+^ [M + Na]^+^ 239.0362, found 239.0361.

*2,5,7-Trimethyl-3-thiocyanatopyrazolo[1,5-a]pyrimidine* (**5ab**) 96%. Colorless powder. M.p. 143–145 °C (lit. 143–144 °C [16]). ^1^H-NMR (300.1 MHz, CDCl_3_): δ 2.62 (s, 3H, Me), 2.63 (s, 3H, Me), 2.72 (s, 3H, Me), 6.69 (s, 1H, H6). ^13^C-NMR (75.5 MHz, CDCl_3_): δ 13.0, 16.8, 24.9, 84.1, 110.1, 111.2, 146.3, 149.7, 157.9, 161.9. HRMS (ESI) calcd. for [C_10_H_10_N_4_NaS]^+^ [M + Na]^+^ 241.0518, found 241.0519.

*2-Cyclopropyl-5,7-dimethyl-3-thiocyanatopyrazolo[1,5-a]pyrimidine* (**5bb**) 92%. Yellowish powder. M.p. 143–146 °C (lit. 143–146 °C [16]). ^1^H-NMR (300.1 MHz, CDCl_3_): δ 0.52–1.53 (m, 4H), 1.94–2.45 (m, 1H), 2.66 (s, 6H, Me), 6.66 (s, 1H, H6). ^13^C-NMR (75.5 MHz, CDCl_3_): δ 8.3, 9.2, 16.7, 24.8, 83.7, 109.8, 111.5, 146.3, 149.8, 161.5, 162.5. HRMS (ESI) calcd. for [C_12_H_12_N_4_S]^+^ [M + H]^+^ 245.0855, found 245.0854.

*2-Methyl-3-thiocyanato-7-(trichloromethyl)pyrazolo[1,5-a]pyrimidine* (**5ac**) 89%. Yellow powder. M.p. 112–114 °C (lit. 112–115 °C [16]). ^1^H-NMR (300.1 MHz, CDCl_3_): δ 2.76 (s, 3H, Me), 7.66 (d, ^3^*J* = 4.3 Hz, 1H, H6), 8.83 (d, ^3^*J* = 4.3 Hz, 1H, H5). ^13^C-NMR (75.5 MHz, CDCl_3_): δ 13.3, 88.2, 88.4, 106.3, 110.1, 143.7, 151.0, 151.2, 158.2. HRMS (ESI) calcd. for [C_9_H_6_^35^Cl_3_N_4_S]^+^ [M + H]^+^ 306.9373, found 306.9374.

*2-Methyl-3-thiocyanato-7-(trifluoromethyl)pyrazolo[1,5-a]pyrimidine* (**5ad**) 78%. Yellowish powder. M.p. 140–142 °C (lit. 140–142 °C [16]). ^1^H-NMR (300.1 MHz, CDCl_3_): δ 2.74 (s, 3H, Me), 7.33 (d, ^3^*J* = 4.2 Hz, 1H, H6), 8.82 (d, ^3^*J* = 4.2 Hz, 1H, H5). ^13^C-NMR (125.8 MHz, CDCl_3_): δ 13.1, 88.8, 107.2 (q, ^3^*J* = 4.0 Hz, C6), 109.8, 115.6 (q, ^1^*J* = 275.1 Hz, CF_3_), 134.5 (q, ^2^*J* =38.3 Hz, C7), 150.2, 150.9, 159.7. HRMS (ESI) calcd. for [C_9_H_6_F_3_N_4_S]^+^ [M + H]^+^ 259.0260, found 259.0257.

*2,5-Dimethyl-3-thiocyanato-7-(trifluoromethyl)pyrazolo[1,5-a]pyrimidine* (**5ae**) 71%. Yellowish powder. M.p. 124–127 °С. ^1^H-NMR (300.1 MHz, CDCl_3_): δ 2.70, 2.80 (both s, 3H, Me), 7.18 (с, 1H, H6). ^13^C-NMR (125.8 MHz, CDCl_3_): δ 13.1, 25.2, 87.0, 108.3 (q, ^3^*J* = 3.8 Hz, C6), 110.2, 115.7 (q, ^1^*J* = 275.0 Hz, CF_3_), 133.8 (q, ^2^*J* = 38.0 Hz, C7), 150.1, 159.4, 162.1. HRMS (ESI) calcd. for [C_10_H_8_F_3_N_4_S]^+^ [M + H]^+^ 273.0416, found 273.0414.

*5-Cyclopropyl-2-methyl-3-thiocyanato-7-(trifluoromethyl)pyrazolo[1,5-a]pyrimidine* (**5af**) 87%. Yellowish powder. M.p. 132–135 °C (subl.) (lit. 130–133 °C (subl.) [16]). ^1^H-NMR (300.1 MHz, CDCl_3_): δ 1.21–1.34 (m, 4H), 2.13–2.20 (m, 1H), 2.56 (s, 3H, Me), 7.79 (s, 1H). ^13^C-NMR (125.8 MHz, DMSO-*d*_6_): δ 12.7, 13.0, 17.9, 85.7, 108.1 (q, ^3^*J* = 3.9 Hz, C6), 111.3, 116.0 (q,^1^*J* = 274.6 Hz, CF_3_), 132.0 (q, ^2^*J* = 37.1 Hz, C7), 149.6, 157.9, 168.1. HRMS (ESI) calcd. for [C_12_H_10_F_3_N_4_S]^+^ [M + H]^+^ 299.0573, found 299.0563.

*2-Methyl-5-phenyl-3-thiocyanato-7-(trifluoromethyl)pyrazolo[1,5-a]pyrimidine* (**5ag**) 84%. Yellowish powder. M.p. 155–157 °C (lit. 15–157 °C [16])). ^1^H-NMR (300.1 MHz, DMSO-*d*_6_) δ 2.63 (s, 3H, Me), 7.53–7.71 (m, 3H, Ph), 8.28 (s, 1H), 8.34–8.56 (m, 2H, Ph). ^13^C-NMR (75.5 MHz, DMSO-*d*_6_): δ 12.7, 91.9, 106.1 (q, ^3^*J* = 4.1 Hz, C6), 111.2, 117.4 (q, ^1^*J* = 274.9 Hz, CF_3_), 127.9, 129.2, 132.0, 132.9 (q, ^2^*J* = 37.4 Hz, C7), 134.9, 149.5, 158.0, 158.5. HRMS (ESI) calcd. for [C_15_H_10_F_3_N_4_S]^+^ [M + H]^+^ 335.0573, found 335.0568.

*2-Methyl-3-thiocyanato-5-(thiophen-2-yl)-7-(trifluoromethyl)pyrazolo[1,5-a]pyrimidine* (**5ah**) 91%. Yellow powder. M.p. 184–186 °C (lit. 154–157 °C [16]). ^1^H-NMR (300.1 MHz, CDCl_3_): 2.70 (s, 3H, Me), 7.22 (dd, ^3^*J* = 5.1 Hz, ^3^*J* = 3.7 Hz, 1H, thiofenyl), 7.56 (s, 1H), 7.66 (dd, ^3^*J* = 5.1 Hz, ^4^*J* = 1.1 Hz, 1H, thiofeyl), 7.85 (dd, ^3^*J* = 3.7 Hz, ^4^*J* = 1.1 Hz, 1H, thiofenyl). ^13^C-NMR (75.5 MHz, CDCl_3_): δ 13.2, 87.6, 104.0 (q, ^3^*J* = 4.1 Hz, C6), 110.4, 117.3 (q, ^1^*J* = 275.1 Hz, CF_3_), 129.0, 129.9, 133.0, 134.1 (q, ^2^*J* = 37.8 Hz, C7), 141.2, 150.2, 153.4, 159.8. HRMS (ESI) calcd. for [C_13_H_8_F_3_N_4_S_2_]^+^ [M + H]^+^ 341.0137, found 341.0138.

## 4. Conclusions

Summarizing the above studies, it should be especially noted the key role of cyclic voltammetry, which was successfully first used to predict the effective realization of anodic thiocyanation of the initial (hetero)arene. As a result, at the first stage, we proposed an atom economical “metal-free” C–H (An) thiocyanation of 5-aminopyrazoles. We showed the possibility of its scaling under the potentiostatic and galvanostatic electrolysis with a good yields of the target thiocyanato-pyrazoles.

At the second stage, the efficient method for the condensation of 4-thiocyanato-5-aminopyrazoles with 1,3-dicarbonyl compounds (or their derivatives) was developed, which opened the way to many target 3-thiocyanatopyrazolo[1,5-*a*]pyrimidines, both without substituents and with various donor (acceptor) substituents on the pyrimidine ring (including not previously described).

In general, the successful combination of electrochemical and chemical approaches, mild implementation conditions, the use of available reagents and solvents, scalability, and ease of isolation of target products make this new strategy of the two-stage synthesis of 3-thiocyanatopyrazolo[1,5-*a*]pyrimidines very attractive for further applications.

## Figures and Tables

**Table 1:** Effect of electrolysis conditions on the yield of the target product **3a**.

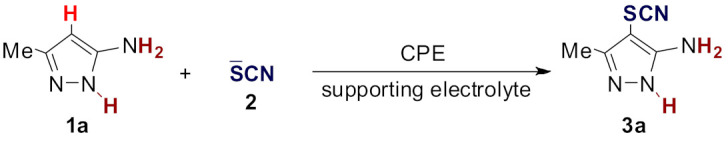		
**Entry**	**Conditions**	**Yield of 3a, %**
1	Optimal ^1^	83
2	Pt electrodes instead of GC	72
3	Undivided cell	42
4	KSCN or NaSCN instead of NH_4_SCN	65
5	MeCN instead of MeCN-H_2_O	61
6	Е_An_ = 1.10 V instead of 0.90 V	63
7	Е_An_ = 0.70 V instead of 0.90 V ^2^	80

^1^ CPE: Glassy carbon (GC) electrodes, divided cell, anolyte (50 mL 0.1 M NaClO_4_ in MeCN-H_2_O (20:1)), azole **1a** (1 mmol), NH_4_SCN (4 mmol)), catholyte (10 mL 0.1 М NaClO_4_ in MeCN-H_2_O), Е_An_ = 0.90 V, Q = Q_t_ = 193 C, T = 2.5 h. The yield is shown for the isolated and purified product **3a**. ^2^ Compared with entry 1, the electrolysis duration increased from ~2.5 h to ~3.5 h.

**Table 2:** C–H (An) thiocyanation of azoles **1a,b**.

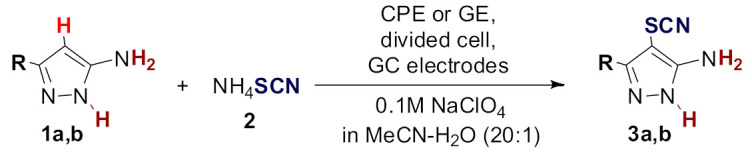			
**Entry**	**Substrate 1**	**Product 3**	**Yield, %**
1 ^1^	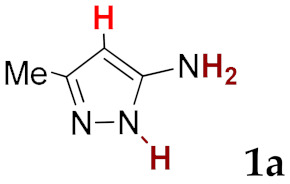	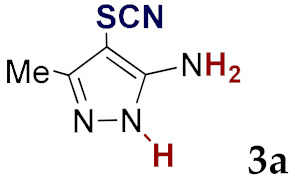	83
2 ^2^			74
3 ^3^			69
4 ^1^	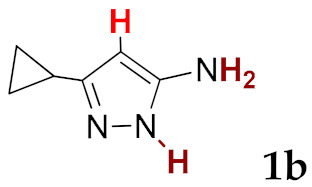	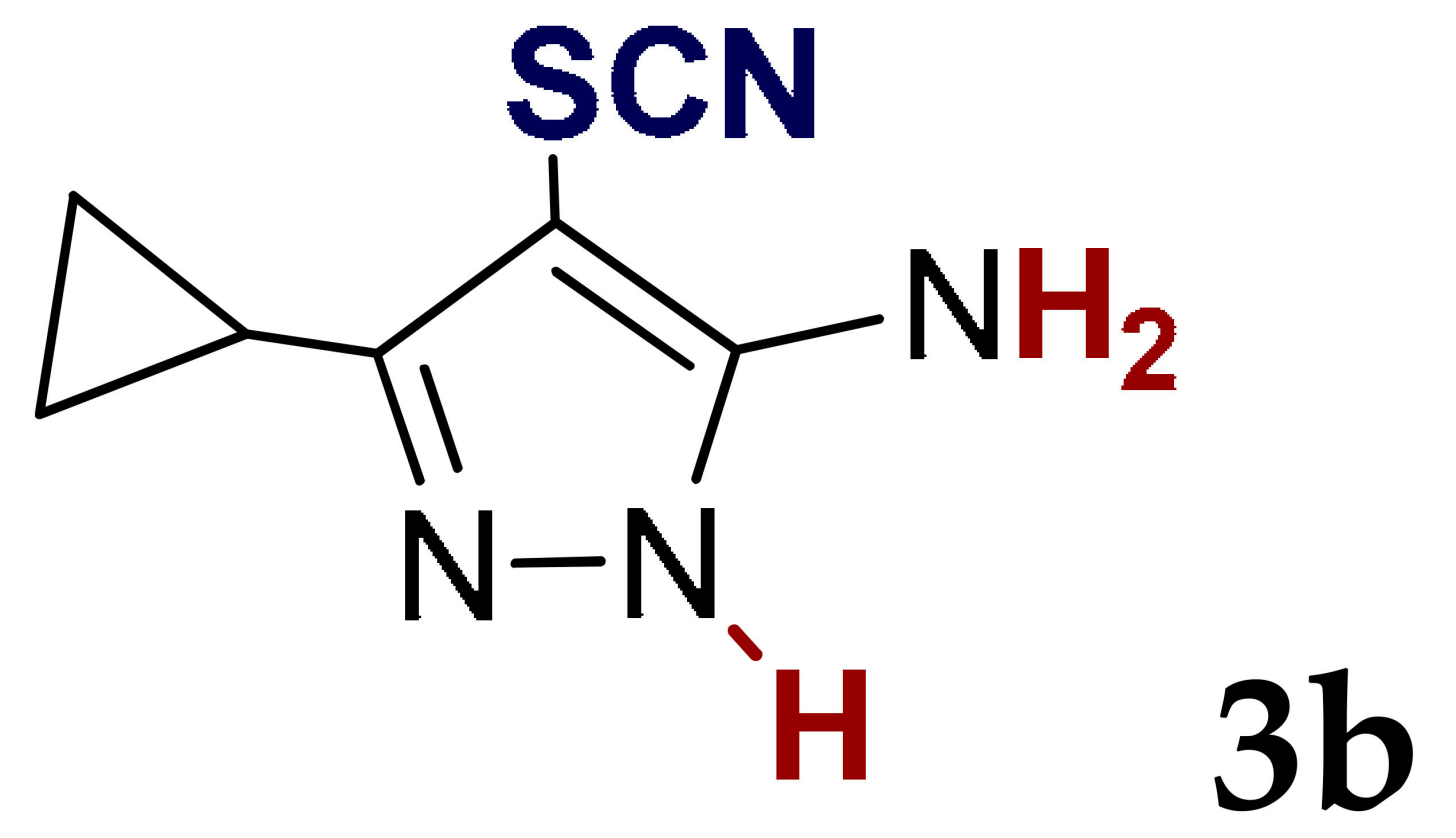	87
5 ^2^			78
6 ^3^			71

^1^ СРЕ: Anolyte (50 mL 0.1М NaClO_4_ in MeCN-H_2_O (20:1), azole **1a,b** (1 mmol), NH_4_SCN **2** (4 mmol)), catholyte (10 mL 0.1 М NaClO_4_ in MeCN-H_2_O), Е_An_ = 0.90 V, Q = Q_t_ = 193 C, T = 2.5 h. Hereinafter, the yield was determined for the isolated target product; ^2^ СРЕ: Anolyte (85 mL 0.1 М NaClO_4_ in MeCN-H_2_O, azole **1a,b** (5 mmol), NH_4_SCN **2** (20 mmol)), catholyte (15 mL 0.1 М NaClO_4_ in MeCN-H_2_O), Е_An_ = 0.90 V, Q = Q_t_ = 965 C, T = 13 h. ^3^ GE at I_An_ = 0.02 A, other conditions see ^2^.

**Table 3:** Effect of the condensation conditions on the yield of the target product **5aa**.

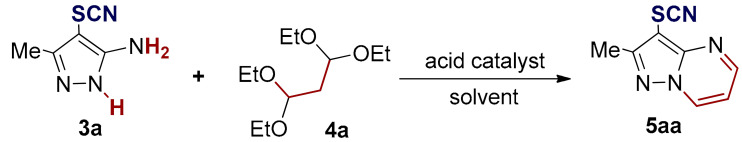		
**Entry**	**Conditions**	**Yield of 5aa, %**
1	Optimal ^1^	77
2	Without HCl	traces
3	AcOH instead of HCl	36
4	H_2_SO_4_ instead of HCl	39
5	5 mL HCl instead of 2.5 mL	75
6	H_2_O-EtOH (1:4) instead of H_2_O	67
7	EtOH instead of H_2_O	65

^1^ Azole **3a** (5 mmol) was dissolved in 15 mL H_2_O, then 2.5 mL of 32% aqueous HCl and diacetal **4a** (6 mmol) were added. The mixture was stirred for 24 h. The yield was determined for the isolated and purified product.

**Table 4:** Condensation of azoles **3a,b** with 1,3-dicarbonyl compounds (or their derivatives) **4a**–**h**.

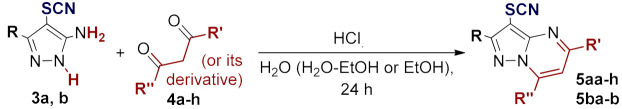				
**Entry**	**Substrate 3**	**Substrate 4**	**Product 5**	**Yield, %**
1 ^1^	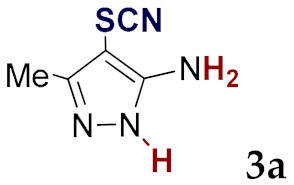	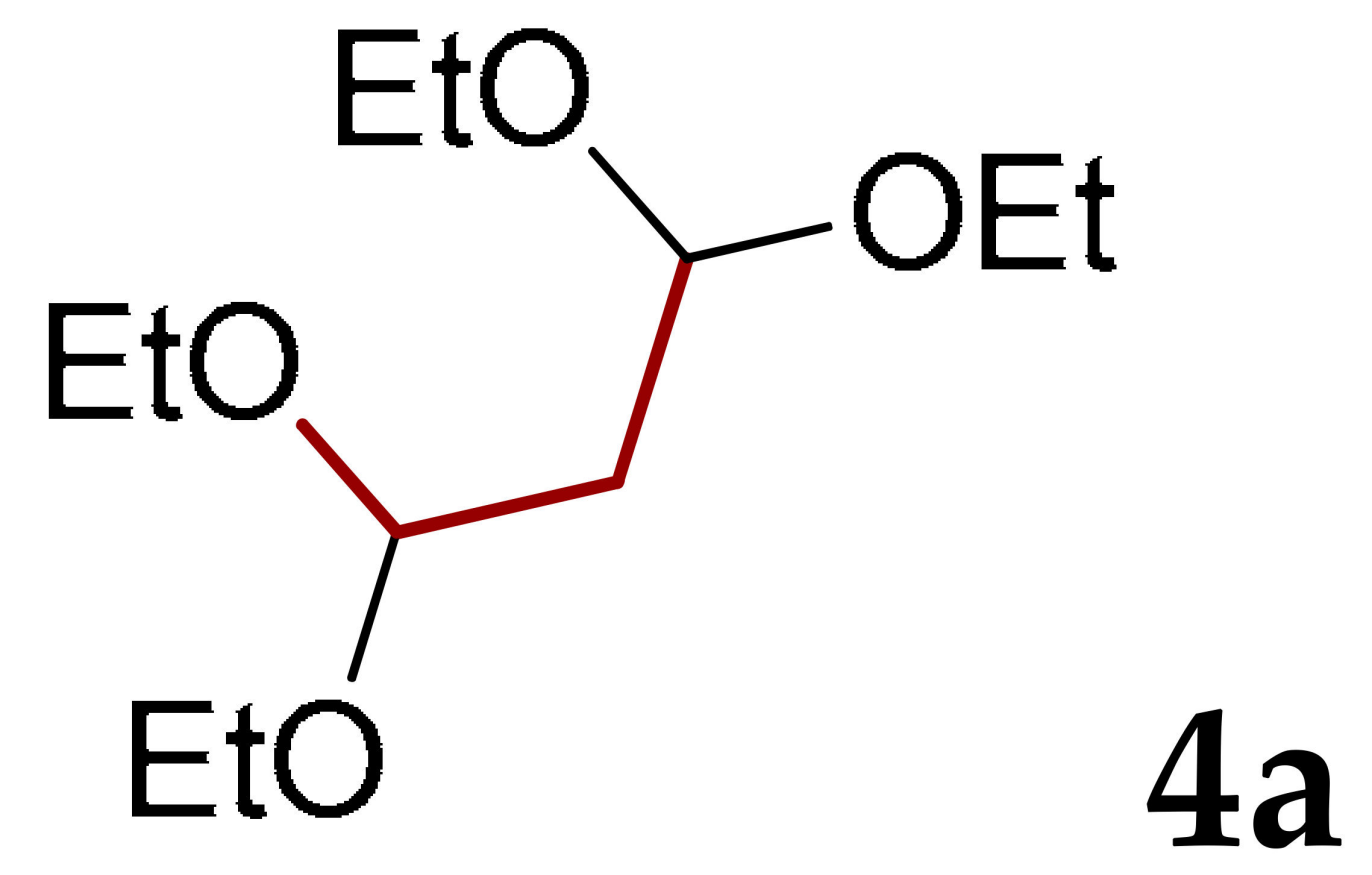	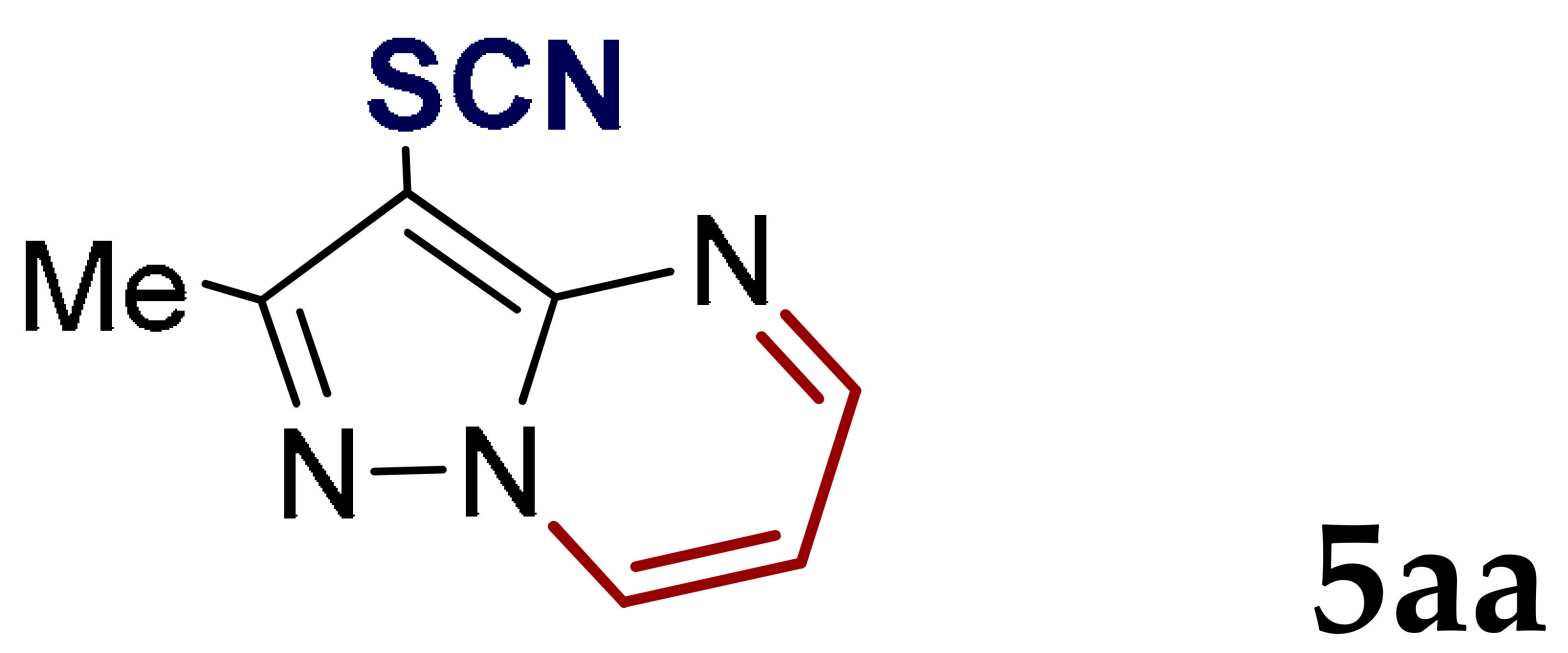	77
2 ^1^	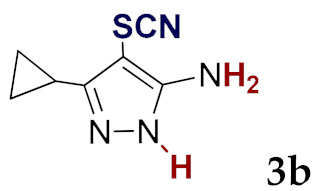	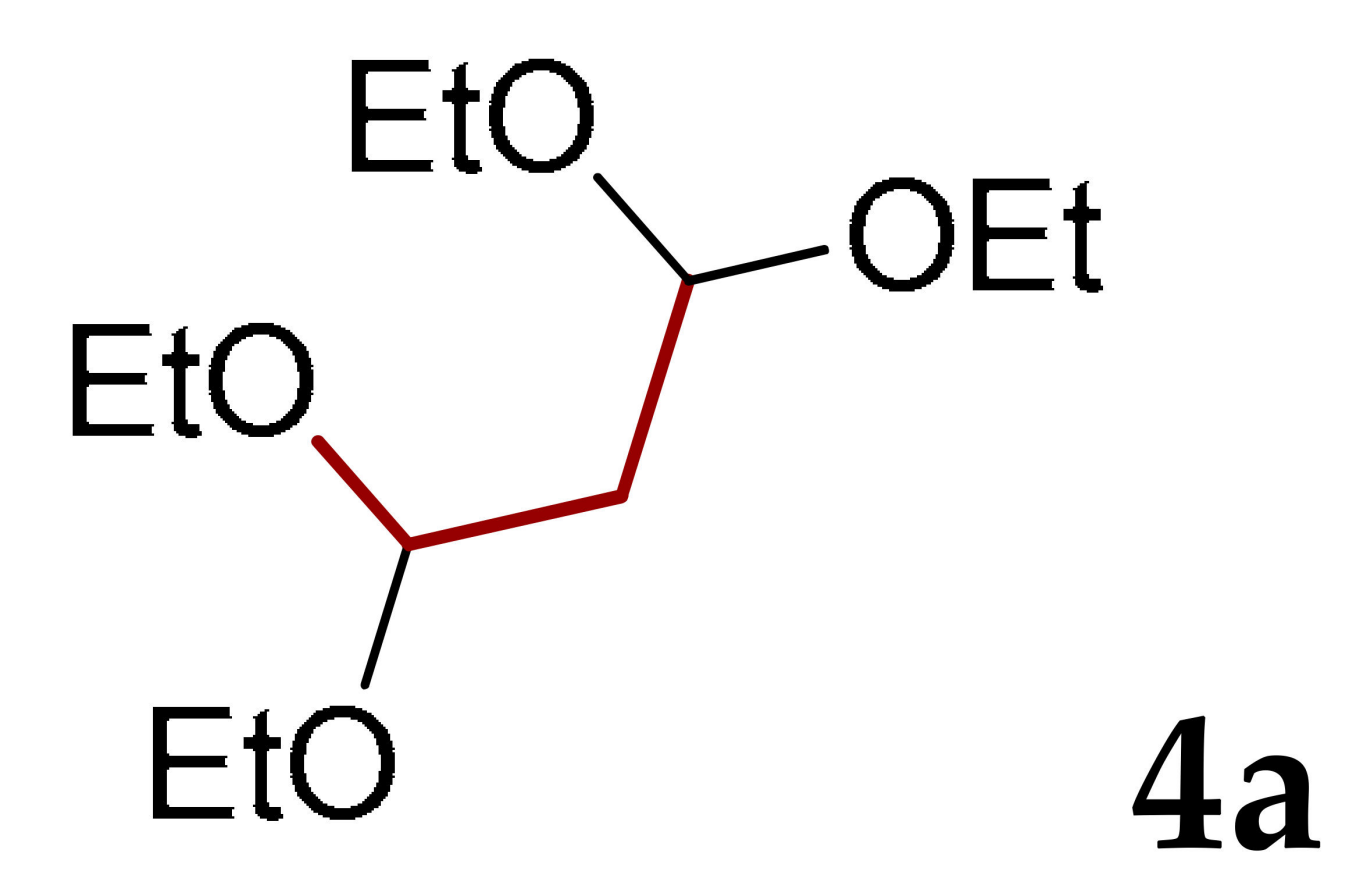	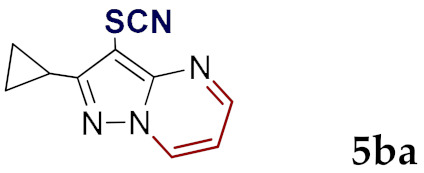	84
3 ^1^	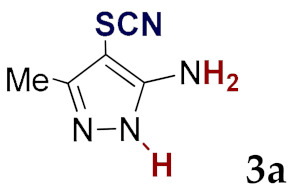	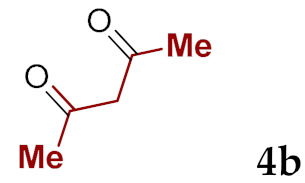	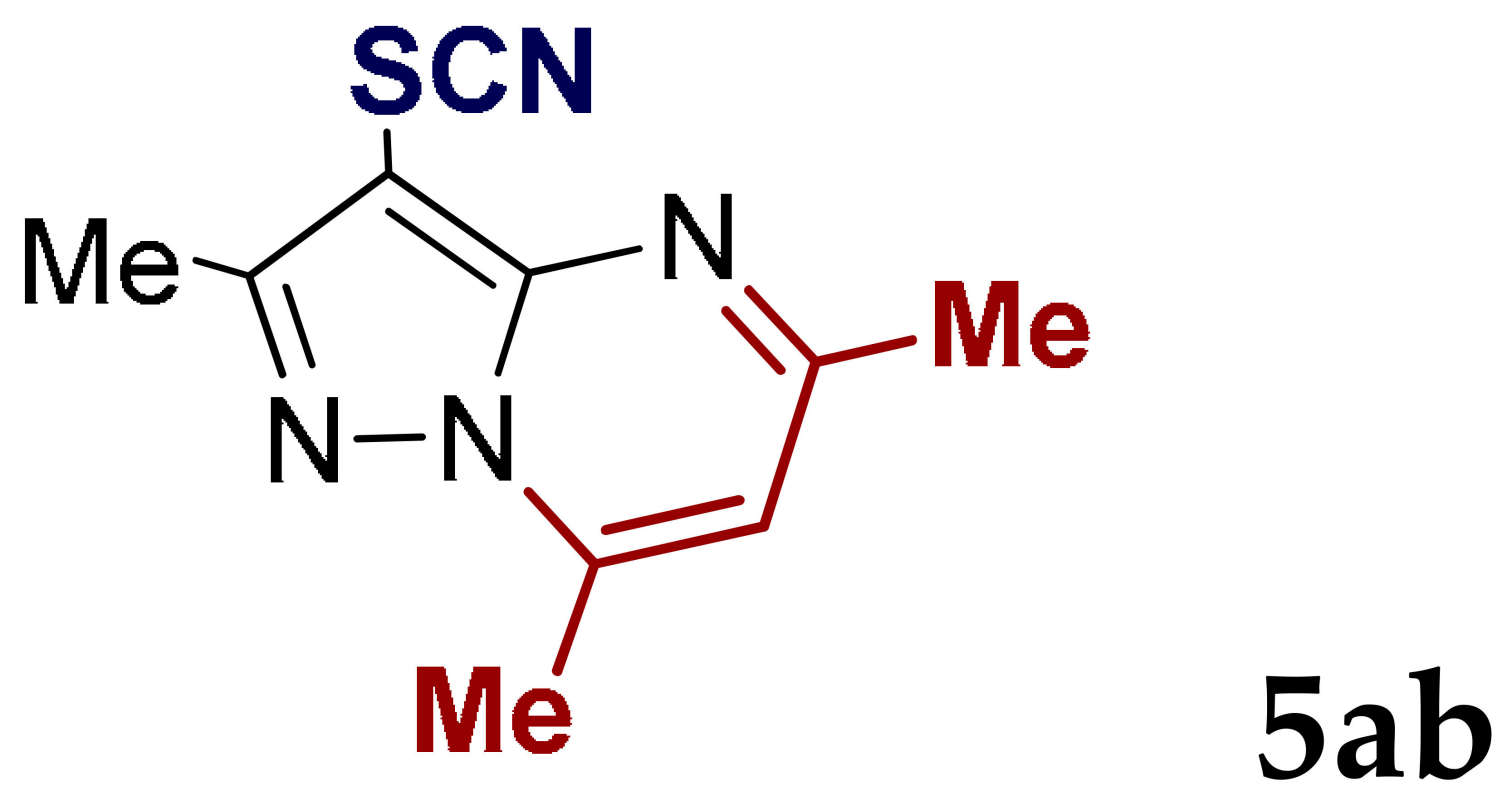	96
4 ^1^	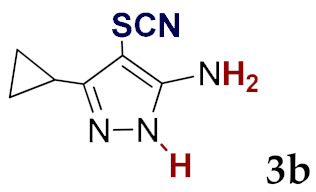	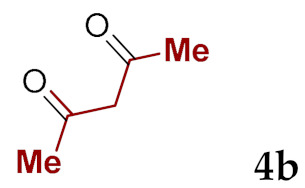	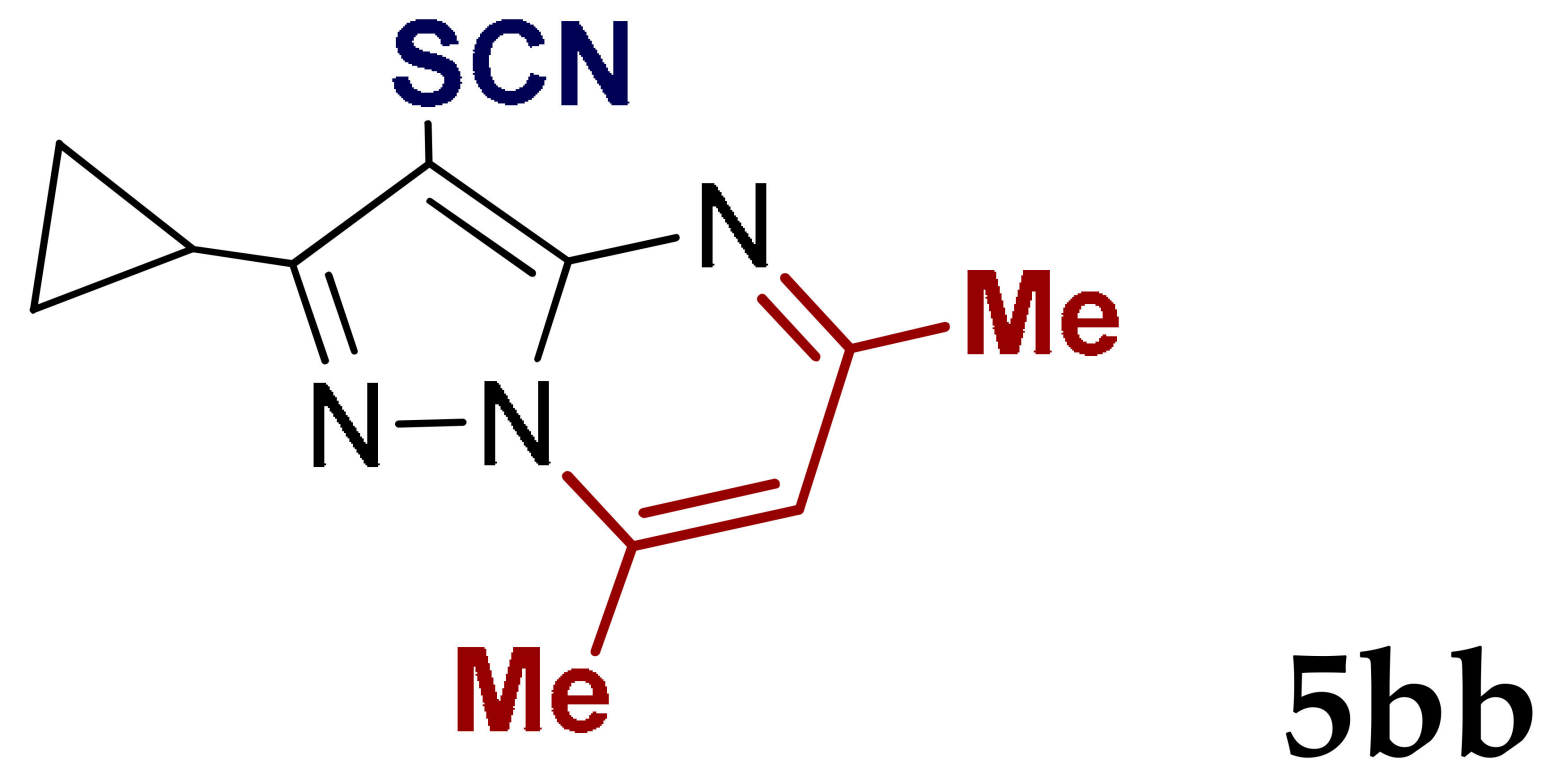	92
5 ^2^	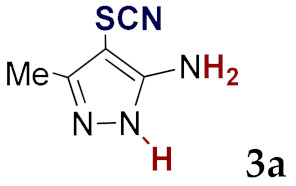	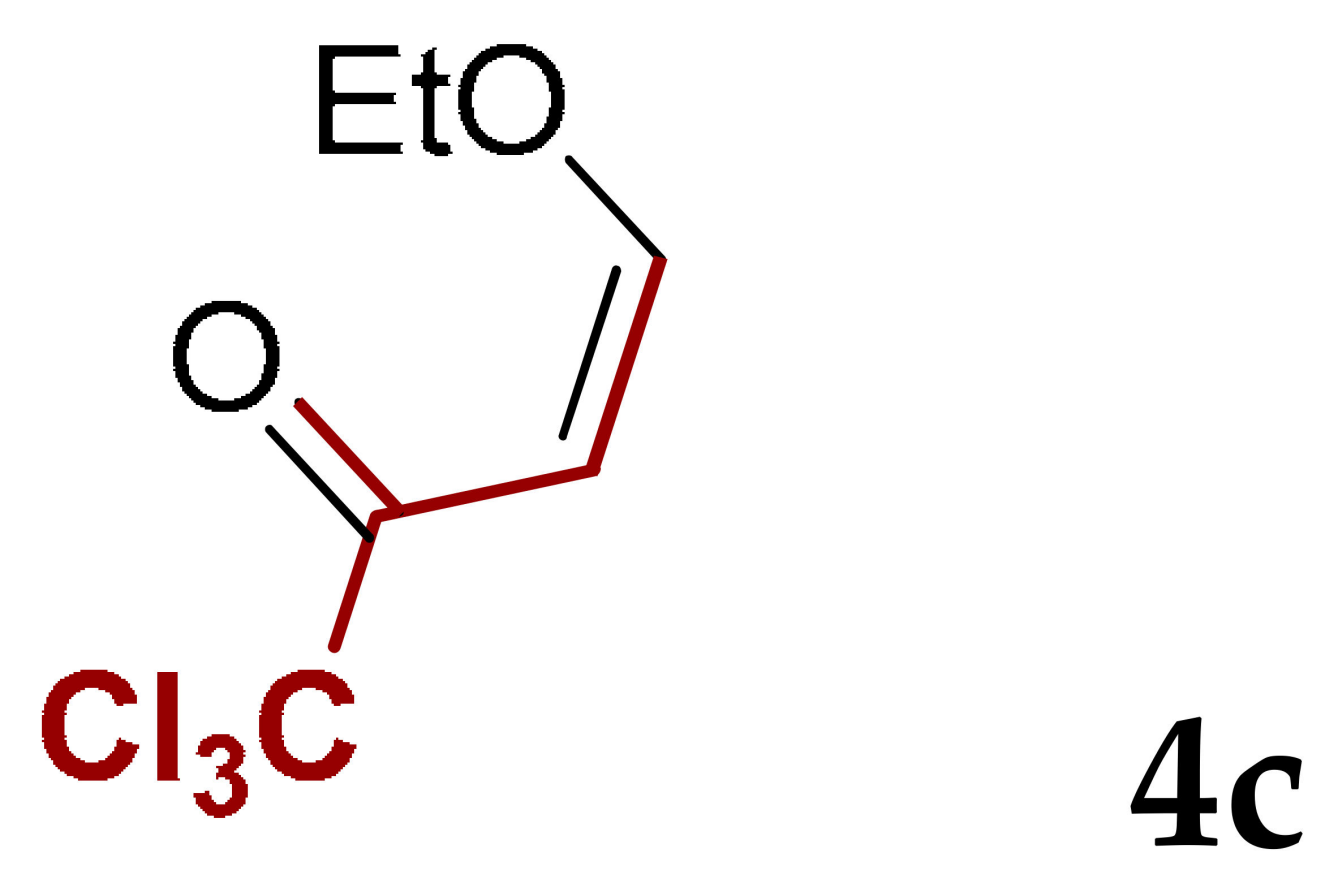	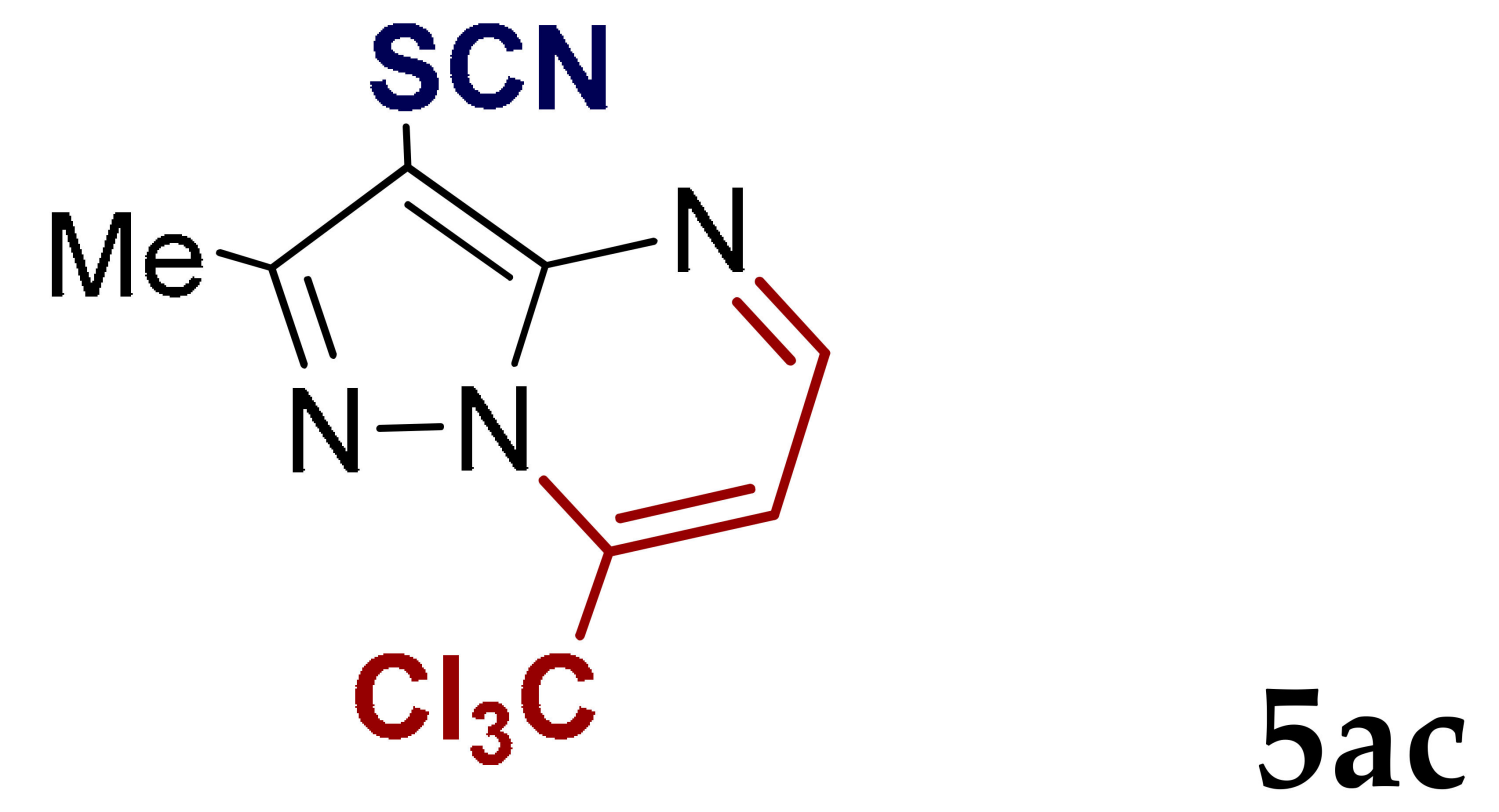	89
6 ^2^	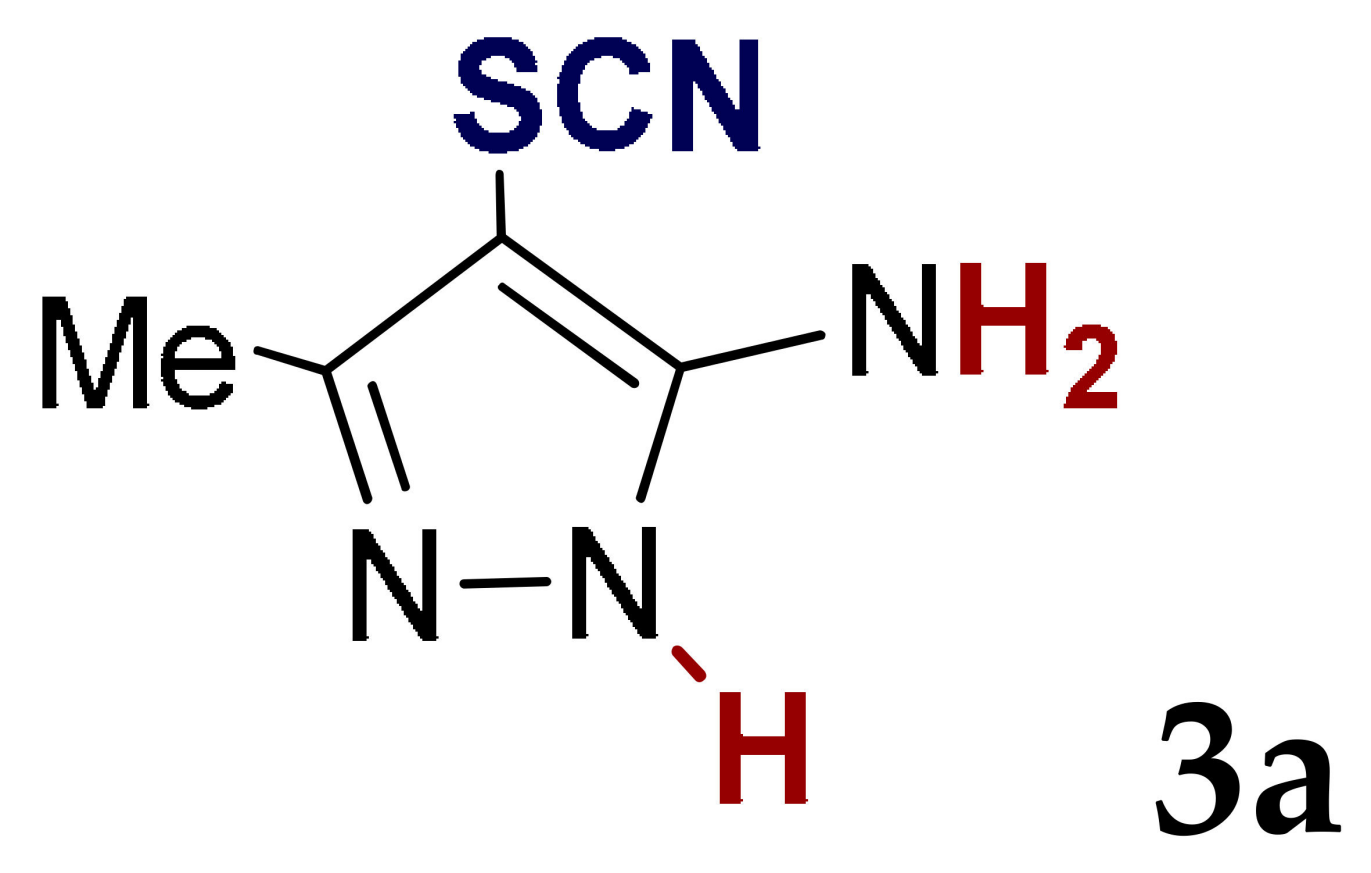	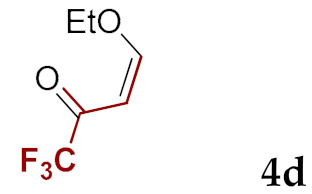	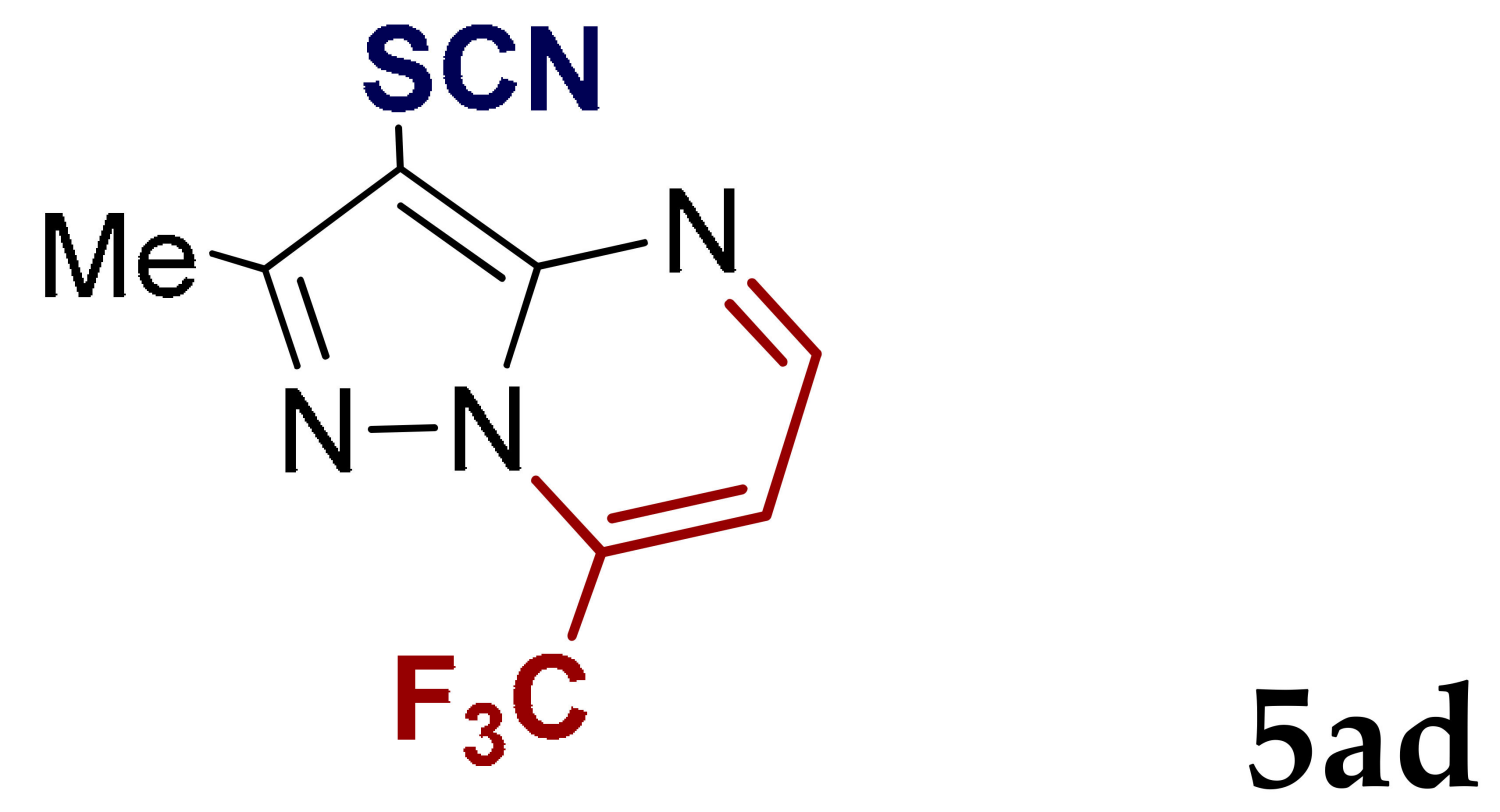	78
7 ^2^	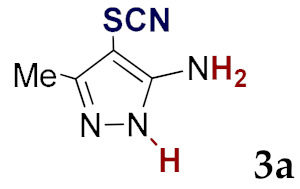	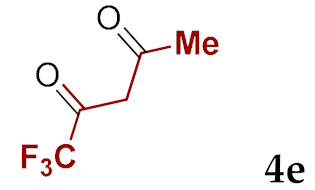	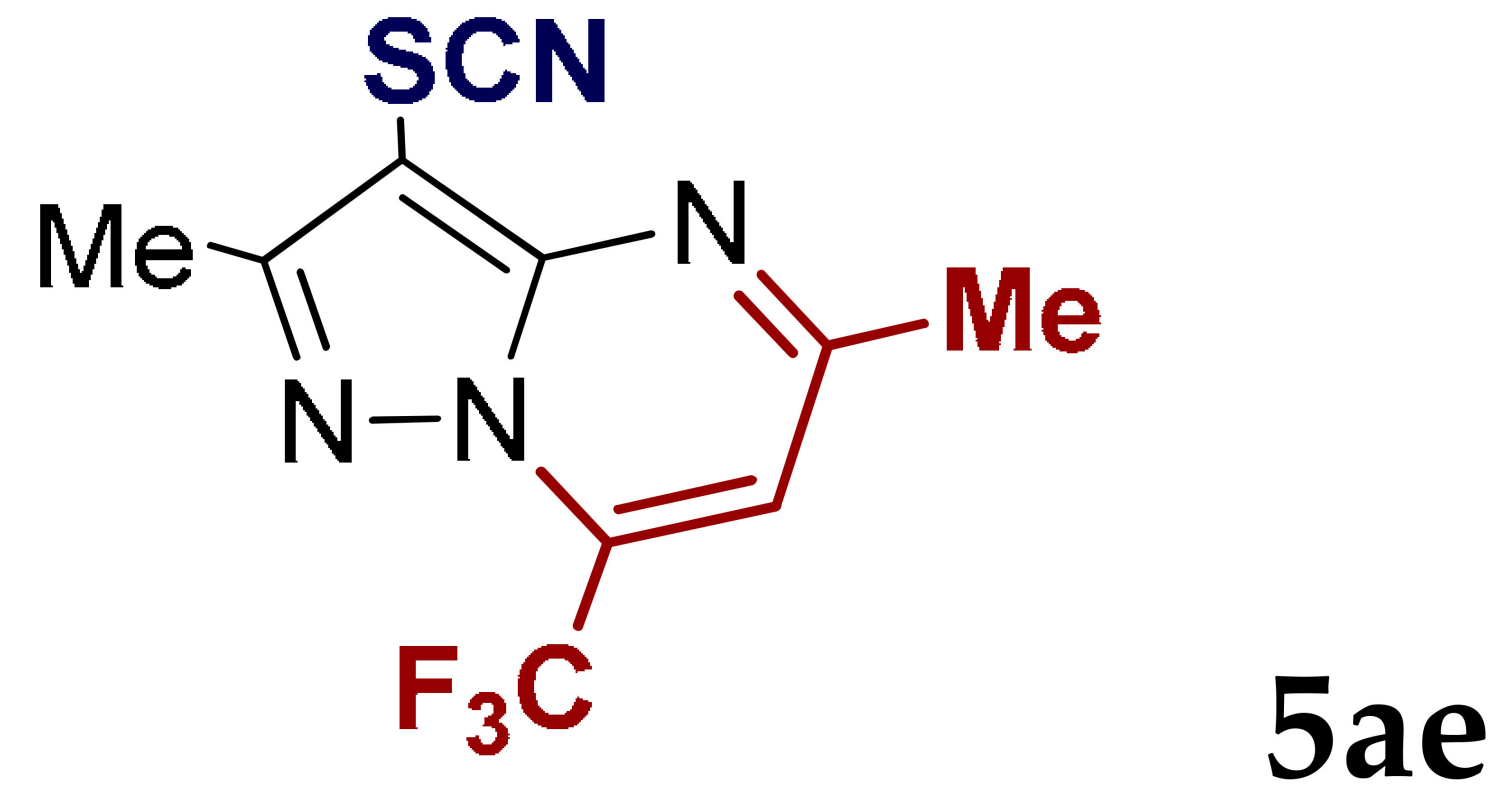	71
8 ^2^	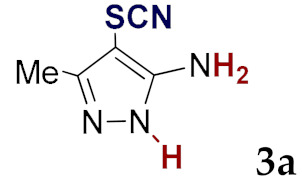	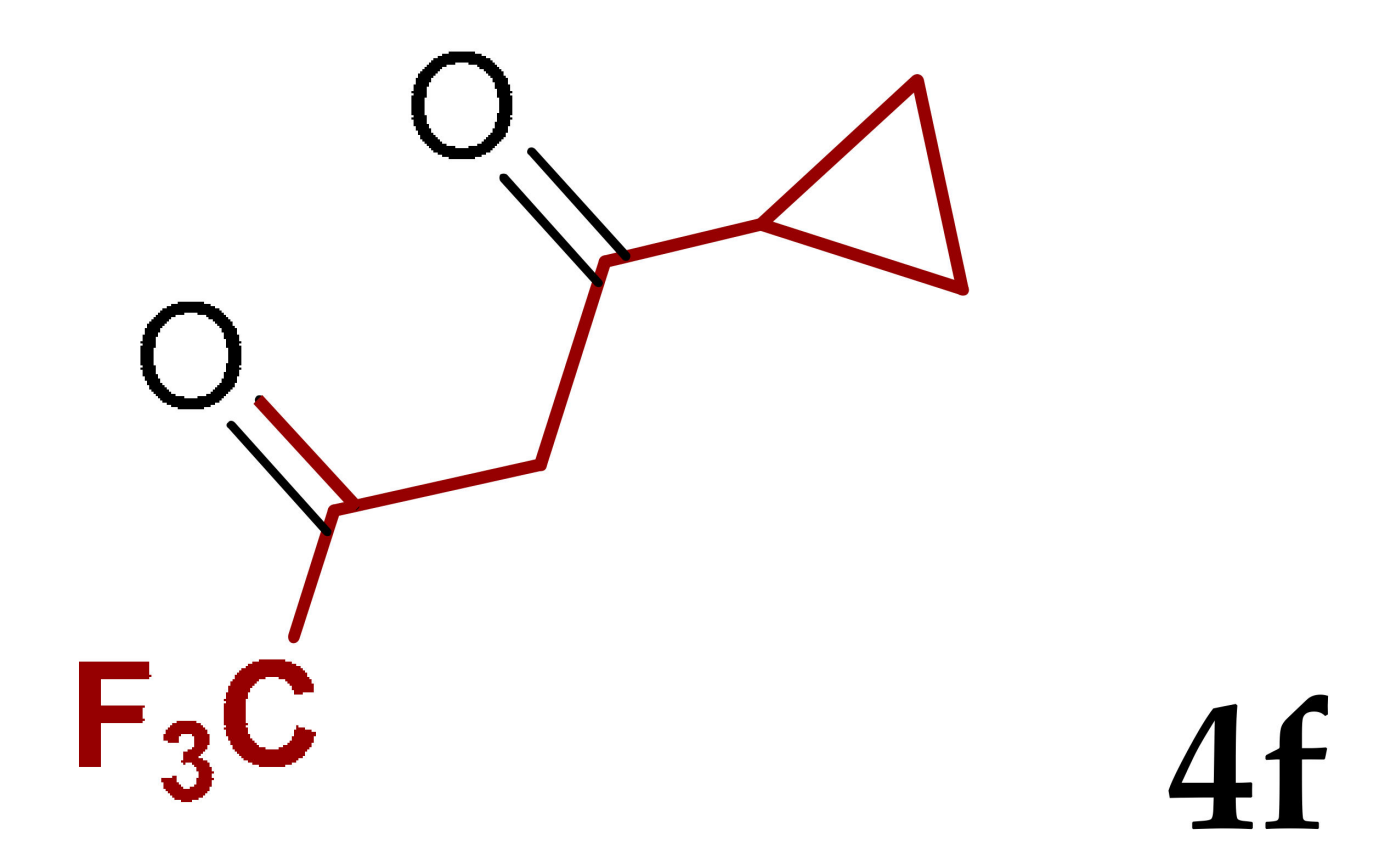	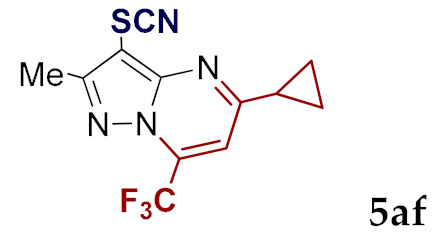	87
9 ^3^	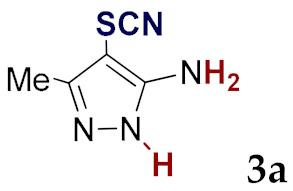	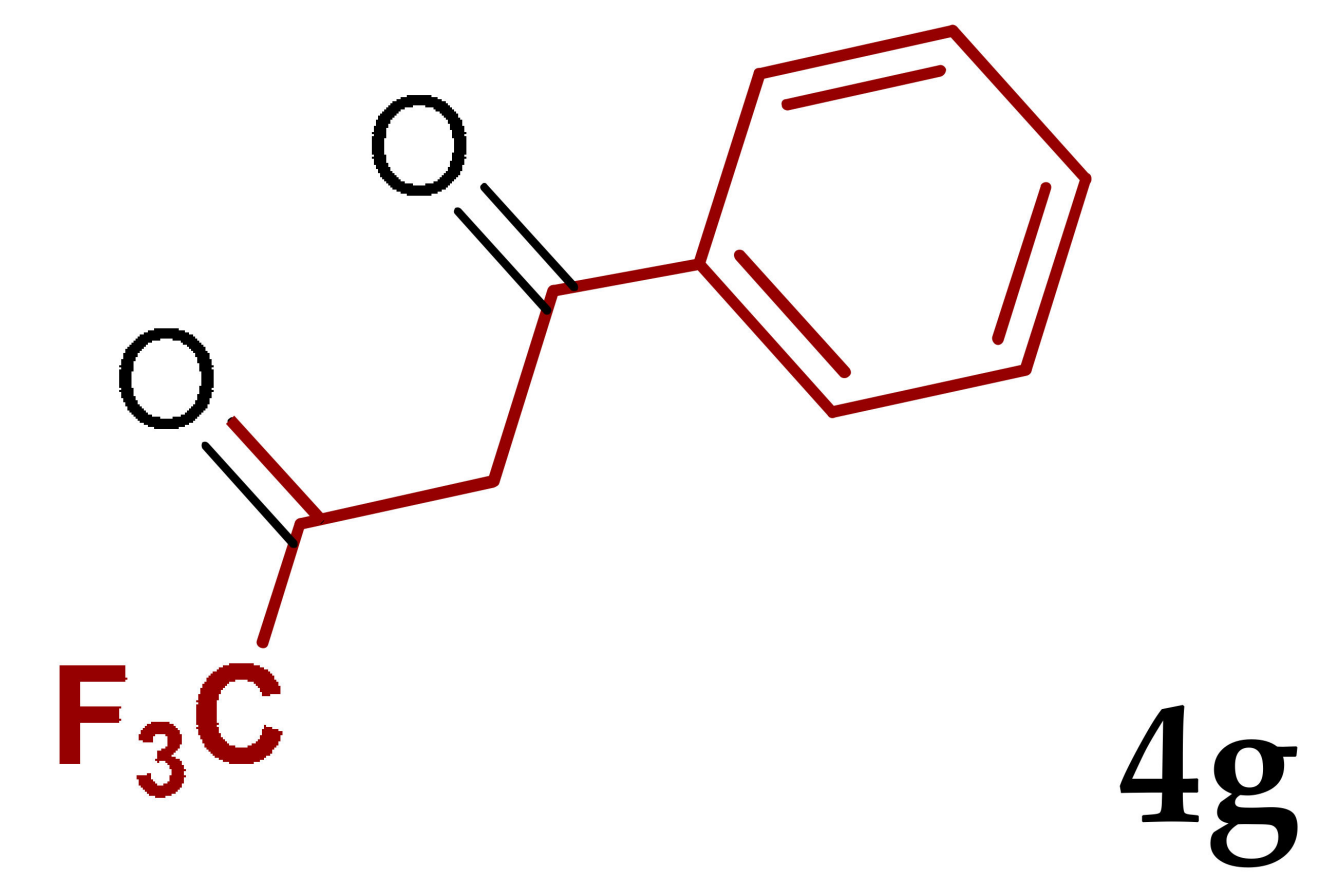	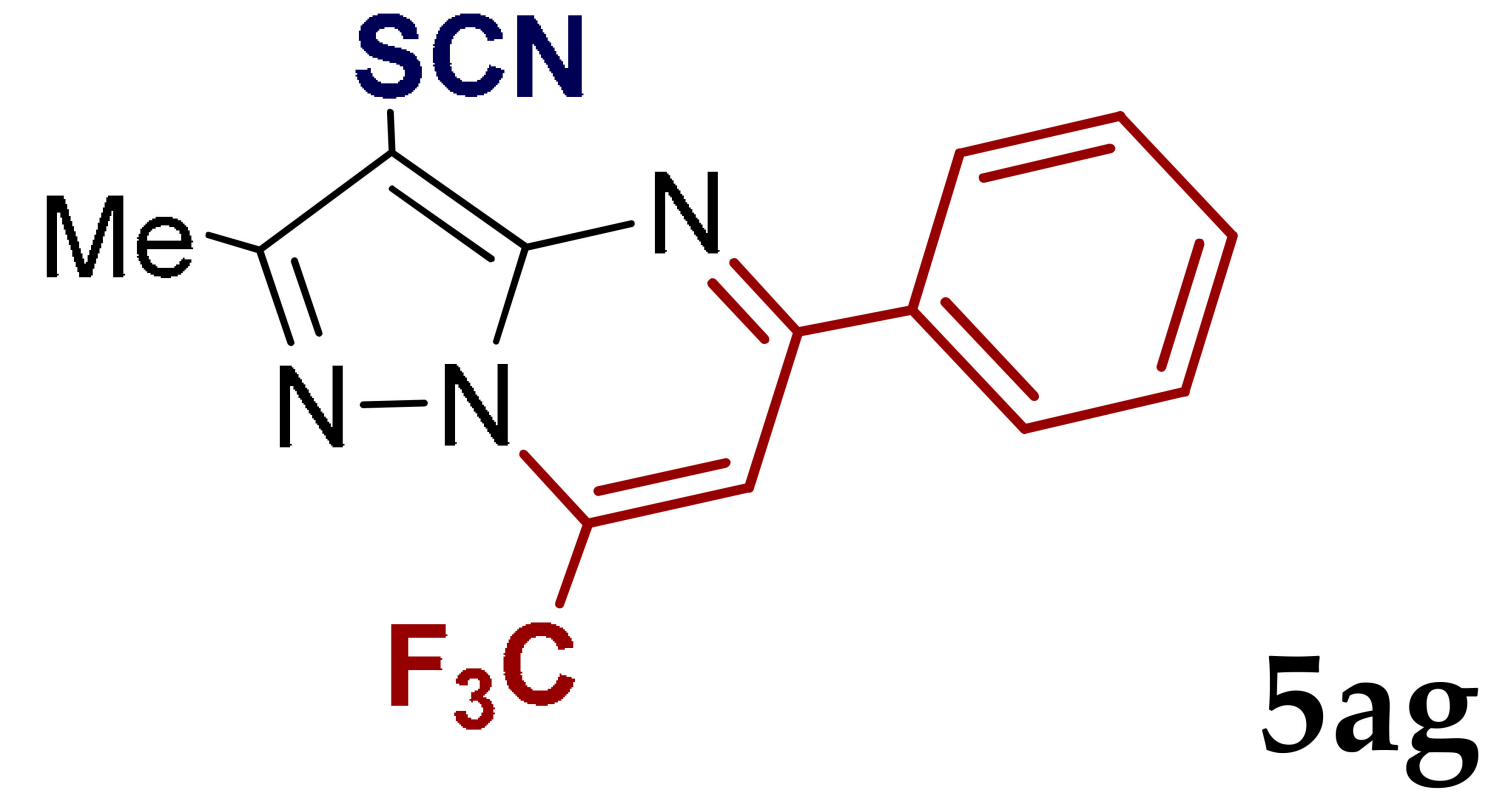	84
10 ^3^	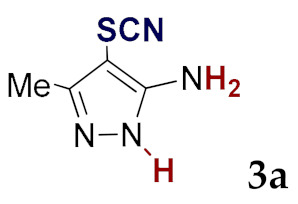	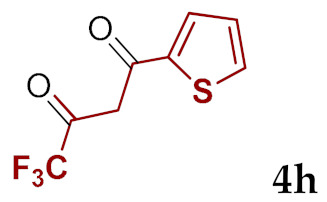	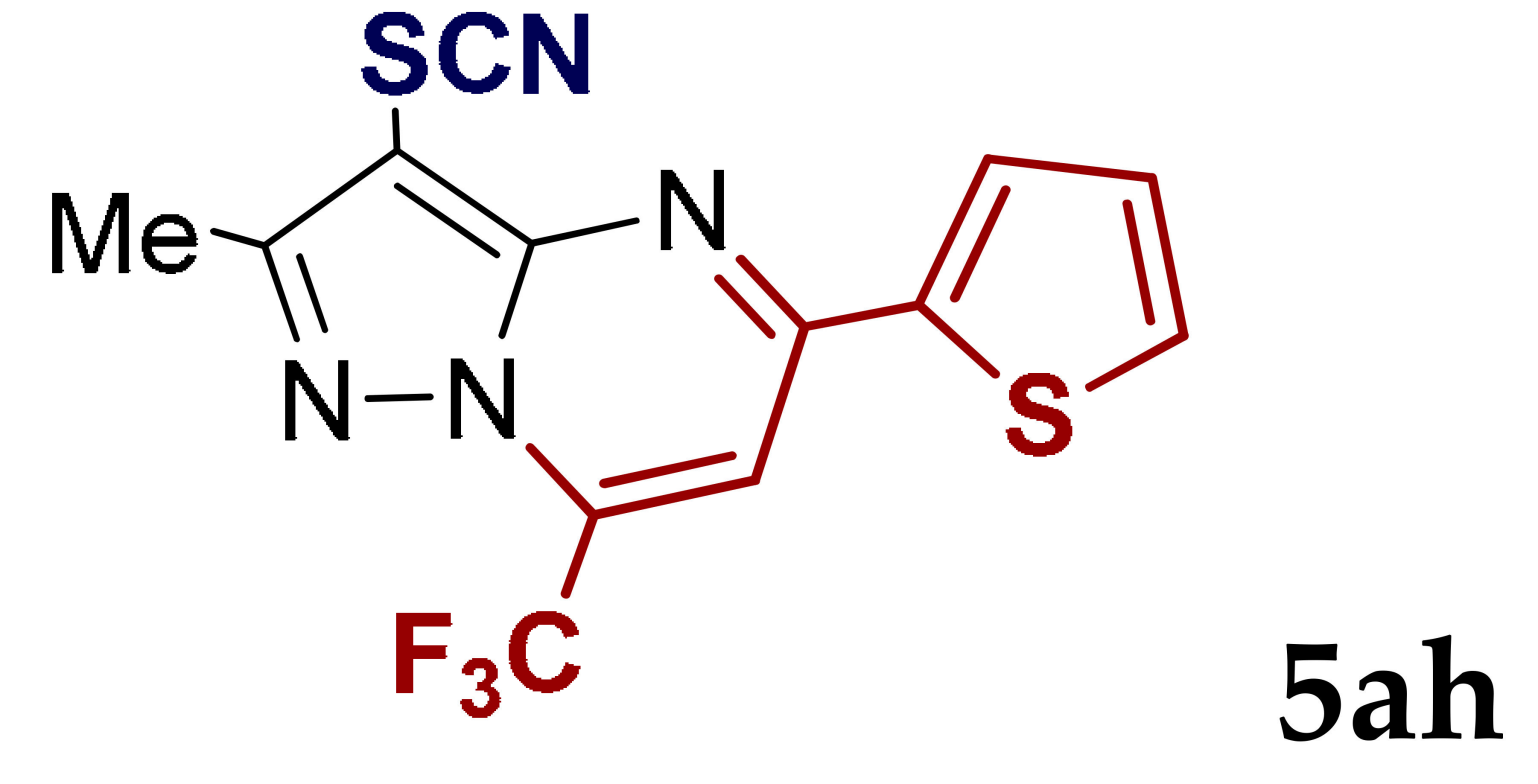	91

^1^ Azole **3a,b** (5 mmol) was dissolved in 15 mL H_2_O, then 2.5 mL of 32% aq. HCl and 1,3-dicarbonyl compound (or its derivative) **4a**–**h** (6 mmol) were added. The mixture was stirred for 24 h. The yield was determined for the isolated target product; ^2^ H_2_O-EtOH (1:4) instead of H_2_O; ^3^ EtOH instead of H_2_O.

## Supplementary Materials

The following are available online at https://www.mdpi.com/1420-3049/25/18/4169/s1, Figures S2, S5, S8, S11, S14, S17, S20, S23, S26, S29, S32, S35: ^1^H-NMR spectra; Figures S3, S6, S9, S12, S15, S18, S21, S24, S27, S30, S33, S36: ^13^C-NMR spectra; Figures S4, S7, S10, S13, S16, S19, S22, S25, S28, S31, S34, S37: HRMS data.

## Sample Availability

Samples of the compounds **3a,b**, **5aa**–**h**, **5ba**–**b** are available from the authors.
