# Supplementary material for: A New Method for the Synthesis of 3-Thiocyanatopyrazolo[1,5-a]pyrimidines
Source: Molecules. 2020 Sep 11;25(18):4169. doi: 10.3390/molecules25184169 (PMC7570695; doi:10.3390/molecules25184169)

| **Table of contents** | | | | | | | |
|---|---|---|---|---|---|---|---|
| NMR and HRM spectra for compounds **3a,b**; **5aa – 5ah** | | | | | | | |
| Compound | $^{1}$H NMR | $^{13}$C NMR | HRMS | Compound | $^{1}$H NMR | $^{13}$C NMR | HRMS |
| **3a** | S2 | S3 | S4 | **5ac** | S20 | S21 | S22 |
| **3b** | S5 | S6 | S7 | **5ad** | S23 | S24 | S25 |
| **5aa** | S8 | S9 | S10 | **5ae** | S26 | S27 | S28 |
| **5ba** | S11 | S12 | S13 | **5af** | S29 | S30 | S31 |
| **5ab** | S14 | S15 | S16 | **5ag** | S32 | S33 | S34 |
| **5bb** | S17 | S18 | S19 | **5ah** | S35 | S36 | S37 |

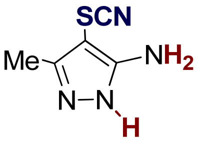


**3a**

**$^1$H NMR**
**600.1 MHz**
**DMSO-$d_6$**

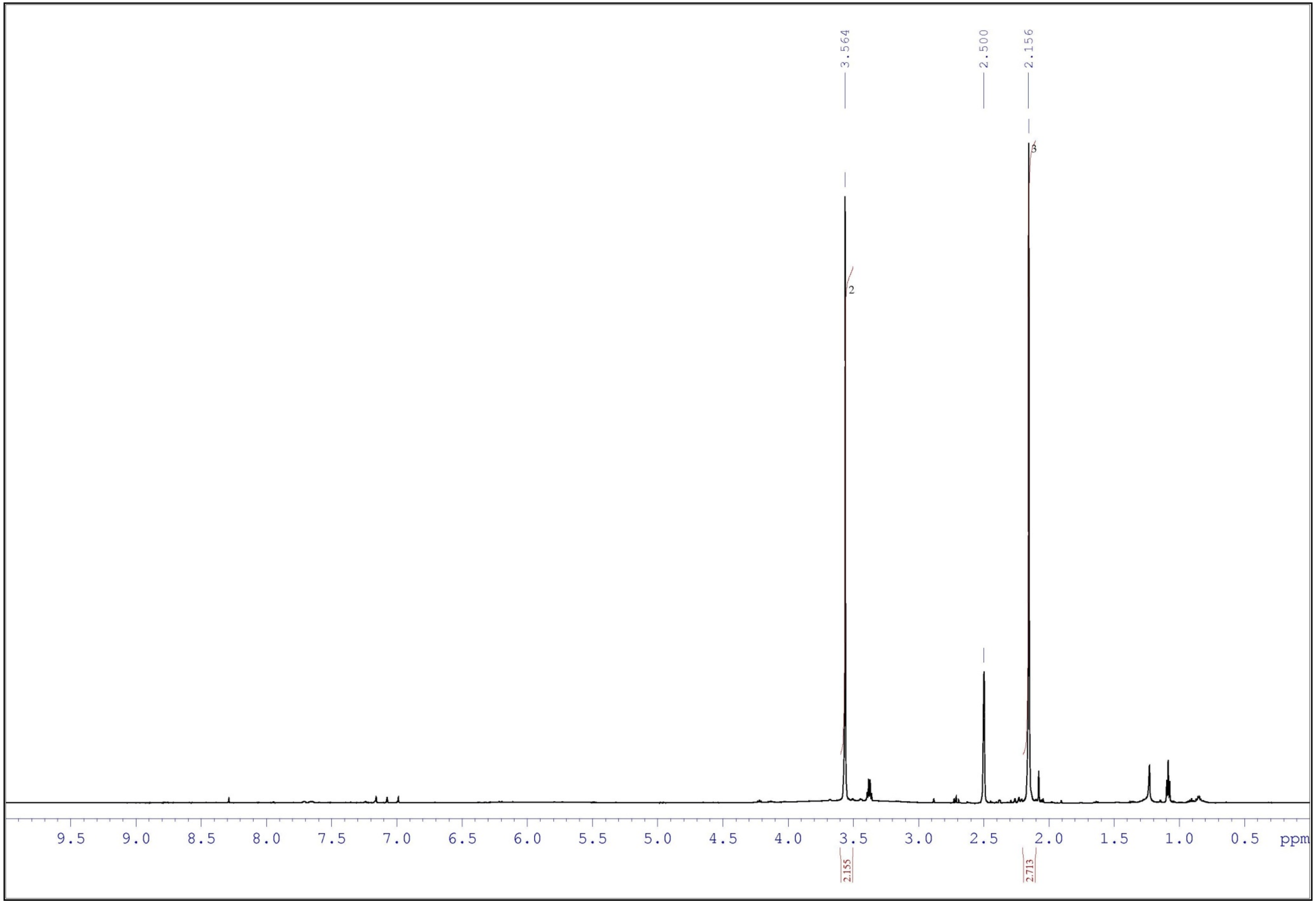

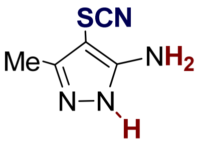


**3a**

**$^{13}C$ NMR**
**125.8 MHz**
**DMSO-$d_6$**

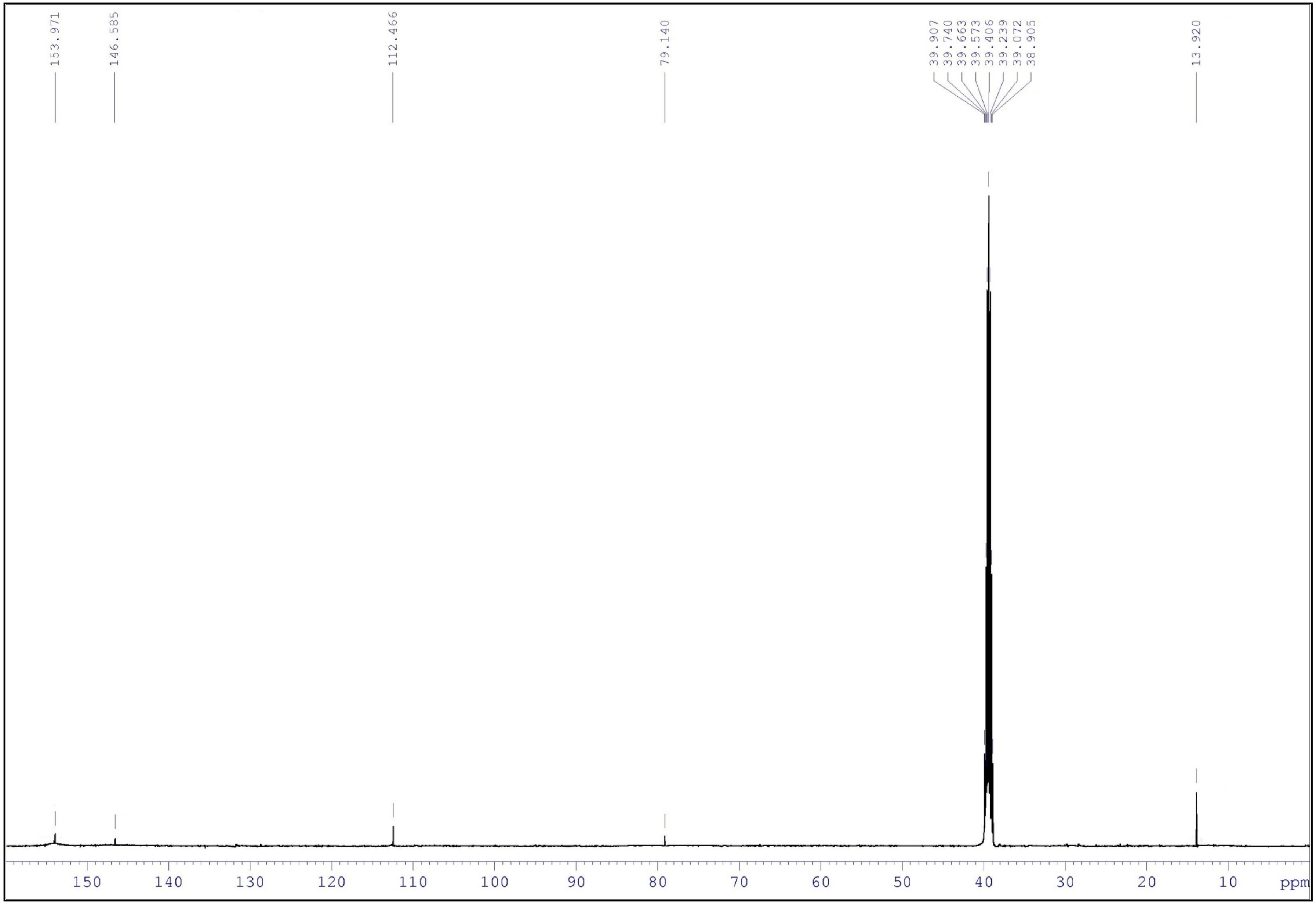

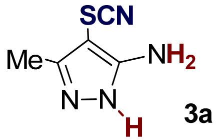


**3a**

**HRMS (ESI)**

**Chemical Formula: $C_5H_6N_4S$**

**Exact Mass: 154,03**

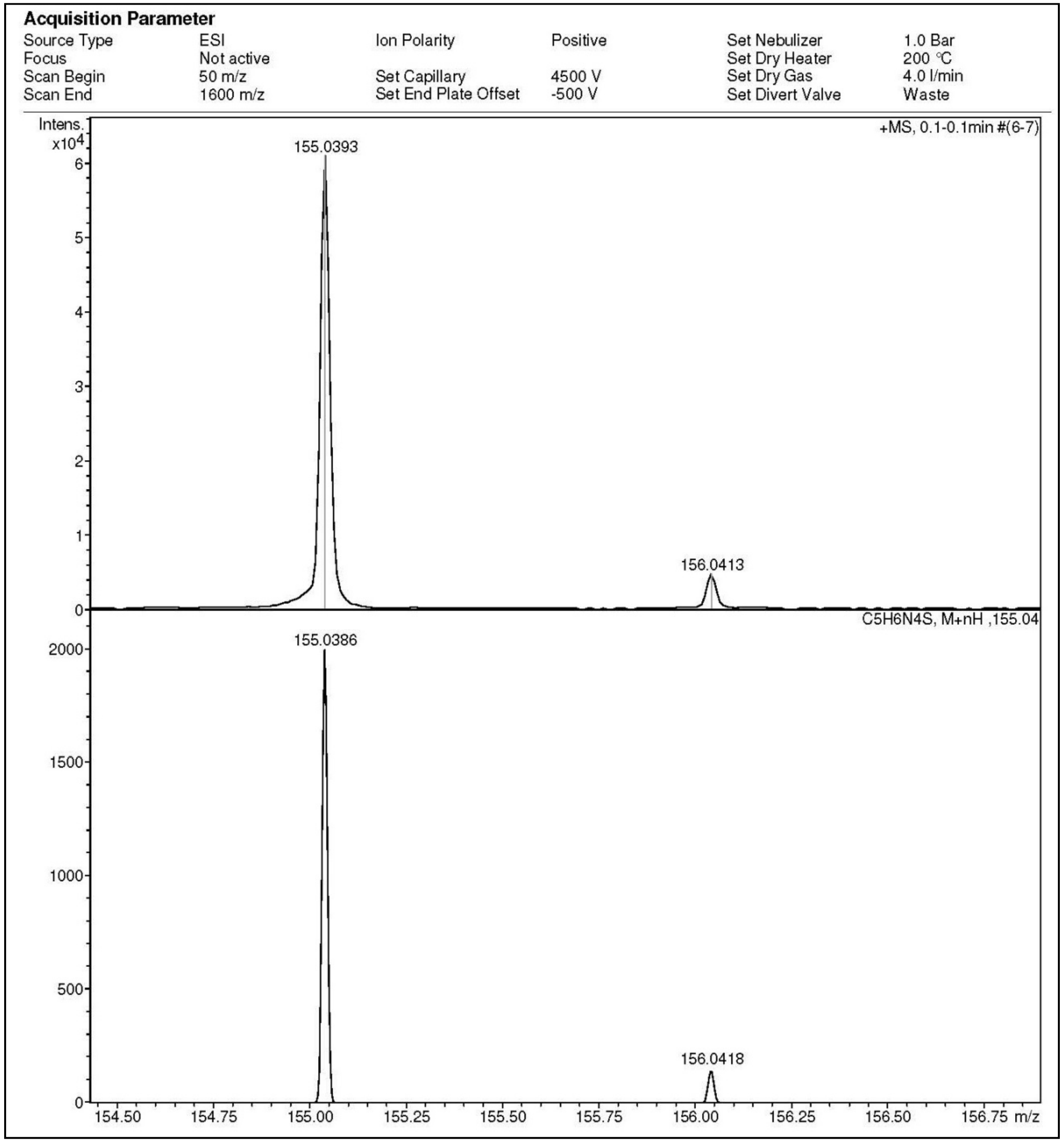

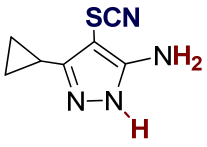


**3b**

**$^{1}$H NMR**

**300.1 MHz**

**$CDCl_3$**

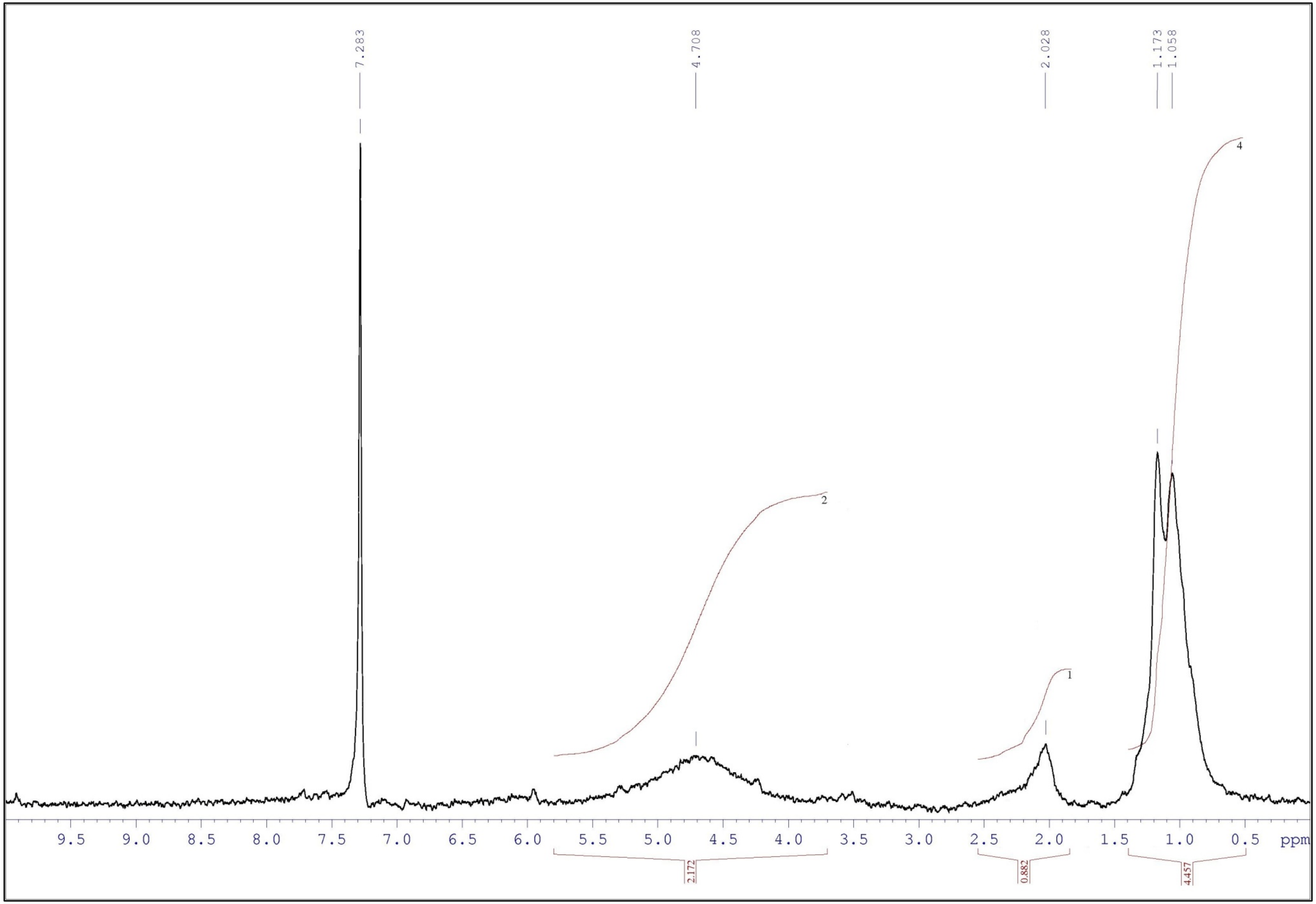

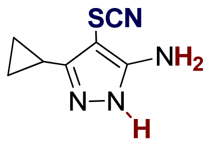


**3b**

**$^{13}$C NMR**
**150.9 MHz**
**$CDCl_3$**

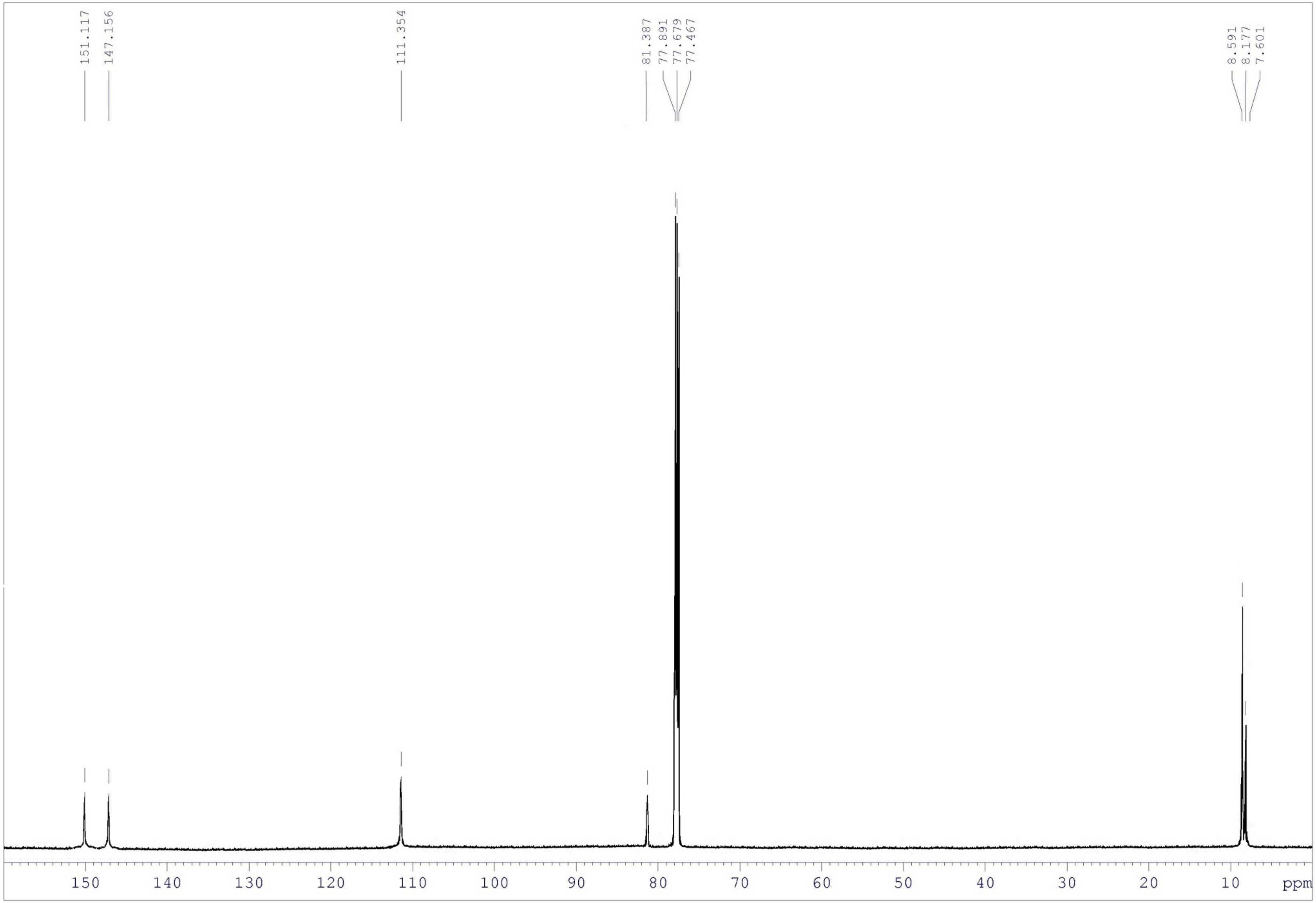

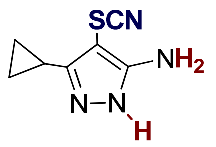


**3b**

**HRMS (ESI)**

**Chemical Formula: $C_7H_8N_4S$**

**Exact Mass: 180,05**

**Acquisition Parameter**

| | | | | | |
|---|---|---|---|---|---|
| Source Type | ESI | Ion Polarity | Positive | Set Nebulizer | 0.4 Bar |
| Focus | Not active | | | Set Dry Heater | 180 °C |
| Scan Begin | 50 m/z | Set Capillary | 4500 V | Set Dry Gas | 4.0 l/min |
| Scan End | 3000 m/z | Set End Plate Offset | -500 V | Set Divert Valve | Waste |

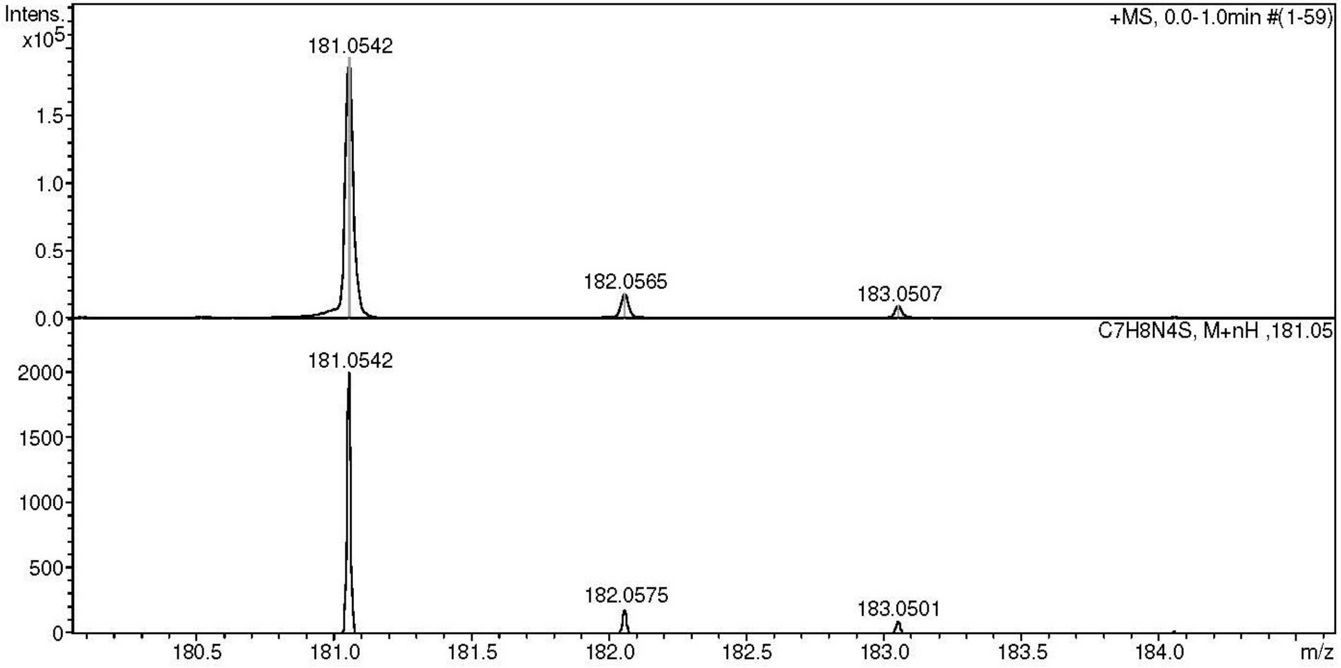

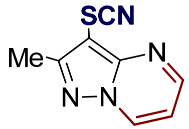


**5aa**

**$^{1}$H NMR**

**300.1 MHz**

**$CDCl_3$**

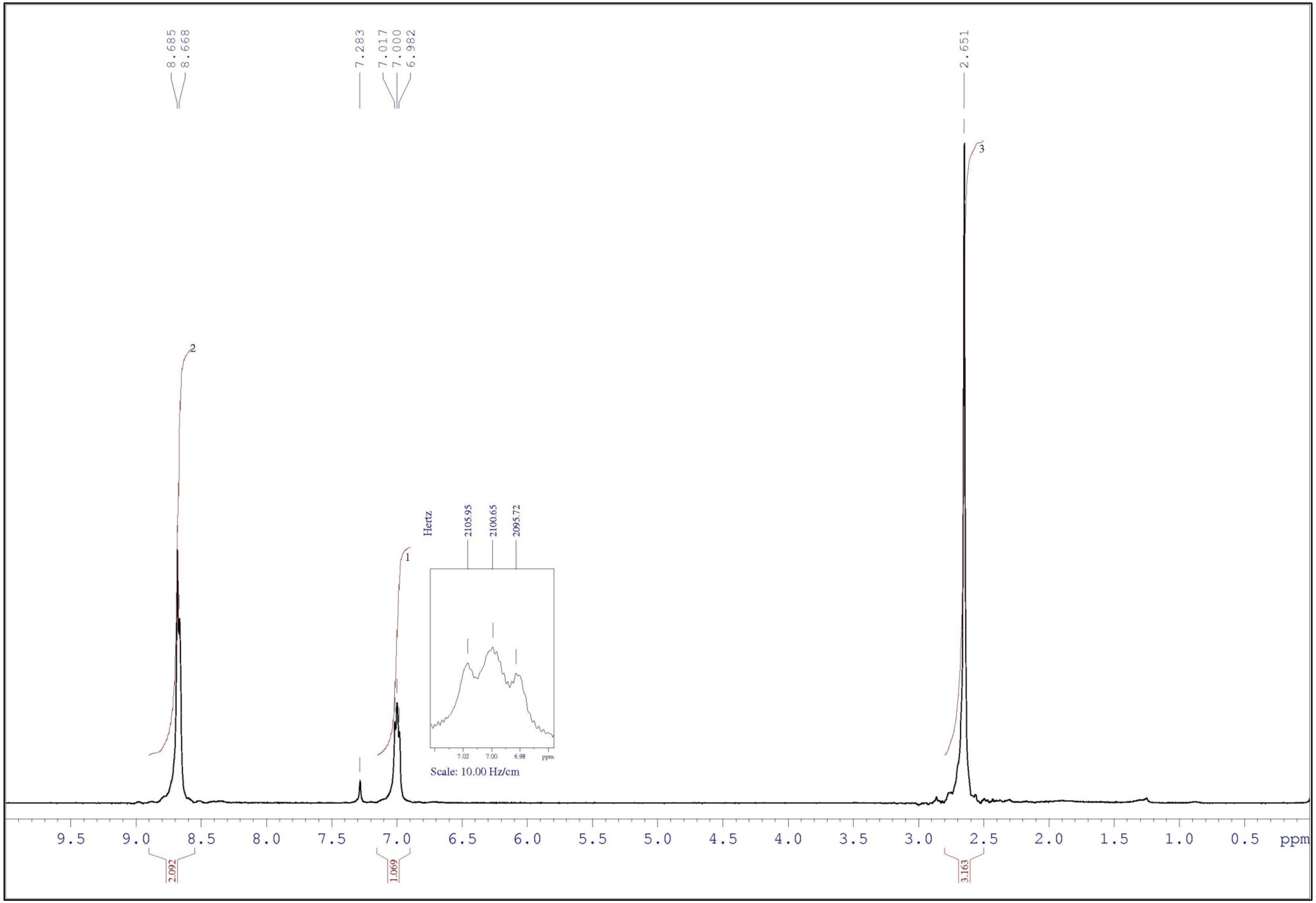

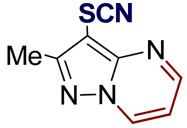

**5aa**

**$^{13}$C NMR**
**75.5 MHz**
**$CDCl_3$**

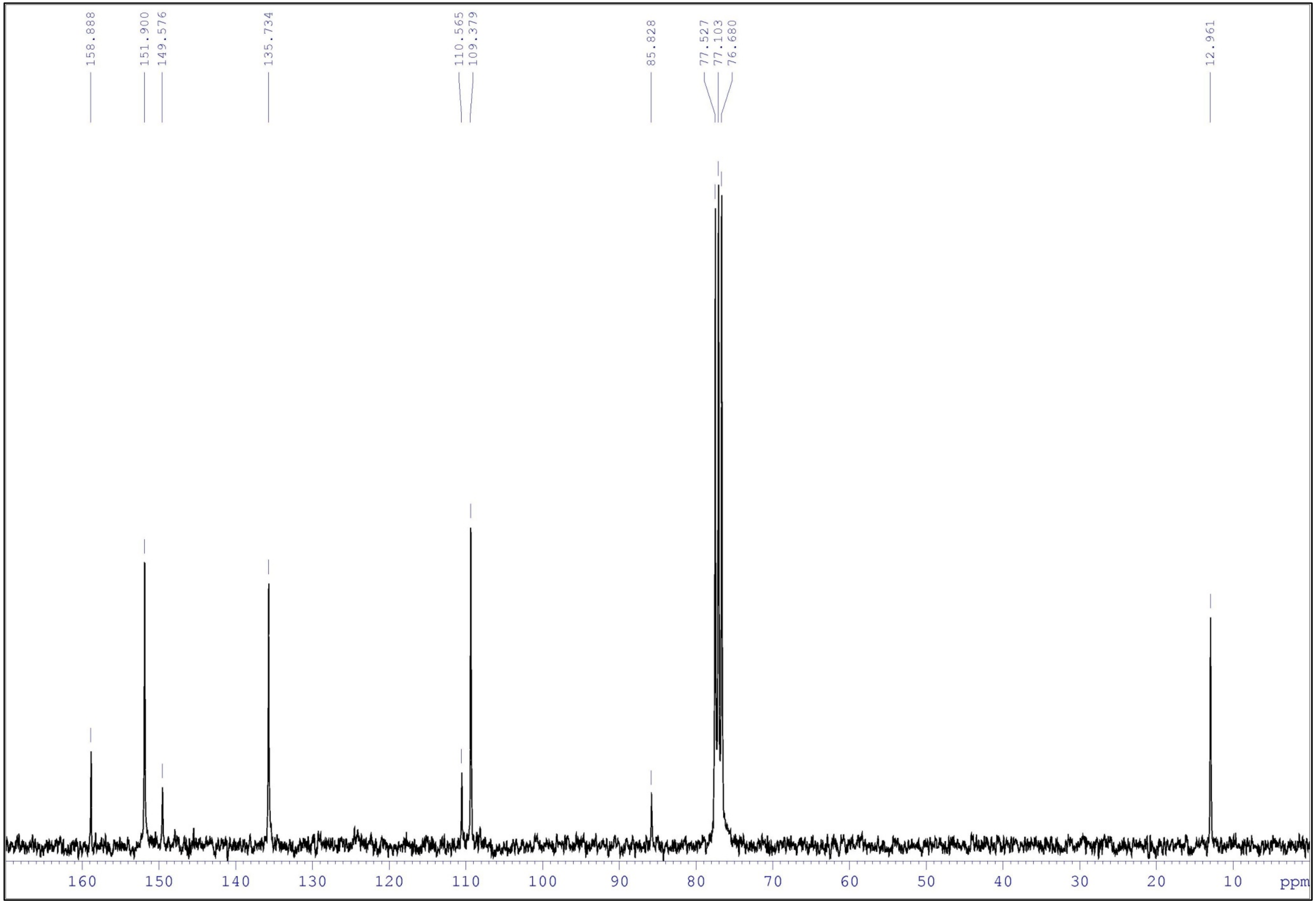

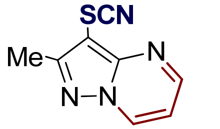


**5aa**

**HRMS (ESI)**

**Chemical Formula: $C_8H_6N_4S$**

**Exact Mass: 190,03**

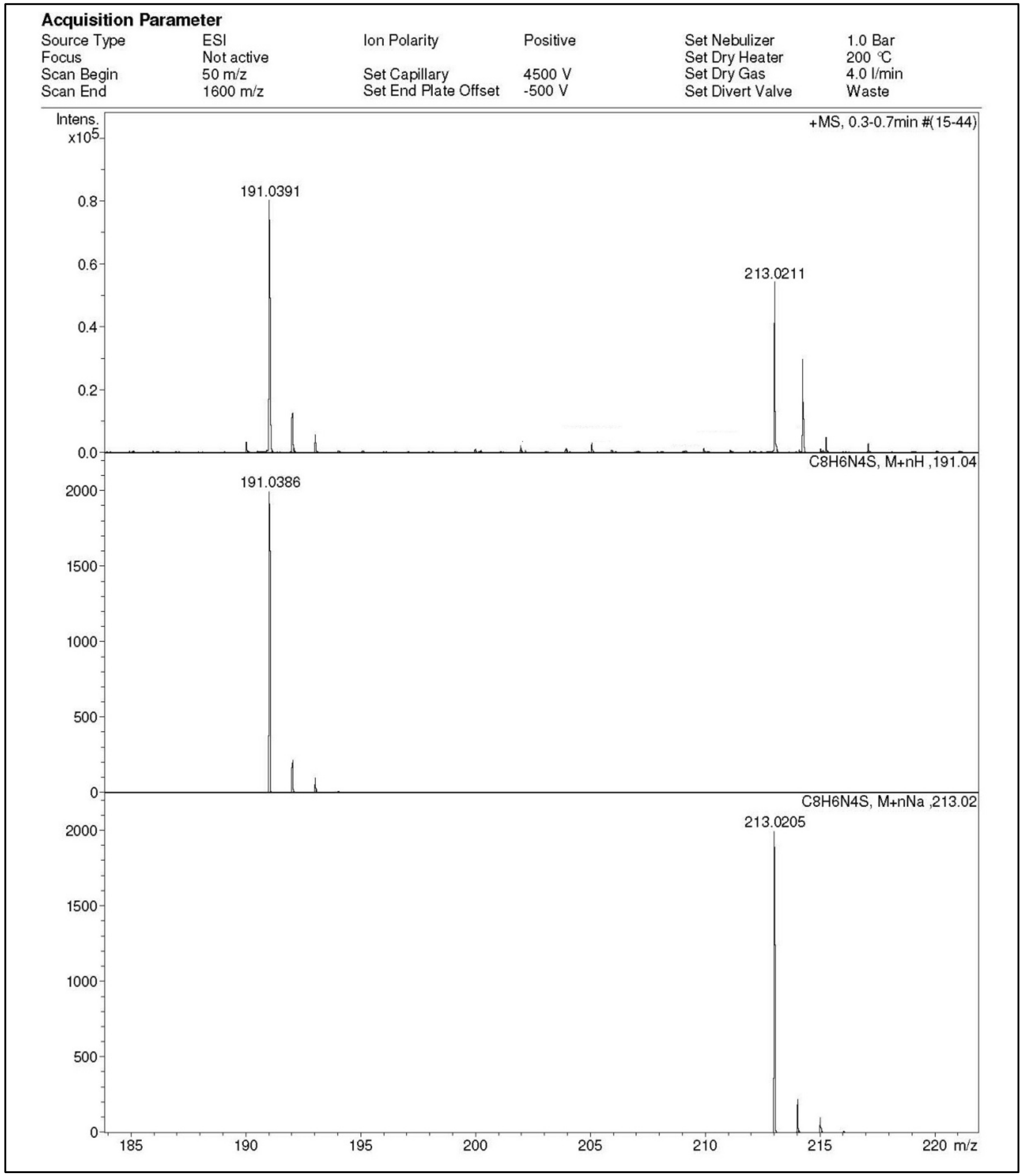

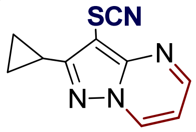

5ba

$^{1}$H NMR
300.1 MHz
$CDCl_3$

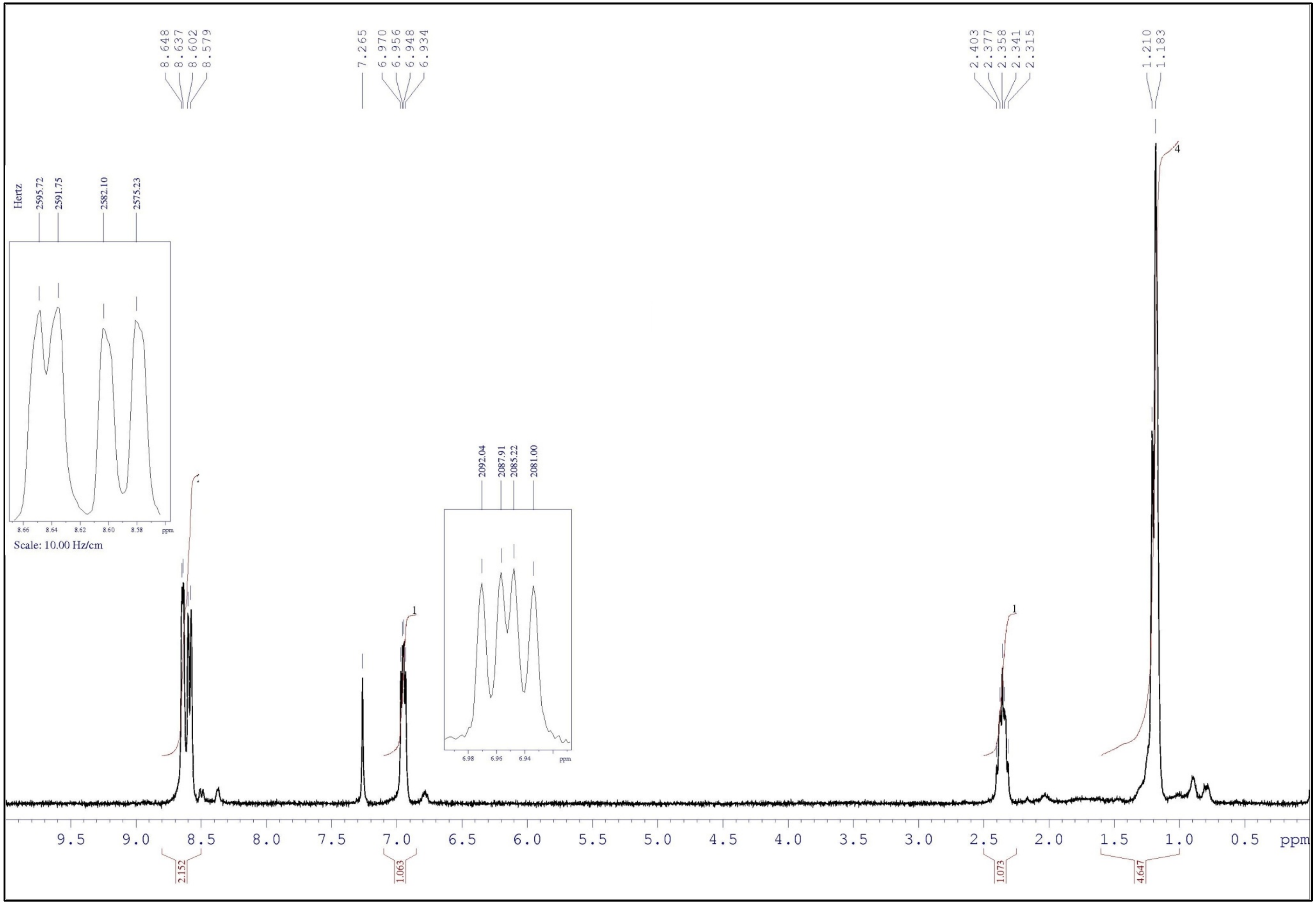

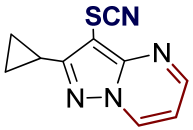

**5ba**

**$^{13}$C NMR**
**150.9 MHz**
**$CDCl_3$**

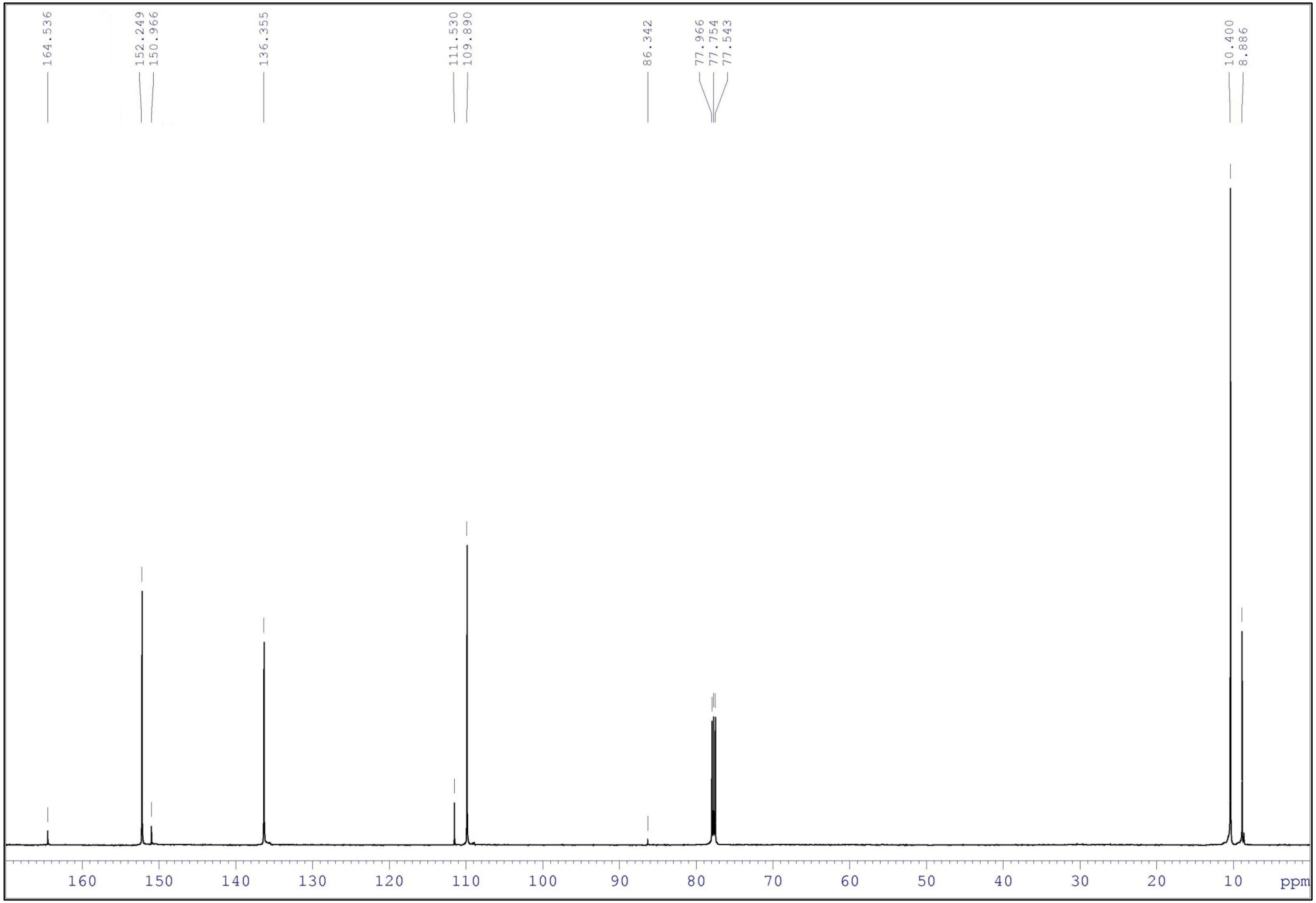

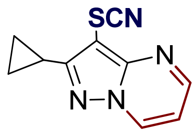


**5ba**

**HRMS (ESI)**

**Chemical Formula: $C_{10}H_8N_4S$**

**Exact Mass: 216,05**

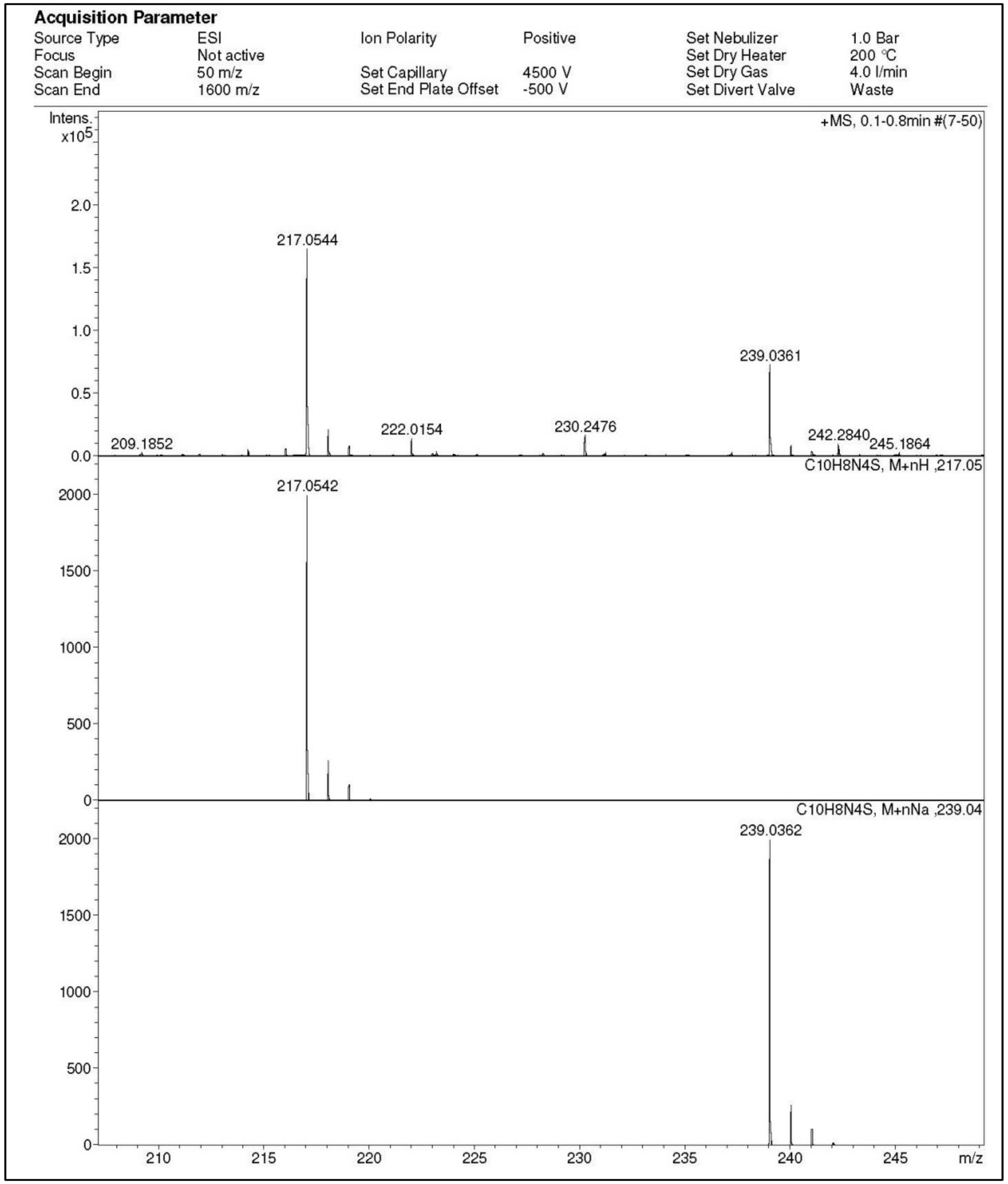

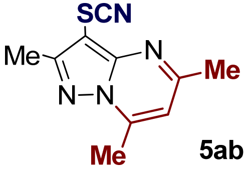


5ab

$^{1}H$ NMR
300.1 MHz
$CDCl_3$

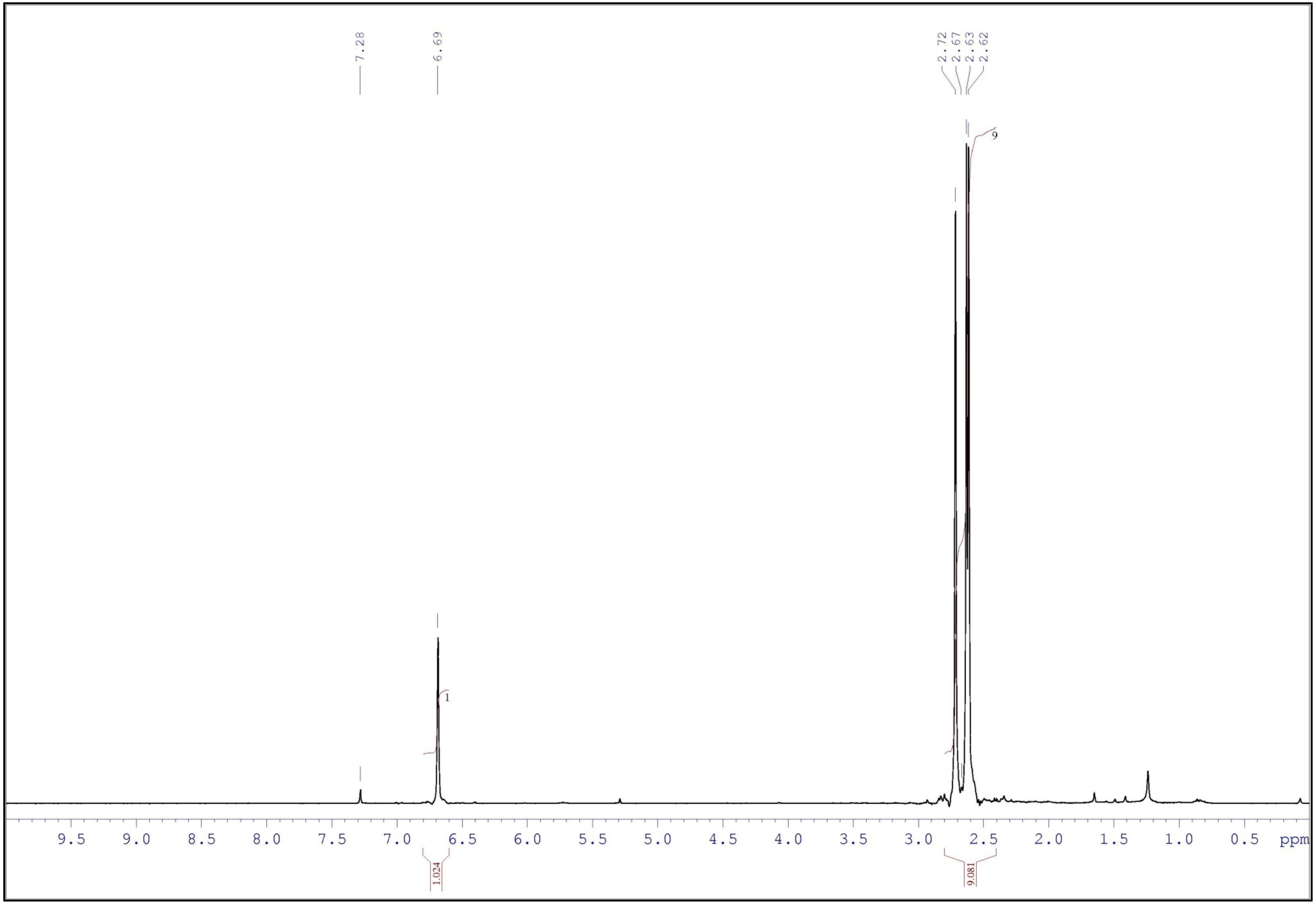

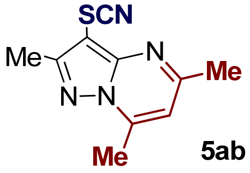

5ab

$^{13}$C NMR

75.5 MHz

$CDCl_3$

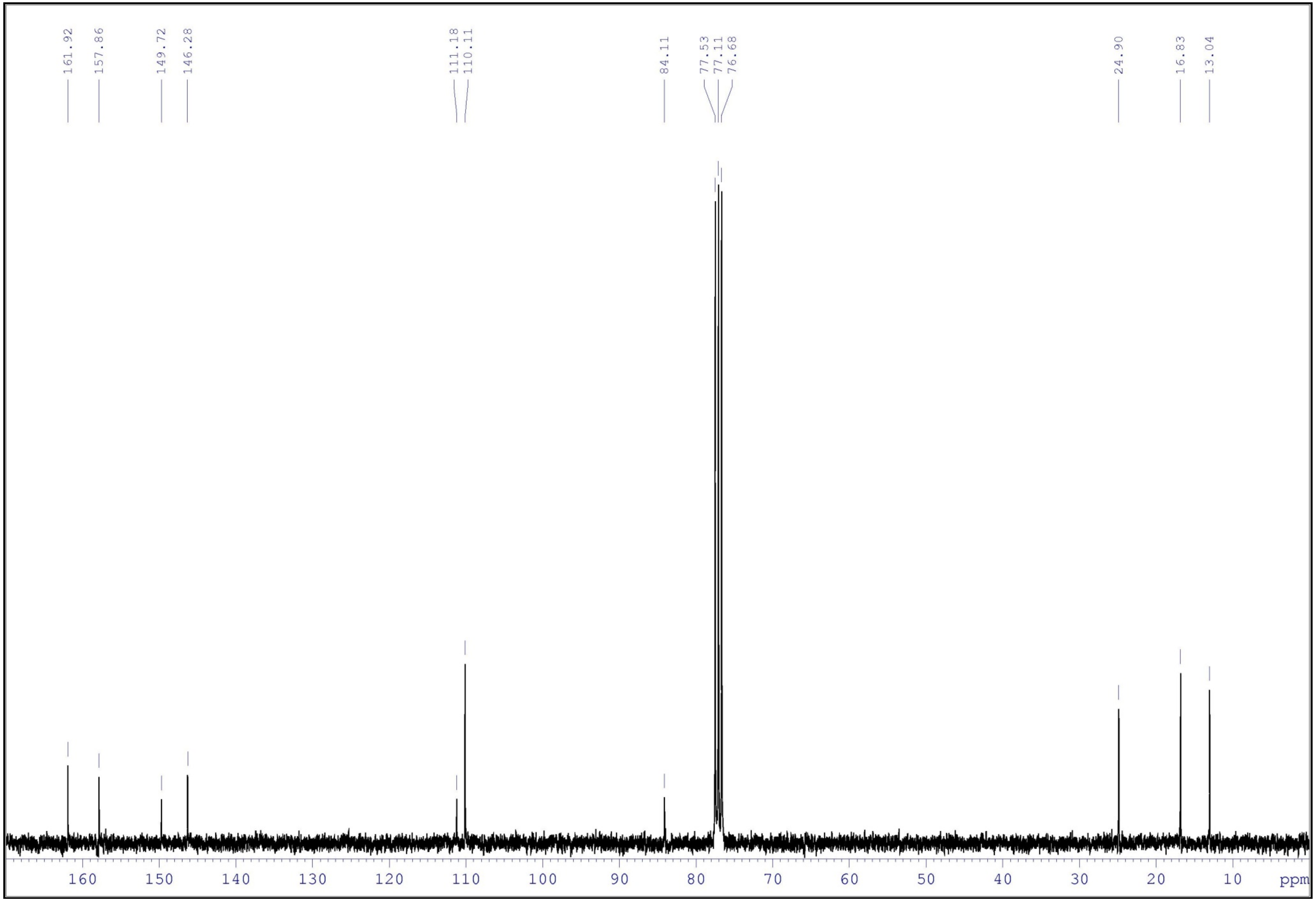

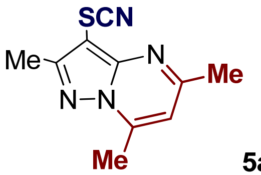


**5ab**

**HRMS (ESI)**

**Chemical Formula: $C_{10}H_{10}N_4S$**

**Exact Mass: 218,06**

**Acquisition Parameter**

| | | | | | |
|---|---|---|---|---|---|
| Source Type | ESI | Ion Polarity | Positive | Set Nebulizer | 0.4 Bar |
| Focus | Not active | | | Set Dry Heater | 180 °C |
| Scan Begin | 50 m/z | Set Capillary | 4500 V | Set Dry Gas | 4.0 l/min |
| Scan End | 3000 m/z | Set End Plate Offset | -500 V | Set Divert Valve | Waste |

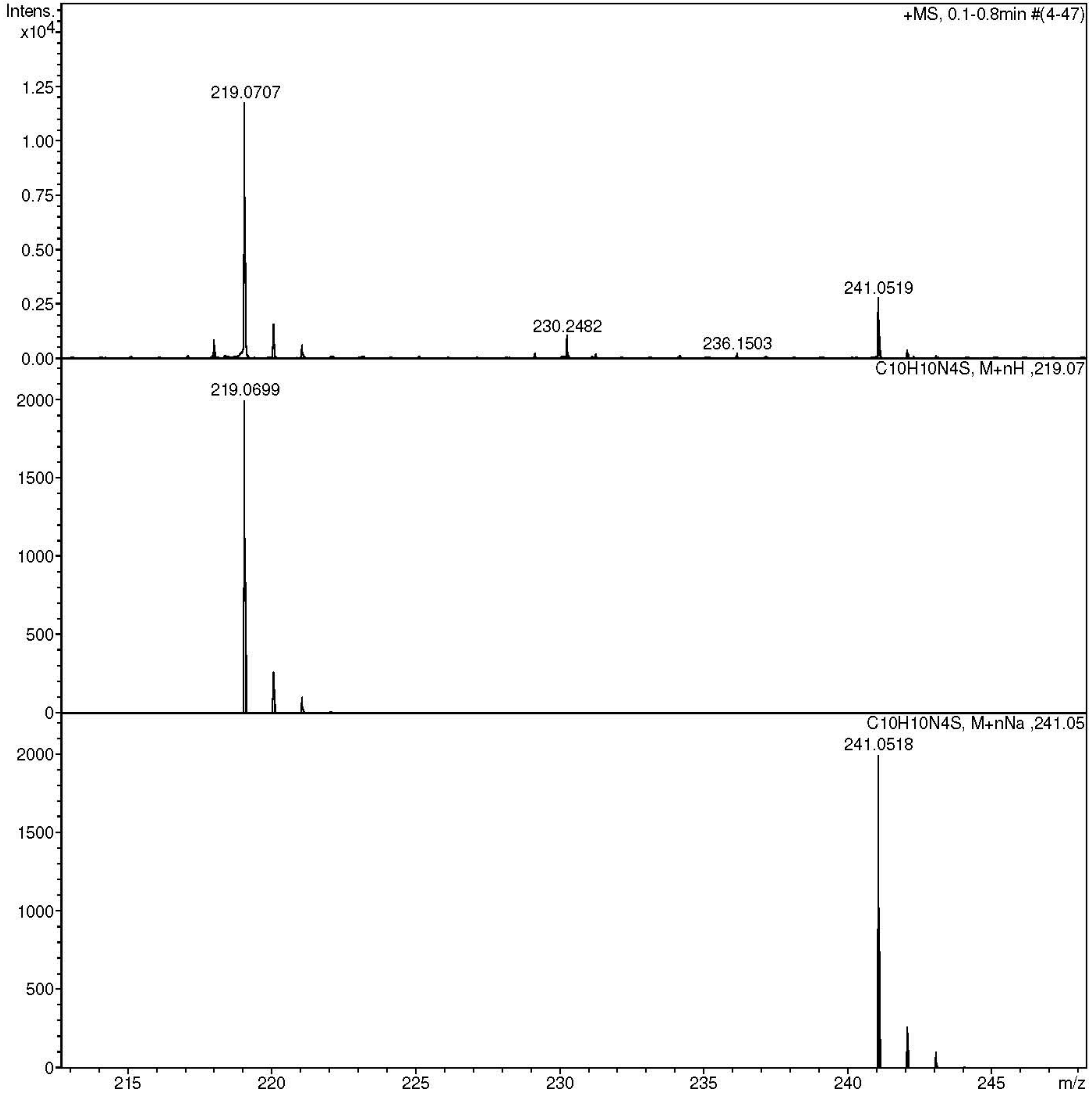

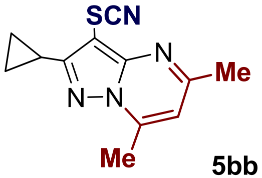


**5bb**

**$^1$H NMR**
**300.1 MHz**
**$CDCl_3$**

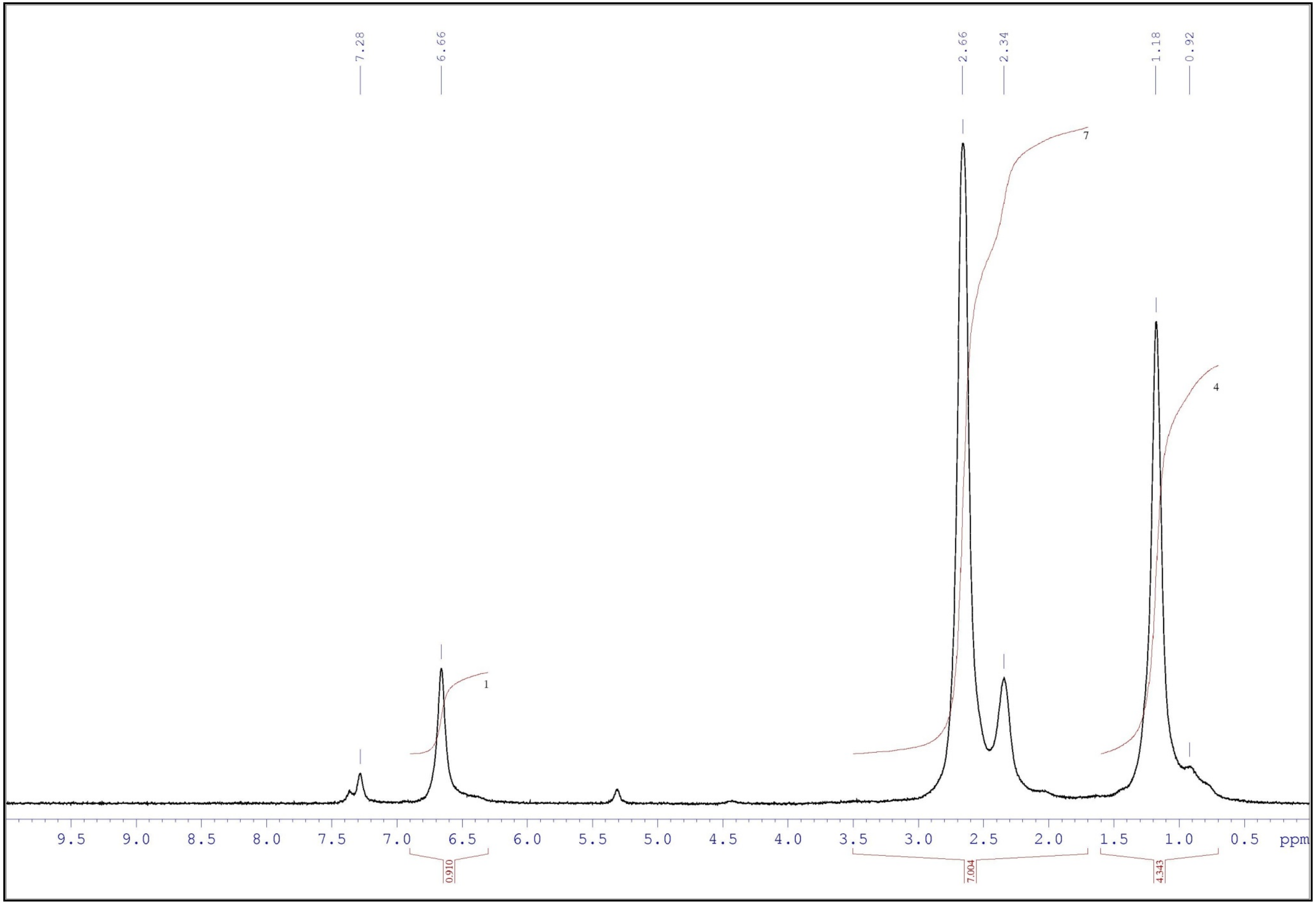

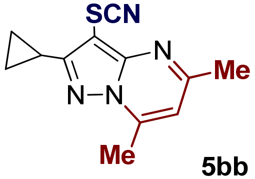


5bb

$^{13}$C NMR
75.5 MHz
$CDCl_3$

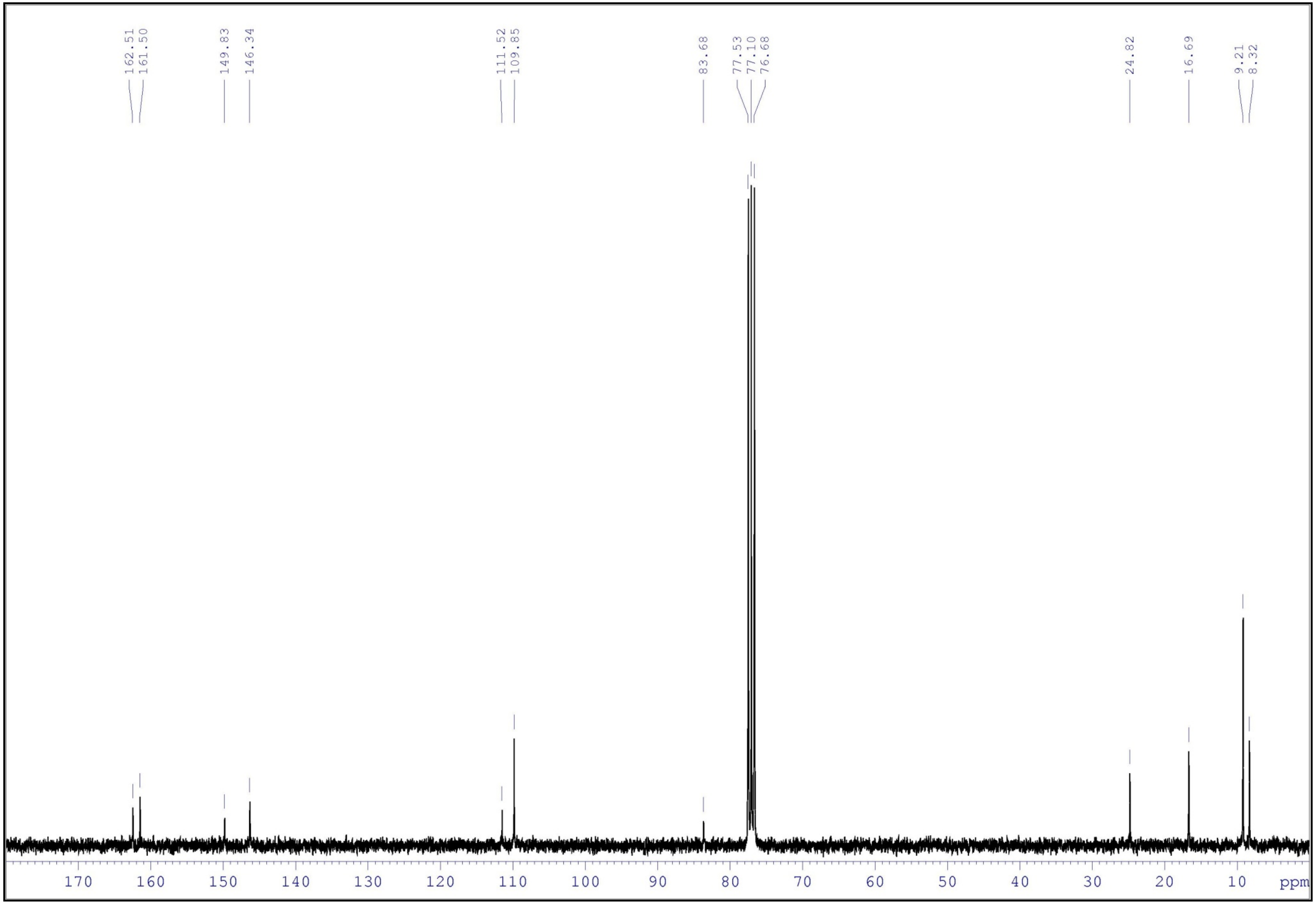

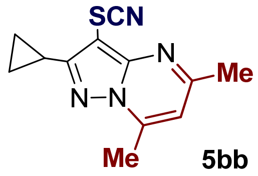


**5bb**

**HRMS (ESI)**

**Chemical Formula: $C_{12}H_{12}N_4S$**

**Exact Mass: 244,08**

**Acquisition Parameter**

| | | | | | |
|---|---|---|---|---|---|
| Source Type | ESI | Ion Polarity | Positive | Set Nebulizer | 0.4 Bar |
| Focus | Not active | | | Set Dry Heater | 180 °C |
| Scan Begin | 50 m/z | Set Capillary | 4500 V | Set Dry Gas | 4.0 l/min |
| Scan End | 3000 m/z | Set End Plate Offset | -500 V | Set Divert Valve | Waste |

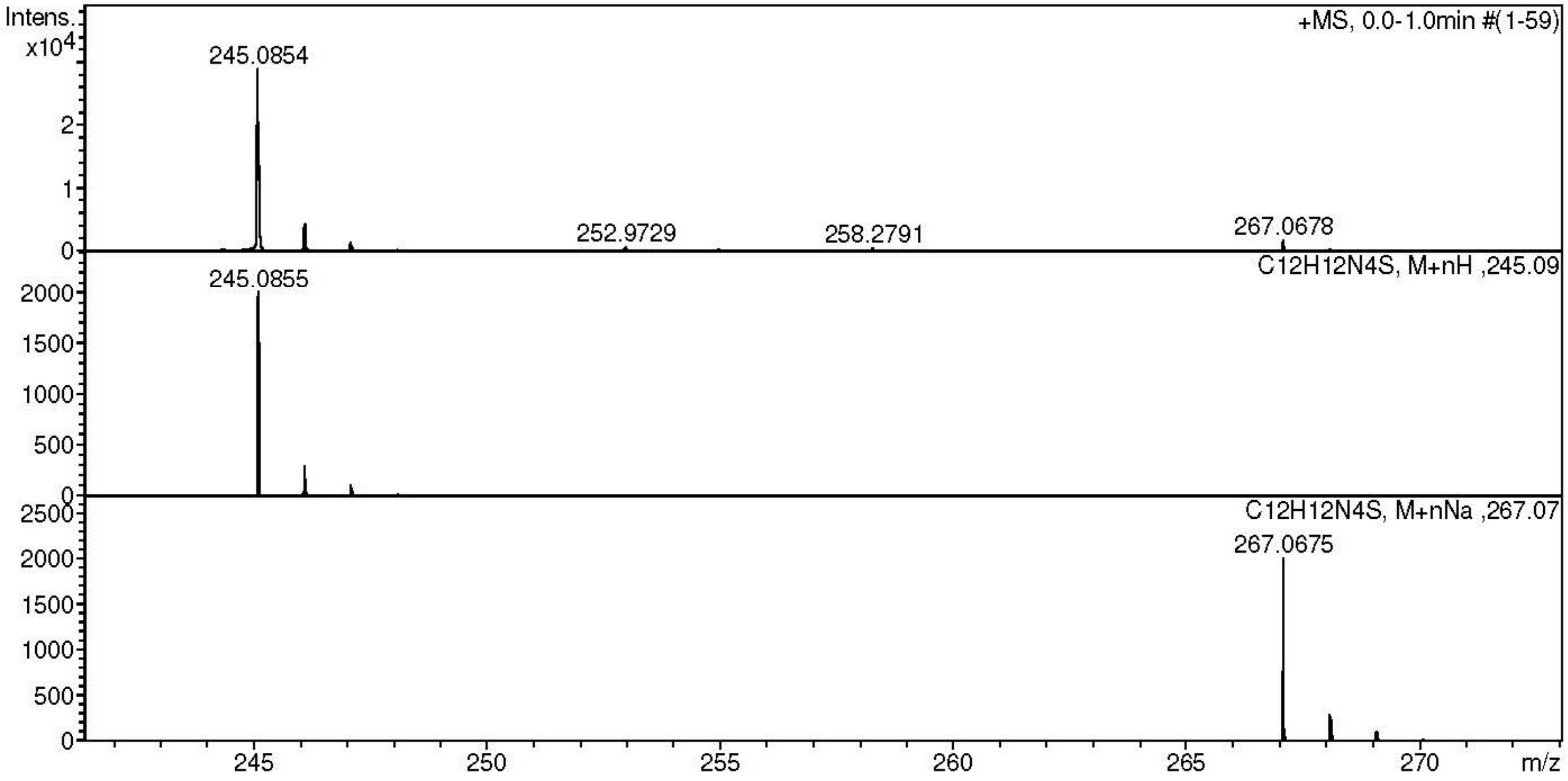

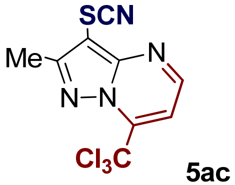


**5ac**

**$^1$H NMR**
**300.1 MHz**
**$CDCl_3$**

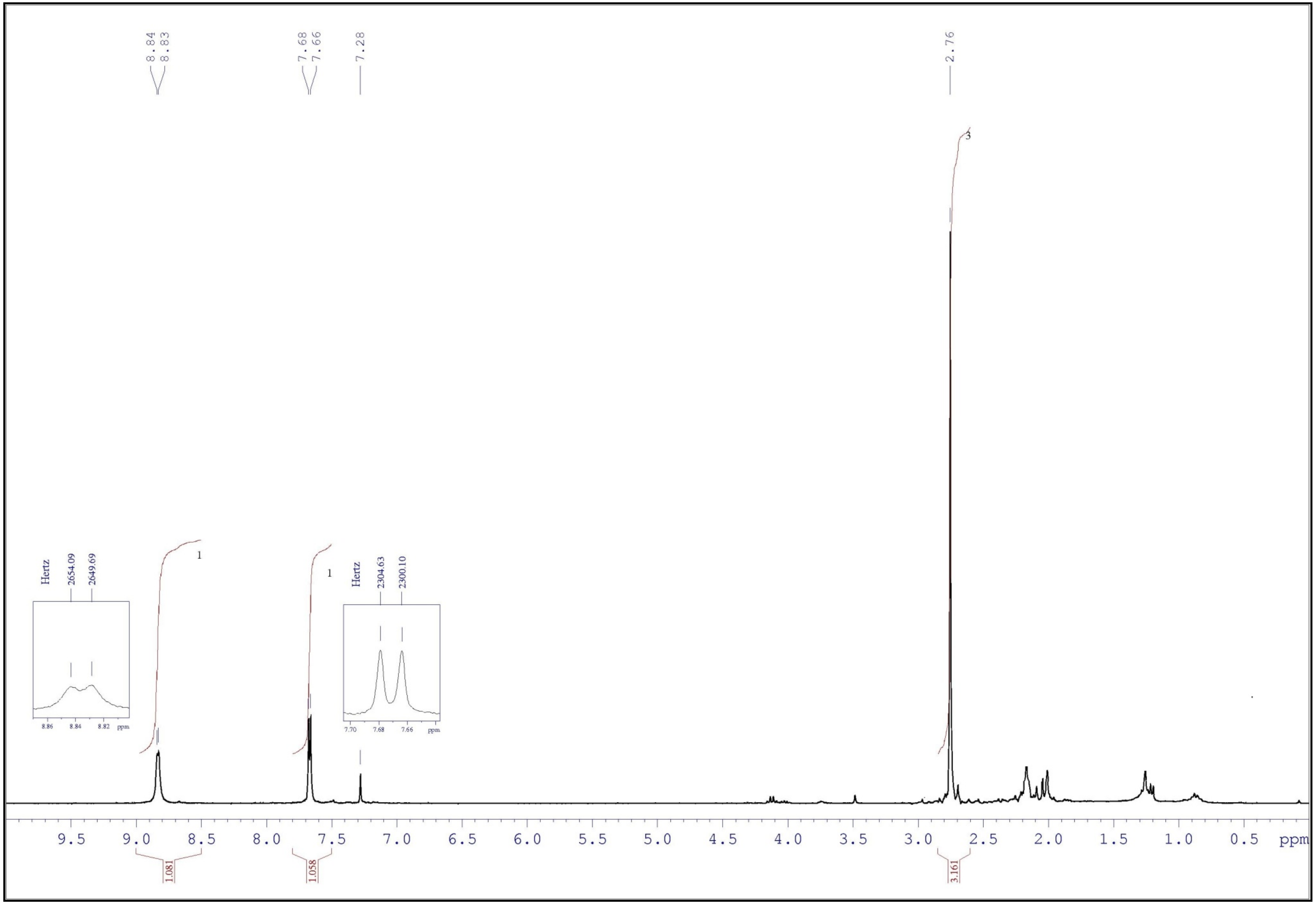

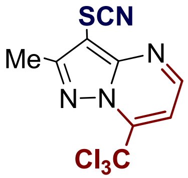


5ac

$^{13}$C NMR
75.5 MHz
$CDCl_3$

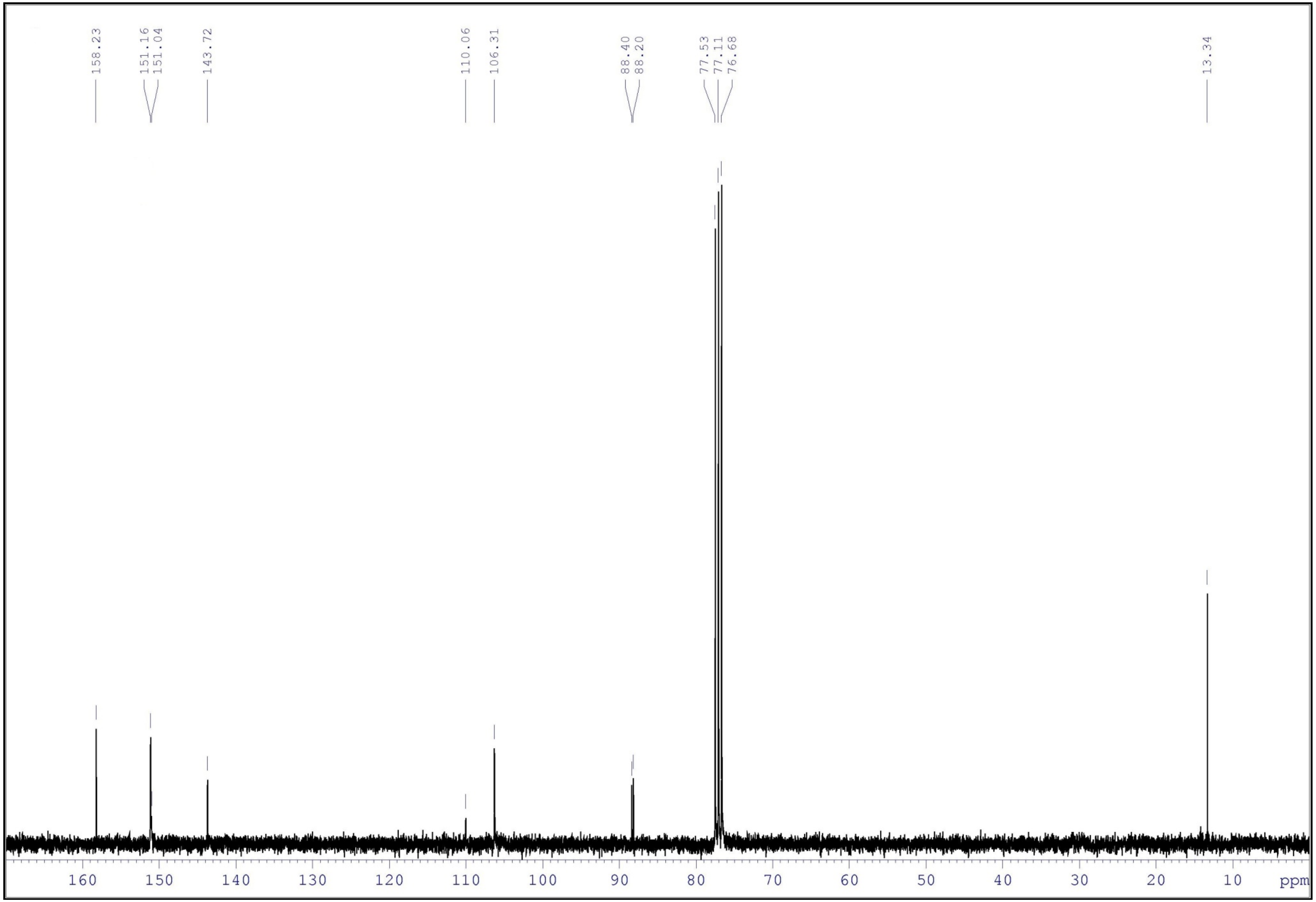

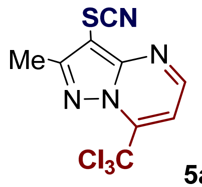


**5ac**

**HRMS (ESI)**

**Chemical Formula: $C_9H_5Cl_3N_4S$**

**Exact Mass: 305,93**

**Acquisition Parameter**

| | | | | | |
|---|---|---|---|---|---|
| Source Type | ESI | Ion Polarity | Positive | Set Nebulizer | 0.4 Bar |
| Focus | Not active | | | Set Dry Heater | 180 °C |
| Scan Begin | 50 m/z | Set Capillary | 4500 V | Set Dry Gas | 4.0 l/min |
| Scan End | 3000 m/z | Set End Plate Offset | -500 V | Set Divert Valve | Waste |

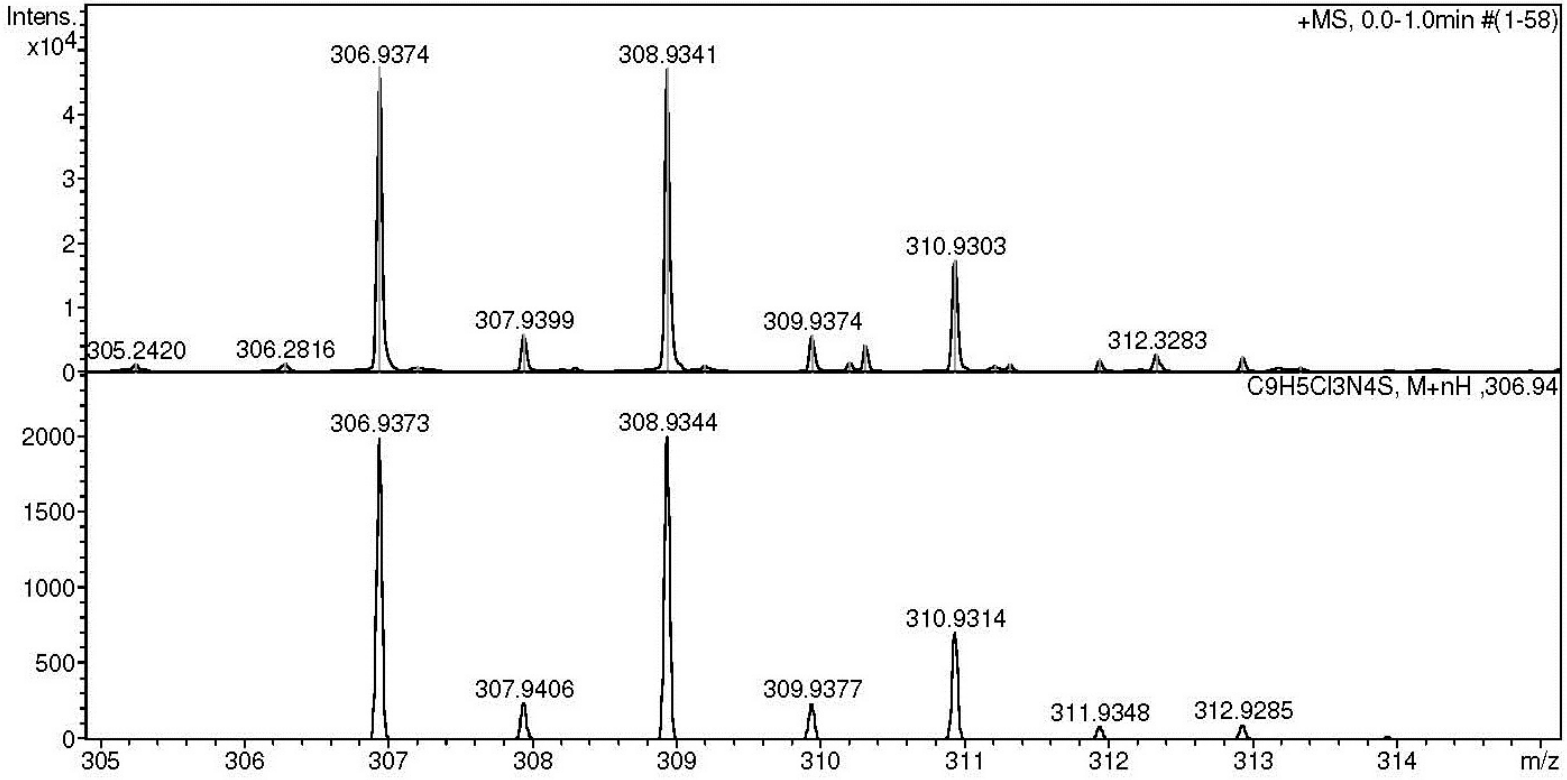

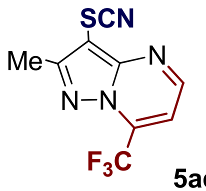

5ad

$^{1}$H NMR
300.1 MHz
$CDCl_3$

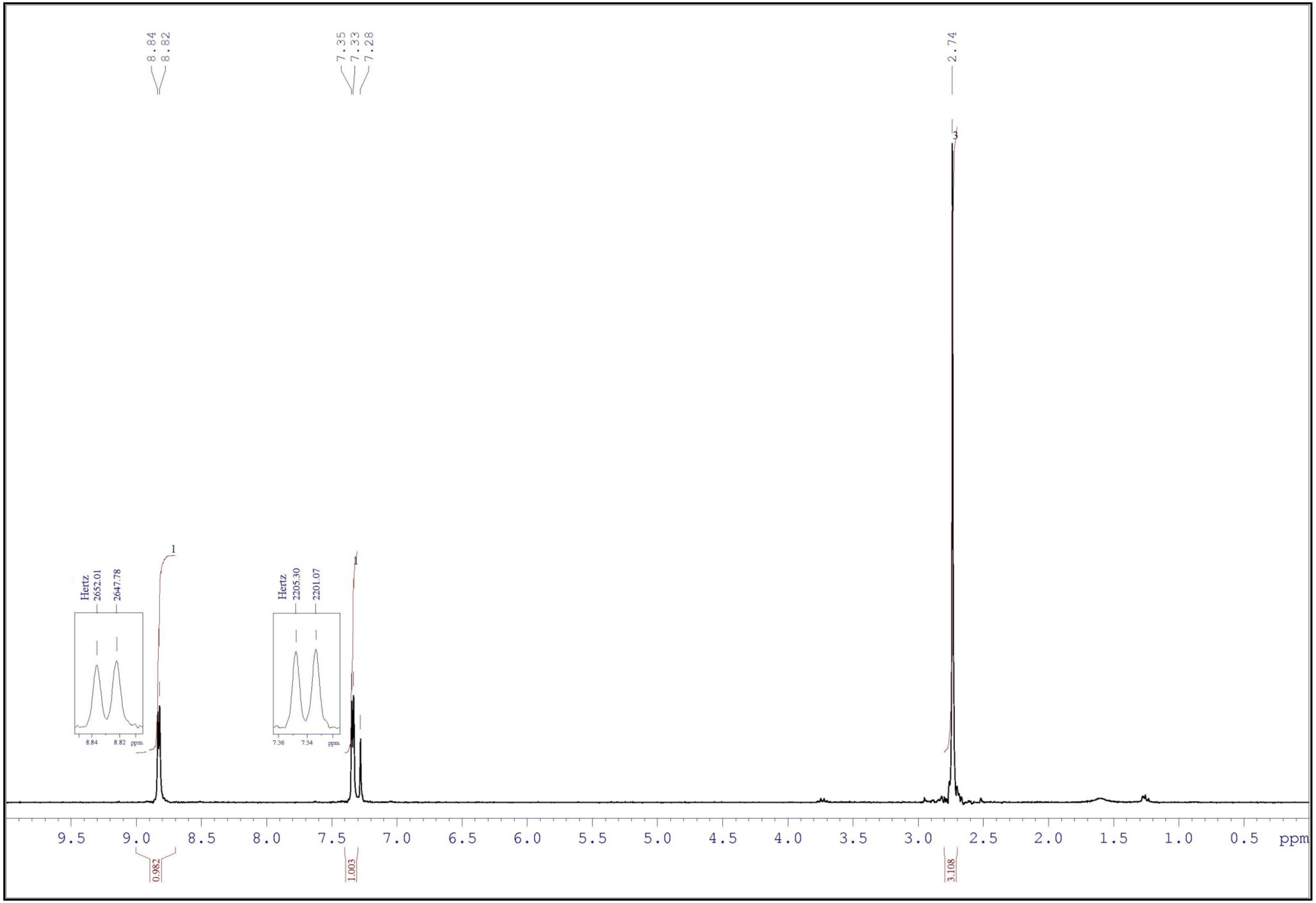

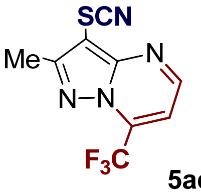

5ad

$^{13}C$ NMR
125.8 MHz
$CDCl_3$

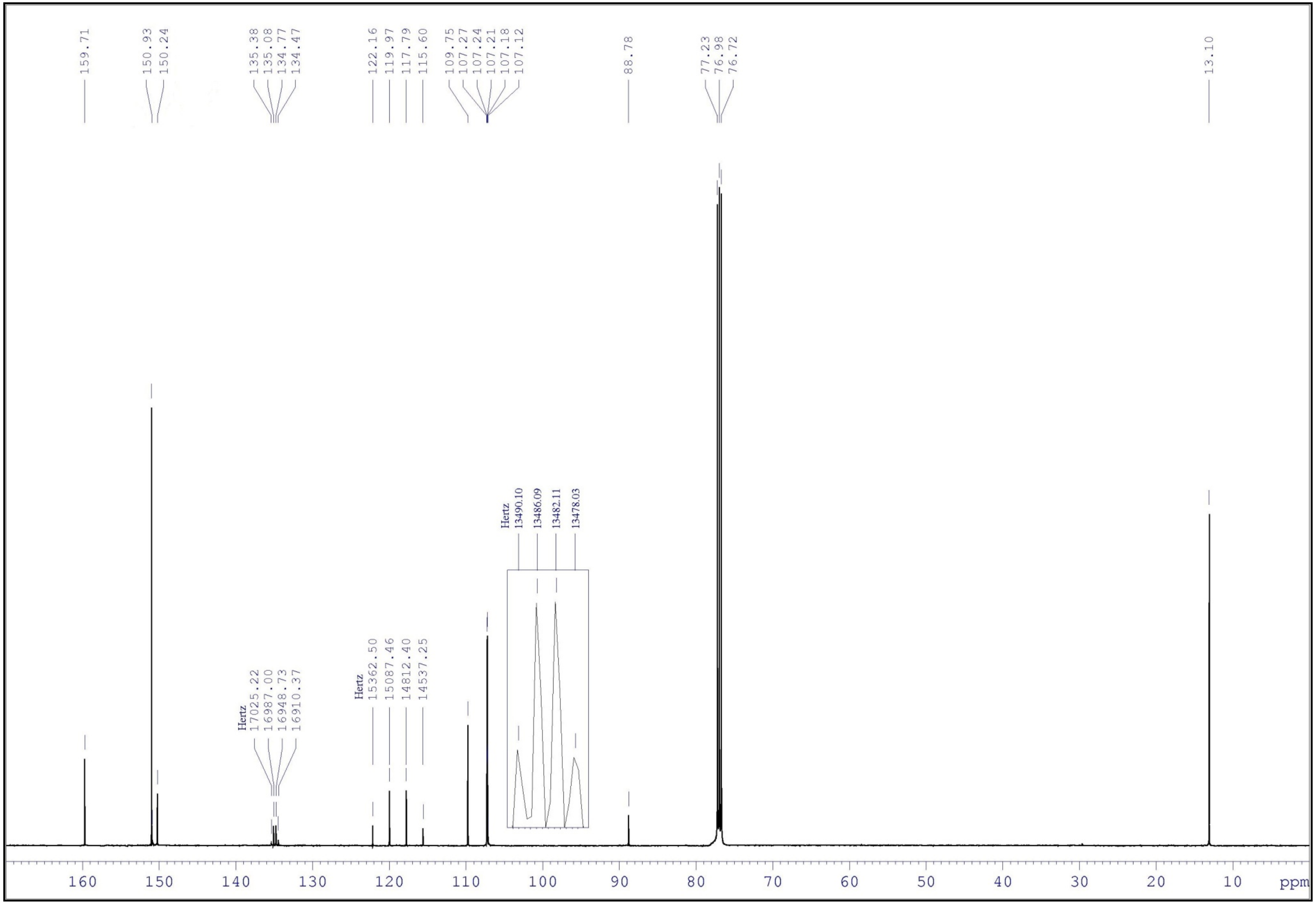

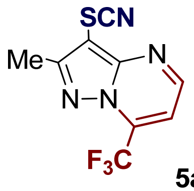


**5ad**

**HRMS (ESI)**

**Chemical Formula: $C_9H_5F_3N_4S$**

**Exact Mass: 258,02**

**Acquisition Parameter**

| | | | | | |
|---|---|---|---|---|---|
| Source Type | ESI | Ion Polarity | Positive | Set Nebulizer | 0.4 Bar |
| Focus | Not active | | | Set Dry Heater | 180 °C |
| Scan Begin | 50 m/z | Set Capillary | 4500 V | Set Dry Gas | 4.0 l/min |
| Scan End | 3000 m/z | Set End Plate Offset | -500 V | Set Divert Valve | Waste |

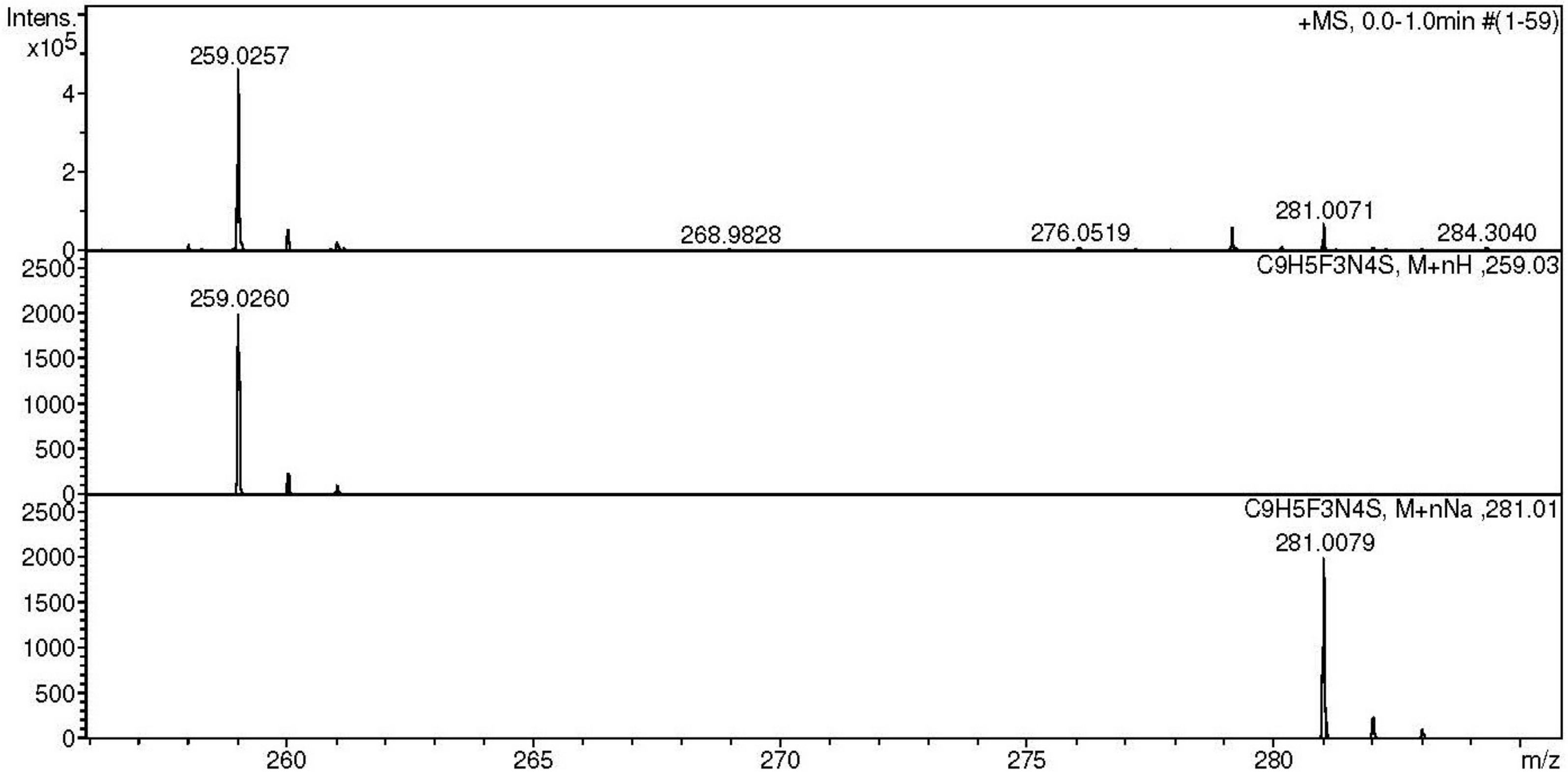

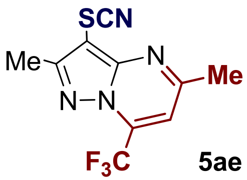


5ae

$^{1}H$ NMR

300.1 MHz

$CDCl_3$

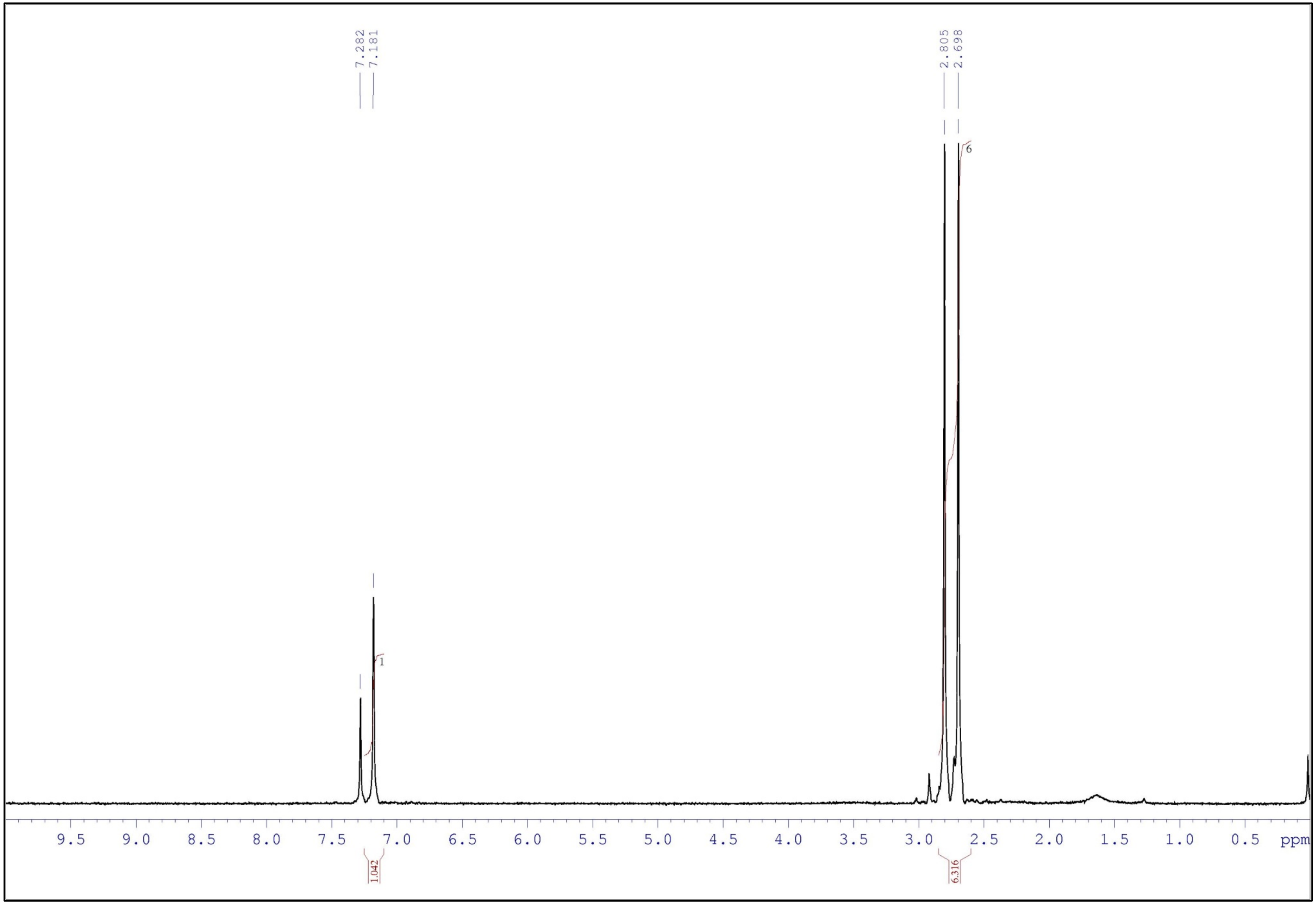

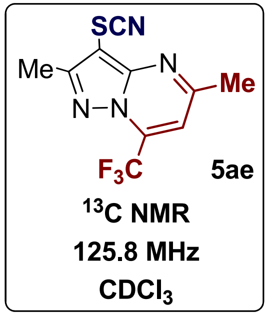
SCN
Me
N
Me
N–N
F3C
5ae
13C NMR
125.8 MHz
CDCl3

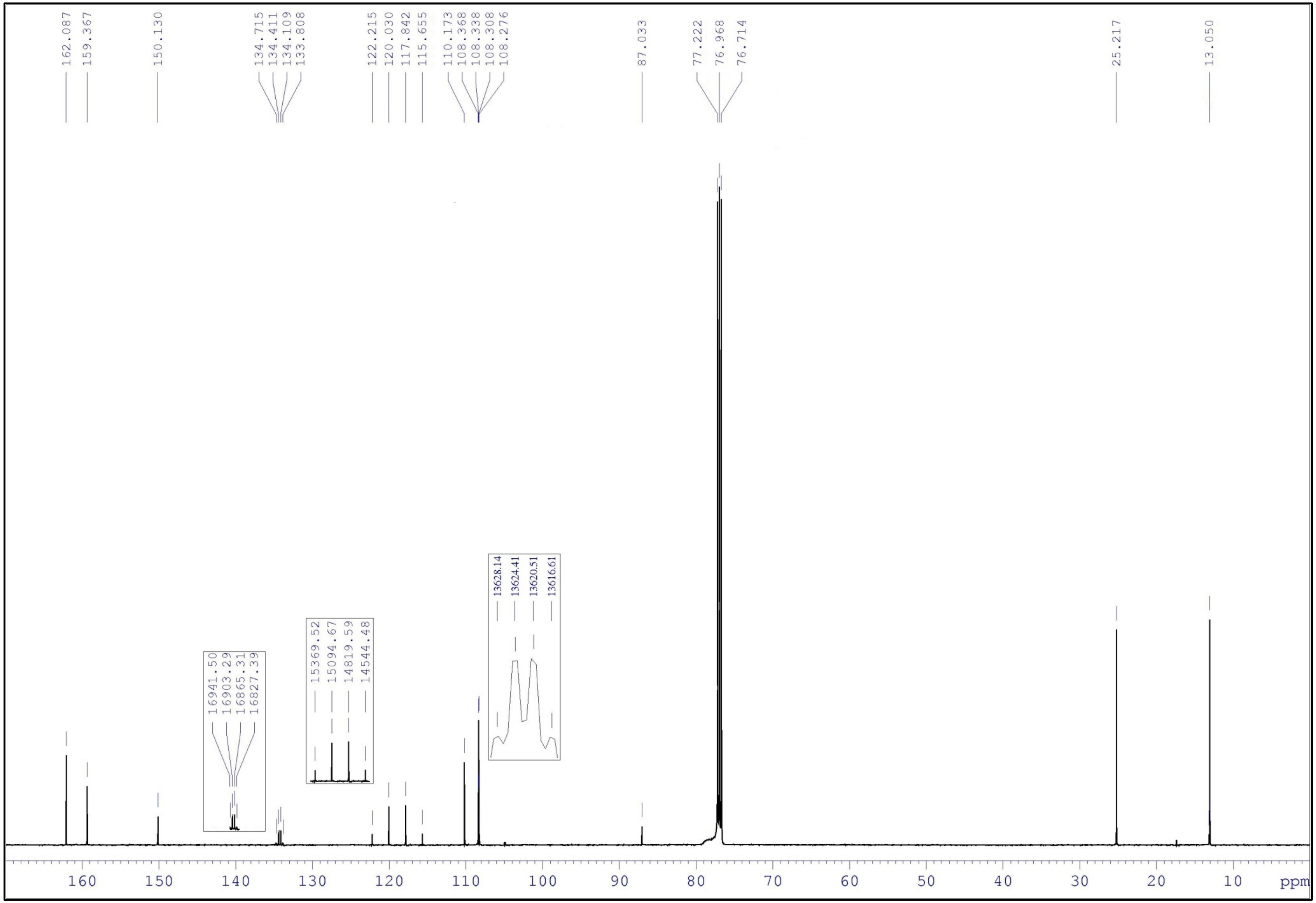
162.087
159.367
150.130
134.715
134.411
134.109
133.808
122.215
120.030
117.842
115.655
110.173
108.368
108.338
108.308
108.276
87.033
77.222
76.968
76.714
25.217
13.050
16941.50
16903.29
16865.31
16827.39
15369.52
15094.67
14819.59
14544.48
13628.14
13624.41
13620.51
13616.61
160
150
140
130
120
110
100
90
80
70
60
50
40
30
20
10
ppm

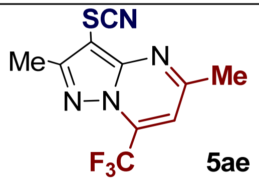


**5ae**

**HRMS (ESI)**

**Chemical Formula: $C_{10}H_7F_3N_4S$**

**Exact Mass: 272,03**

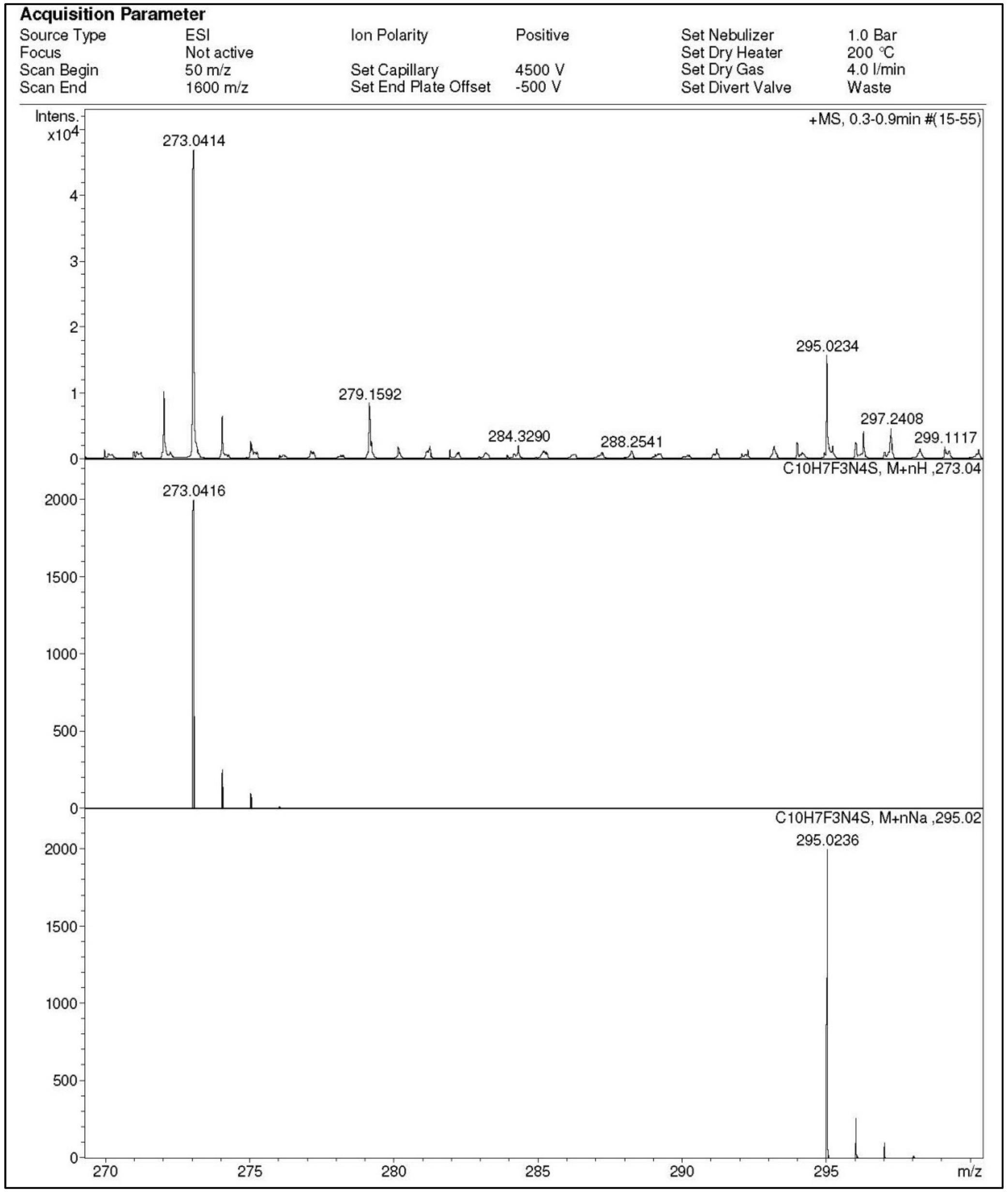

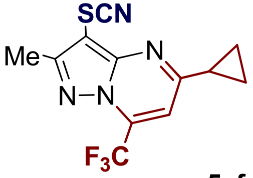


**5af**

$^1$H NMR

300.1 MHz

DMSO-$d_6$

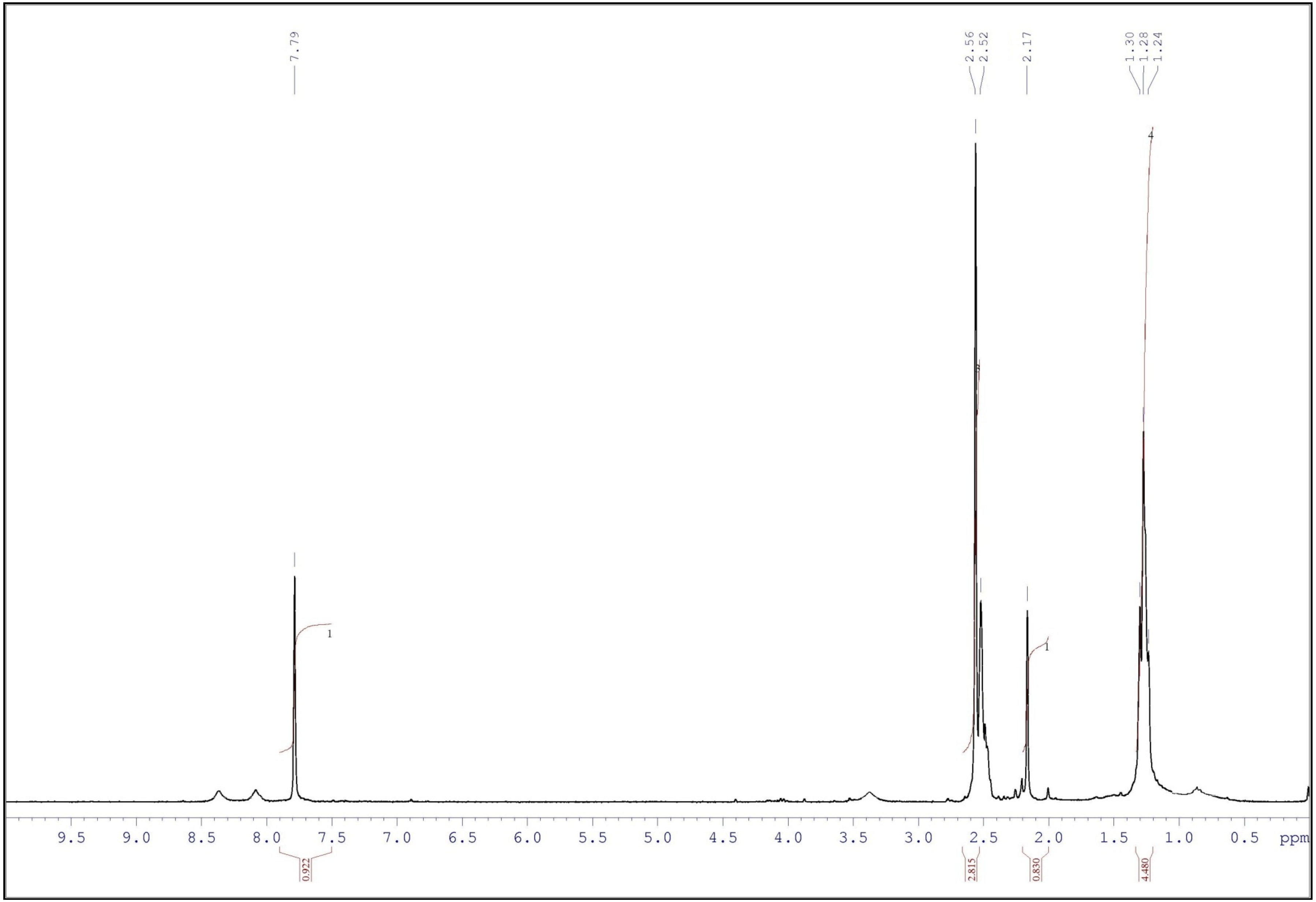

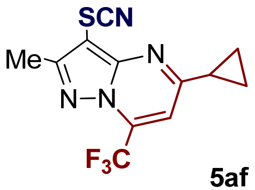


5af

$^{13}C$ NMR
125.8 MHz
DMSO-$d_6$

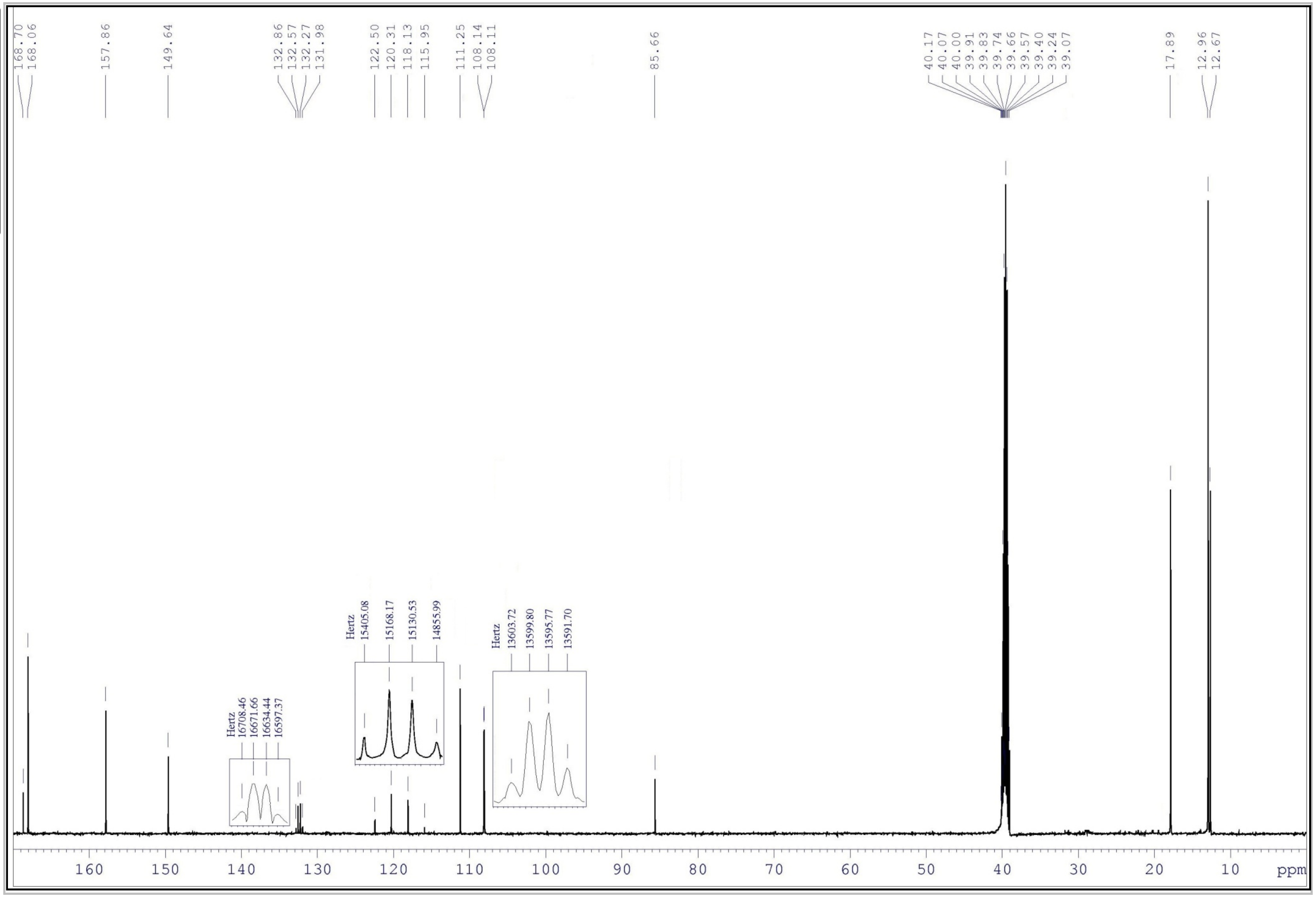

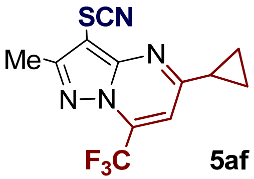


**5af**

**HRMS (ESI)**
**Chemical Formula: $C_{12}H_9F_3N_4S$**
**Exact Mass: 298,05**

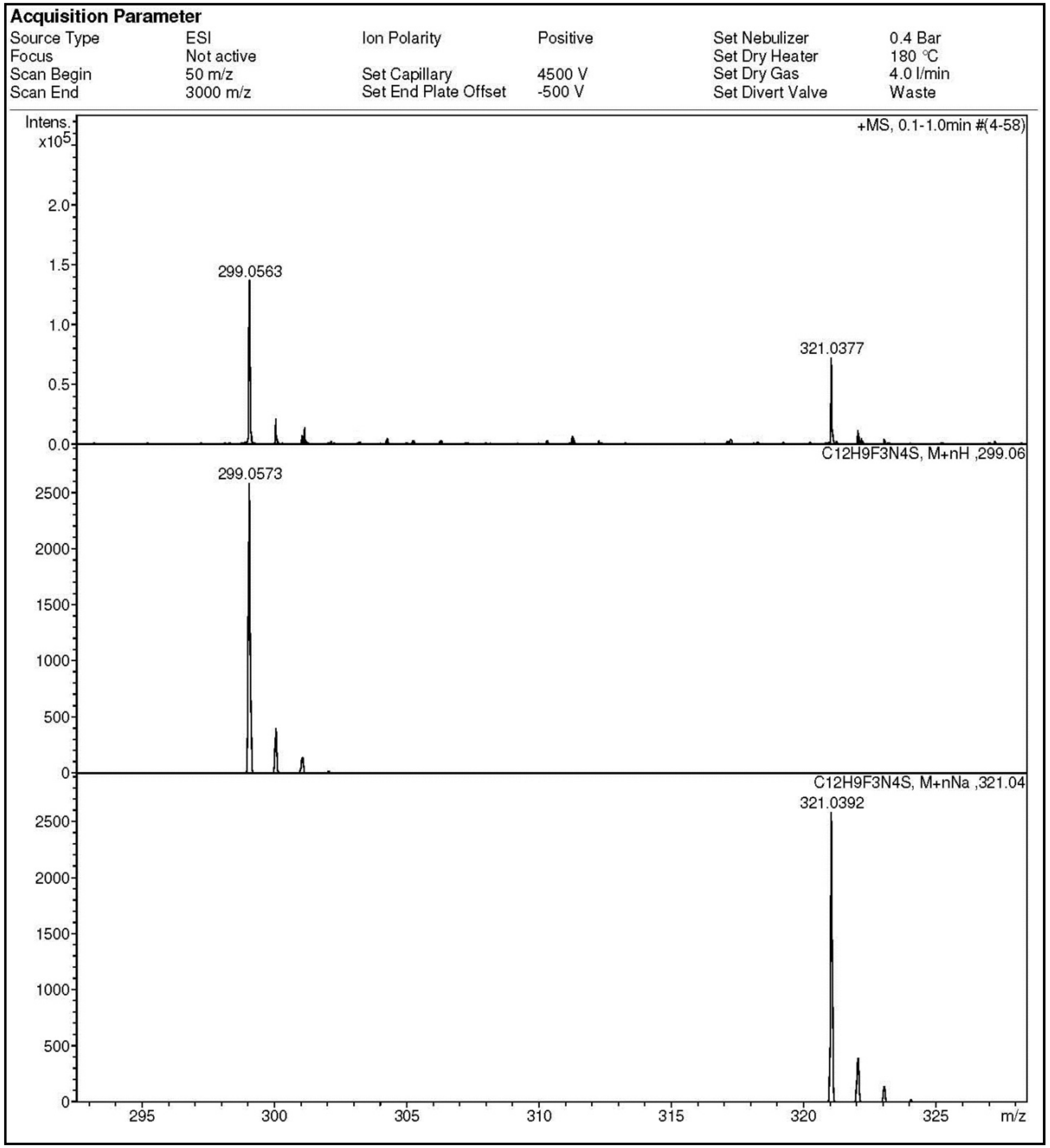

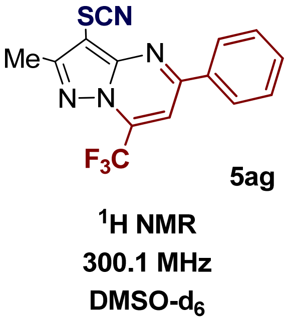


**5ag**

**$^1$H NMR**

**300.1 MHz**

**DMSO-$d_6$**

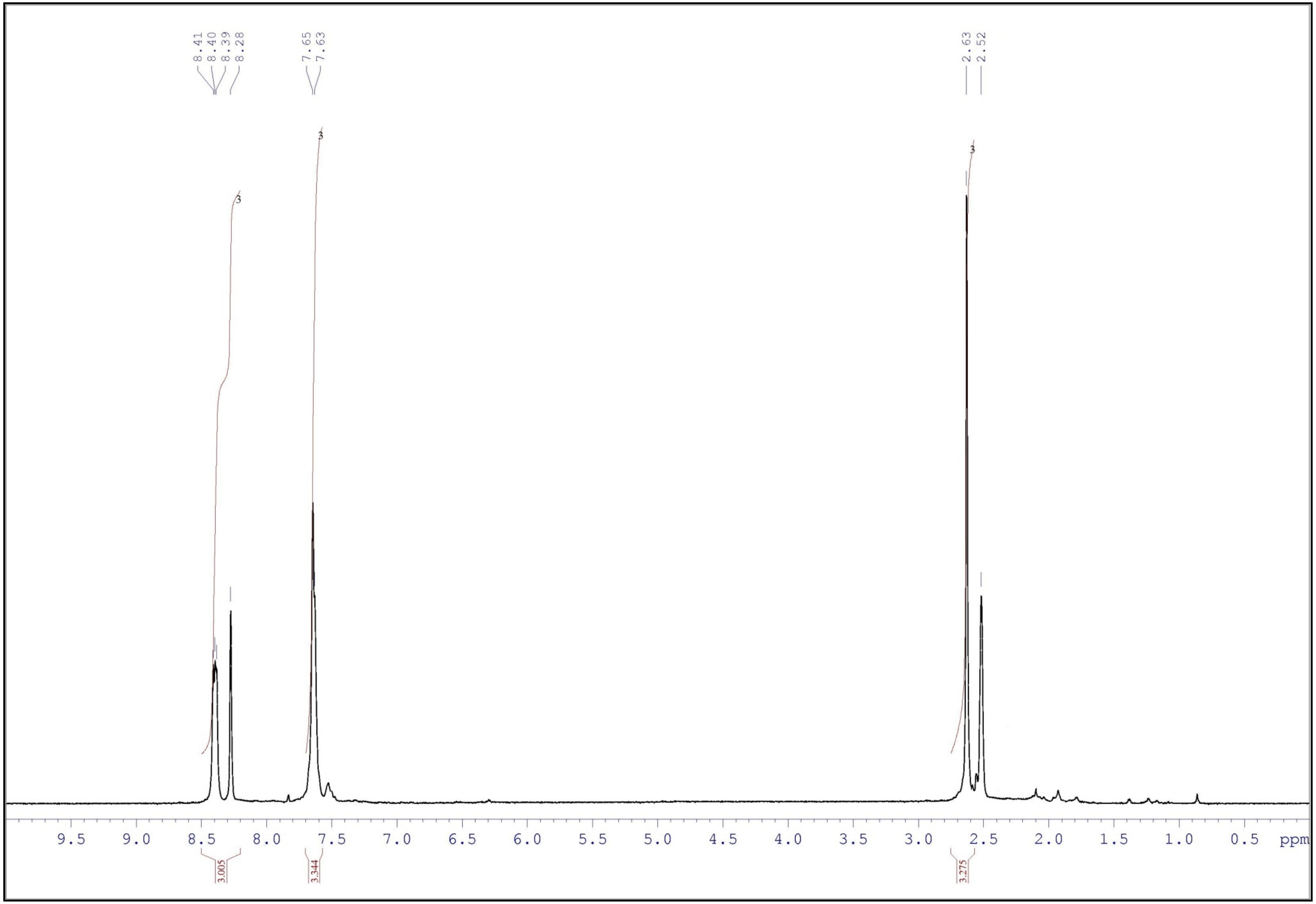

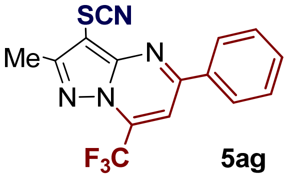

**5ag**

**$^{13}$C NMR**
**75.5 MHz**
**DMSO-$d_6$**

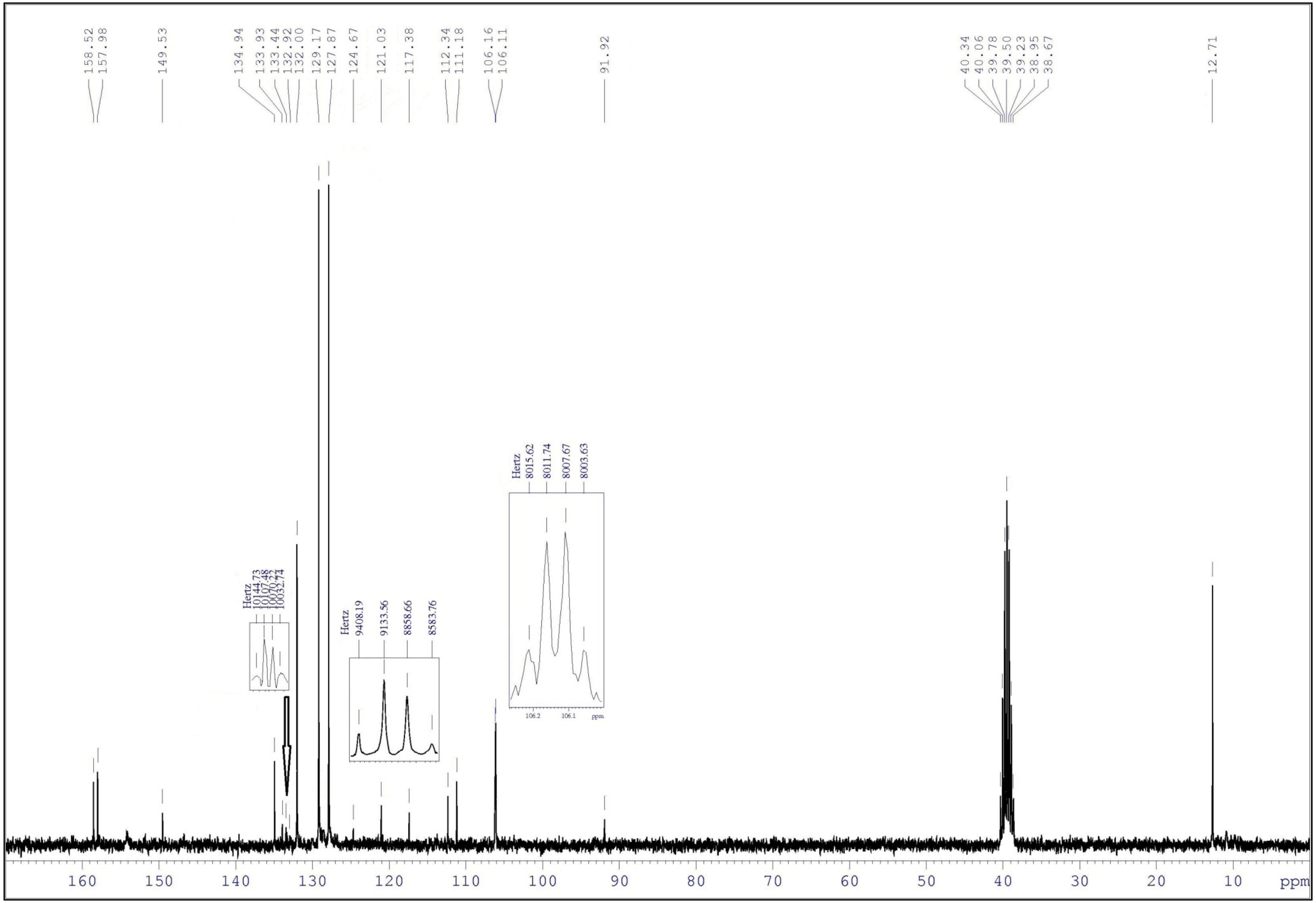

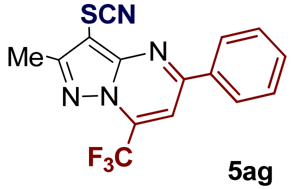


**5ag**

**HRMS (ESI)**

**Chemical Formula: $C_{15}H_9F_3N_4S$**

**Exact Mass: 334,05**

**Acquisition Parameter**

| | | | | | |
|---|---|---|---|---|---|
| Source Type | ESI | Ion Polarity | Positive | Set Nebulizer | 0.4 Bar |
| Focus | Not active | | | Set Dry Heater | 180 °C |
| Scan Begin | 50 m/z | Set Capillary | 4500 V | Set Dry Gas | 4.0 l/min |
| Scan End | 3000 m/z | Set End Plate Offset | -500 V | Set Divert Valve | Waste |

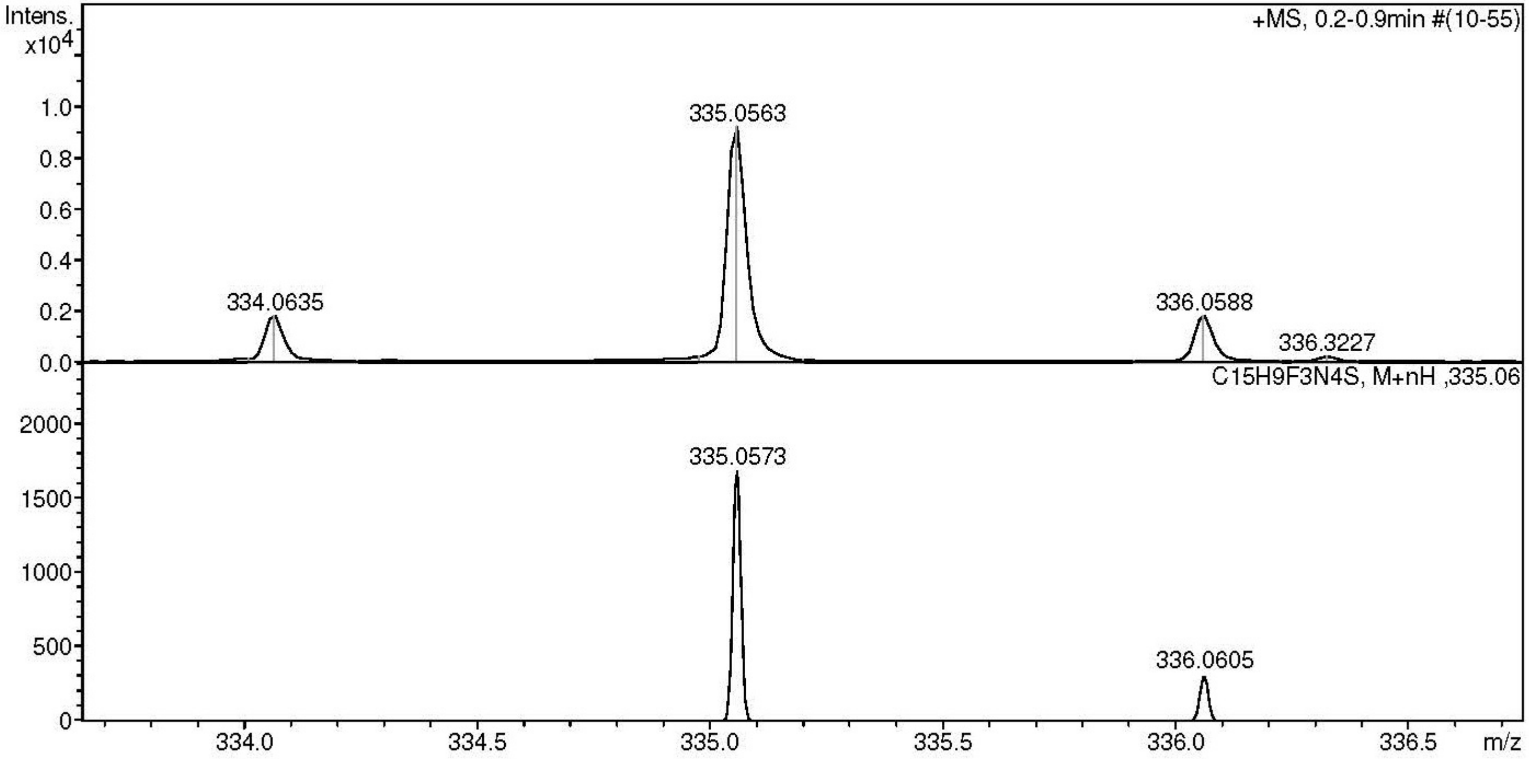

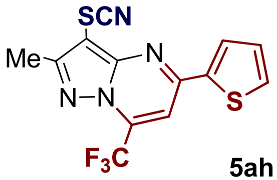


**5ah**

**$^1$H NMR**
**300.1 MHz**
**$CDCl_3$**

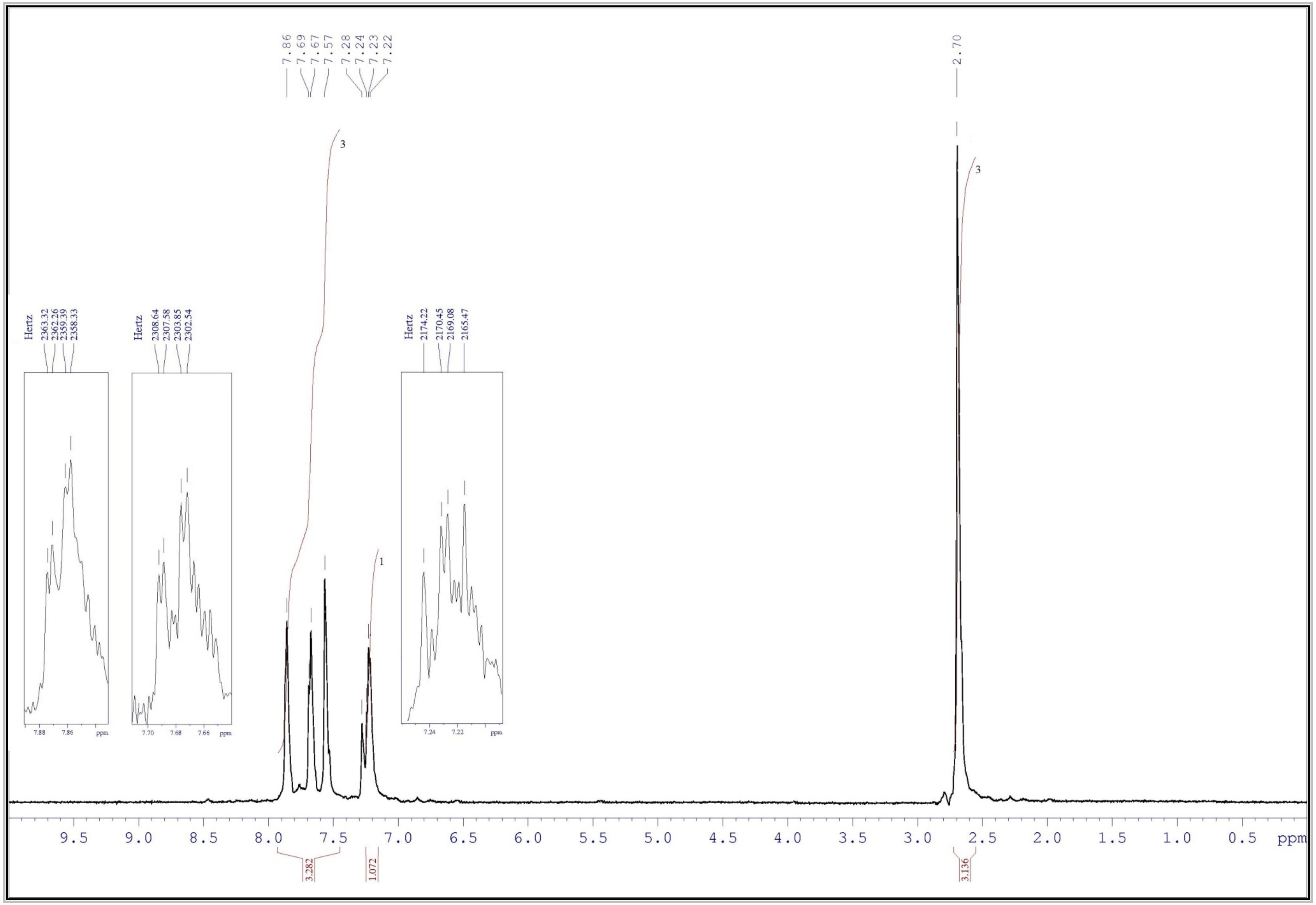

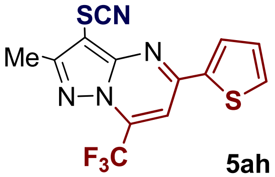


**5ah**

$^{13}C$ NMR
75.5 MHz
$CDCl_3$

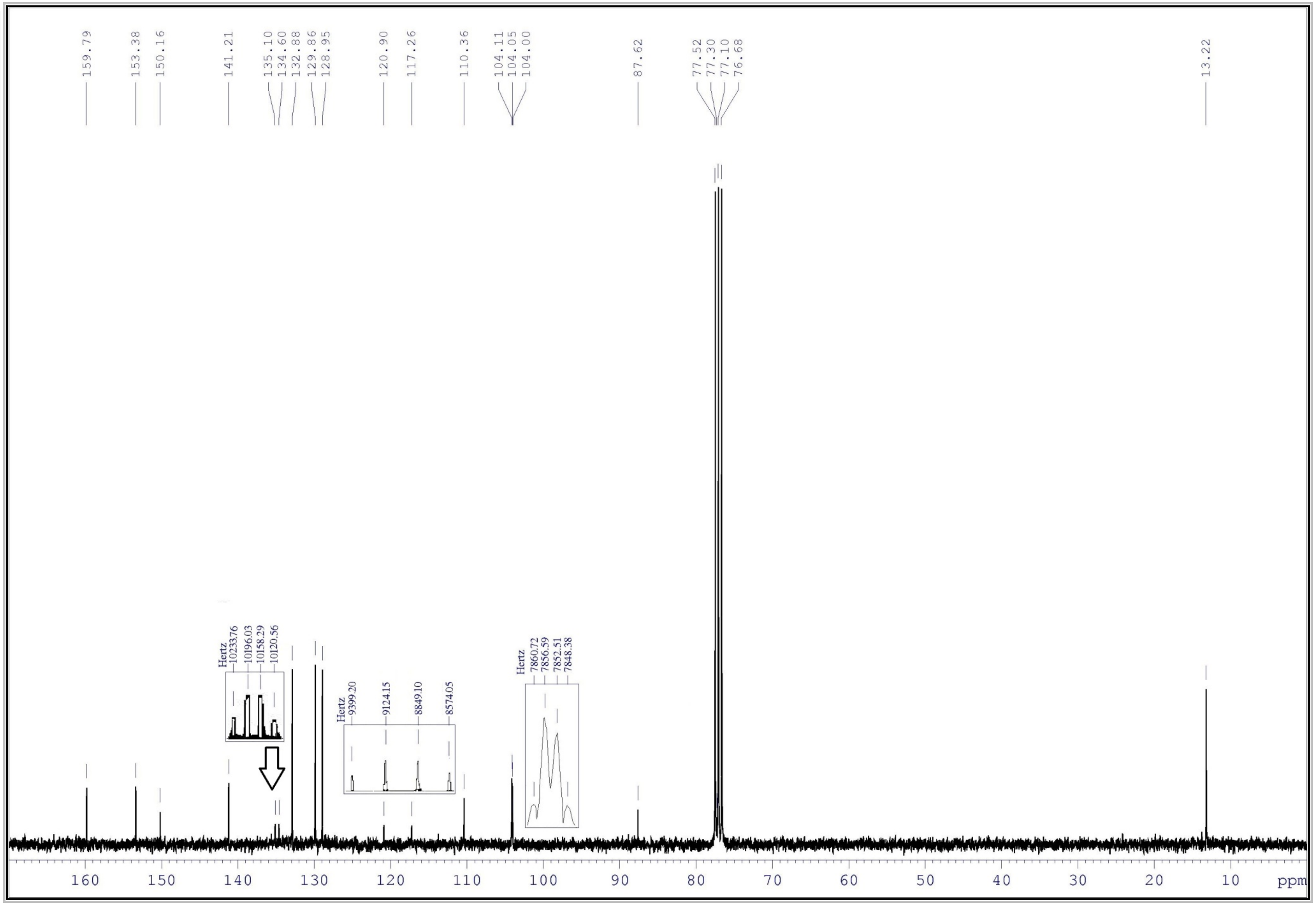

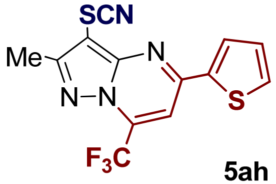


**5ah**

**HRMS (ESI)**

**Chemical Formula: $C_{13}H_7F_3N_4S_2$**

**Exact Mass: 340,01**

**Acquisition Parameter**

| | | | | | | | |
|---|---|---|---|---|---|---|---|
| Source Type | ESI | Ion Polarity | Positive | | | Set Nebulizer | 0.4 Bar |
| Focus | Not active | | | | | Set Dry Heater | 180 °C |
| Scan Begin | 50 m/z | Set Capillary | 4500 V | | | Set Dry Gas | 4.0 l/min |
| Scan End | 3000 m/z | Set End Plate Offset | -500 V | | | Set Divert Valve | Waste |

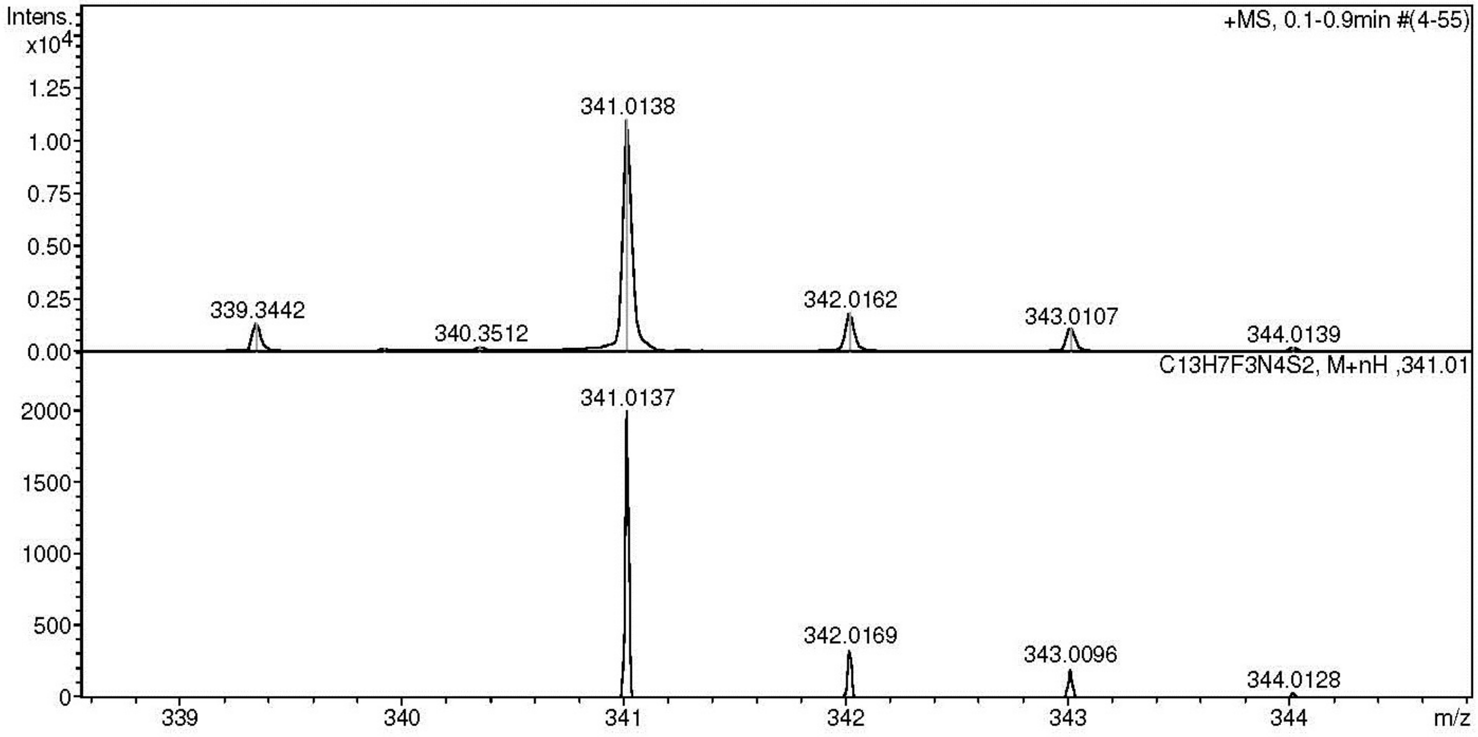

Supplement: Supplementary file 1 [file molecules-25-04169-s001.pdf]
